# Boosted charge and proton transfer over ternary Co/Co_3_O_4_/CoB for electrochemical nitric oxide reduction to ammonia

**DOI:** 10.1038/s41467-025-60043-6

**Published:** 2025-05-26

**Authors:** Xiaoxuan Fan, Zhenyuan Teng, Lupeng Han, Yongjie Shen, Xiyang Wang, Wenqiang Qu, Jialing Song, Zhenlin Wang, Haiyan Duan, Yimin A. Wu, Bin Liu, Dengsong Zhang

**Affiliations:** 1 Innovation Institute of Carbon Neutrality, International Joint Laboratory of Catalytic Chemistry, State Key Laboratory of Advanced Special Steel, Department of Chemistry, College of Sciences, Shanghai University, Shanghai, China; 2 Department of Materials Science and Engineering, City University of Hong Kong, Hong Kong SAR, China; 3 Institute for Chemical Reaction Design and Discovery (WPI-ICReDD), Hokkaido University, Sapporo, Japan; 4 Department of Mechanical and Mechatronics Engineering, Waterloo Institute for Nanotechnology, University of Waterloo, ON Waterloo, Canada; 5 Department of Chemistry, Hong Kong Institute of Clean Energy (HKICE) & Center of Super-Diamond and Advanced Films (COSDAF), City University of Hong Kong, Hong Kong SAR, China

**Keywords:** Electrocatalysis, Electrocatalysis, Electrocatalysis, Pollution remediation

## Abstract

The electrochemical nitric oxide reduction reaction (NORR) holds a great potential for removing environmental pollutant NO and meanwhile generating high value-added ammonia (NH_3_). Herein, we tactfully design and synthesize a ternary Co/Co_3_O_4_/CoB heterostructure that displays a high NH_3_ Faradaic efficiency of 98.8% in NORR with an NH_3_ yield rate of 462.18 µmol cm^−2^ h^−1^ (2.31 mol h^−1^ g_cat_^−1^) at −0.5 V versus reversible hydrogen electrode, outperforming most of the reported NORR electrocatalysts to date. The superior NORR performance is attributed to the enhanced charge and proton transfer over the ternary Co/Co_3_O_4_/CoB heterostructure. The charge transfer between CoB and Co/Co_3_O_4_ yields electron-deficient Co and electron-rich Co_3_O_4_. The electron-deficient Co sites boost H_2_O dissociation to generate *H while the electron-rich low-coordination Co_3_O_4_ sites promote NO adsorption. The *H formed on electron-deficient Co sites is more favorable to transfer to electron-rich Co_3_O_4_ sites adsorbed with NO, facilitating the selective hydrogenation of NO. This study paves the way for designing and developing highly efficient electrocatalysts for electrochemical reduction of NO to NH_3_.

## Abstract (web summary)

The electrochemical NH_3_ synthesis from NO reduction faces challenges, including weak NO adsorption and sluggish proton supply. Here, the authors report a ternary Co/Co_3_O_4_/CoB heterostructure promotes NO adsorption while boosting H_2_O dissociation and subsequent hydrogenation to produce NH3.

## Introduction

Ammonia (NH_3_) is an indispensable chemical for agriculture, chemical industry, refrigerant, hydrogen carrier, and healthcare^1–5^. Unfortunately, NH_3_ production by traditional Haber-Bosch method requires harsh conditions of high temperature and high pressure, emitting a large quantity of greenhouse gases^6,7^. One of the alternatives to the Haber-Bosch method for NH_3_ synthesis is the electrocatalytic reduction of nitrogen-containing species, including nitrogen gas (N_2_), nitric oxide (NO), nitrite (NO_2_^−^), and nitrate (NO_3_^−^)^8–11^. The nitrogen reduction reaction (NRR) suffers from low NH_3_ Faradaic efficiency (FE_NH3_) and NH_3_ yield rate because of the high dissociation energy (941 kJ mol^−1^) of N ≡ N and the competitive hydrogen evolution reaction (HER) (the reduction potential of N_2_ (0.093 V vs. RHE) and H_2_O (0 V vs. RHE) are close to each other)^12–14^. The electroreduction of NO_2_^−^ and NO_3_^−^ to NH_3_ (NO_2_^−^RR and NO_3_^−^RR) have more complicated reaction pathways, and generate more types of N-containing by-products^15,16^. Compared with NRR, the electrochemical NO reduction reaction (NORR) is kinetically and thermodynamically more favorable because NO possesses a lower dissociation energy (204 kJ mol^−1^) and a more positive reduction potential (0.71 V vs. RHE)^17–19^. The approach to couple NO oxidation with NO_3_^−^ reduction is also considered as an alternative to NORR. But NORR to produce NH_3_ generally requires lower energy consumption^20,21^. In recent years, NORR is becoming increasingly attractive because NORR not only can remove environmental pollutant NO from industrial exhaust gas, but also produces value-added chemicals.

The electrochemical reduction of NO involves chemical activation of NO followed by NO hydrogenation^22–24^. Currently, the FE_NH3_ and NH_3_ yield rate in electrochemical NORR are still impeded by the weak adsorption of NO and the sluggish proton supply. To improve the NORR performance, researchers have made endeavors to enhance the adsorption of NO by transition metal oxide modification or vacancy engineering^25,26^. The proton supply could be enhanced by using metal-based electrocatalysts that would facilitate H_2_O dissociation^24,27^. Constructing single atoms in amorphous metal oxides with oxygen vacancies to form metal-O moieties could accelerate hydrogenation of NO to produce NH₃^28–30^. However, till now, it is still intractable to acquire both high FE_NH3_ and NH_3_ yield rate simultaneously because of the complex balance of the NO adsorption and H_2_O dissociation. Early studies have shown fairly high NO adsorption capacity over Co_3_O_4_ surface^31–34^. However, the poor activation and dissociation of H_2_O on Co_3_O_4_ greatly restricts the hydrogenation reaction in NORR^35^. Metallic Co on Co_3_O_4_ heterostructure is designed to promote H_2_O dissociation to increase available *H^36^. But this concurrently accelerates HER, leading to a low NORR FE_NH3_ and NH_3_ yield rate^27,30,37–39^. Tuning charge distribution on catalyst’s surface is able to directionally drive *H transfer to enhance NO hydrogenation during NORR^40,41^. Boron (B) possesses a 2s^2^p^1^ electronic structure, which exhibits flexible valence states during catalytic reactions. Introducing B into the Co/Co_3_O_4_ heterostructure is anticipated to optimize NO adsorption and H_2_O dissociation, and in the meantime induce *H transfer to promote NO hydrogenation and suppress HER during NORR, thus greatly enhancing NORR activity and selectivity.

In this study, we tactfully designed and synthesized a ternary Co/Co_3_O_4_/CoB heterostructure by reducing Co_3_O_4_ with NaBH_4_. The ternary Co/Co_3_O_4_/CoB catalyst exhibited an excellent electrochemical NORR performance, achieving an NH_3_ yield rate of 462.18 µmol cm^−2^ h^−1^ (2.31 mol h^−1^ g_cat_^−1^) and an FE_NH3_ of 98.80% at −0.5 V vs. RHE, outperforming most of the reported NORR electrocatalysts to date. To demonstrate the application potential, a Zn-NO battery was assembled using the Co/Co_3_O_4_/CoB as the cathode, which delivered a high power density of 10.06 mW cm^−2^. A series of experiments and density functional theory (DFT) calculations revealed that the enhanced charge and proton transfer over the ternary Co/Co_3_O_4_/CoB effectively boosted the electrochemical NORR. In detail, the charge transfer between CoB and Co/Co_3_O_4_ yielded electron-deficient Co and electron-rich Co_3_O_4_. The electron-deficient Co sites boosted H_2_O dissociation to generate *H, while the electron-rich low-coordination Co_3_O_4_ sites promoted NO adsorption and *H transfer. Thanks to the enhanced charge and proton transfer, the energy barrier of the NORR potential-determining step from *NO to *HNO over the ternary Co/Co_3_O_4_/CoB was greatly reduced. Furthermore, the Co/Co_3_O_4_/CoB could also facilitate adsorption of *NH and *NH_2_, beneficial for NO reduction to produce NH_3_.

## Results

### Catalyst synthesis and structure characterization

**Fig. 1:**
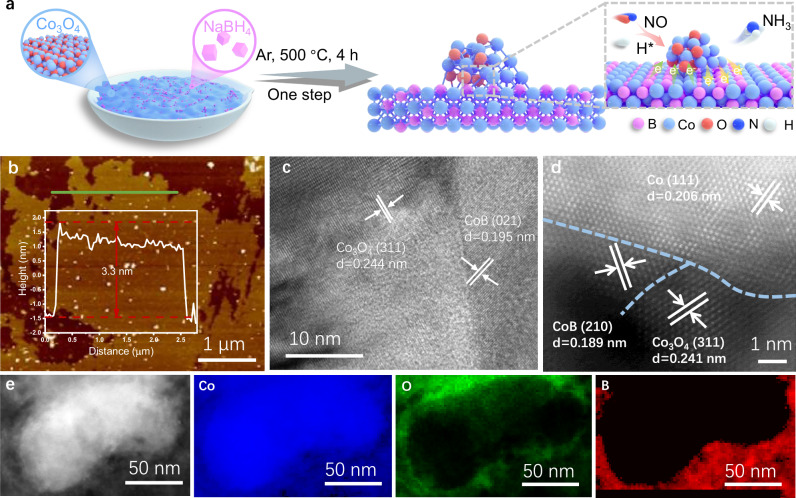
Synthesis and morphology of Co/Co_3_O_4_/CoB. **a** Schematic diagram showing the synthesis process of Co/Co_3_O_4_/CoB. **b** AFM image of Co/Co_3_O_4_/CoB. The inset shows the height profile along the red line in (**b**). **c** HRTEM image of Co/Co_3_O_4_/CoB. **d** Aberration-corrected HAADF-STEM image of Co/Co_3_O_4_/CoB. **e** HAADF-STEM image and the corresponding EELS mappings of Co, O, and B elements in Co/Co_3_O_4_/CoB. Source data for Fig. 1 are provided as a Source Data file.

The ternary Co/Co_3_O_4_/CoB heterostructured electrocatalyst was synthesized by reducing Co_3_O_4_ using NaBH_4_ in an Ar atmosphere at 500 °C for 4 h (Fig. 1a). By applying the same method, Co/Co_3_O_4_ could also be obtained by decreasing the quality ratio of NaBH_4_/Co_3_O_4_. The as-prepared Co/Co_3_O_4_/CoB shows the morphology of nanoparticles supported on nanosheets based on the transmission electron microscope (TEM, Supplementary Fig. 1) measurement. The average thickness of the nanosheets is ∼3.3 nm determined by atomic force microscopy (AFM, Fig. 1b). The nanoparticles on the nanosheets are composed of large nanoparticles surrounded by many small nanoparticles (HRTEM, Supplementary Fig. 2). Co/Co_3_O_4_/CoB shows Co_3_O_4_ (311) and CoB (021) crystalline phases from the HRTEM image (Fig. 1c). To precisely determine the structure of Co/Co_3_O_4_/CoB, aberration-corrected HAADF-STEM measurement was further performed. As shown in Fig. 1d, abundant interfaces among Co, Co_3_O_4_, and CoB phases can be clearly observed, suggesting the formation of a ternary Co/Co_3_O_4_/CoB heterostructure. The electron energy loss spectroscopy (EELS) mapping (Fig. 1e) combined with the HRTEM image (Supplementary Fig. 2) clarifies that the Co/Co_3_O_4_/CoB heterostructure is composed of a CoB nanosheet supported with large Co and small Co_3_O_4_ nanoparticles. For comparison, Co/Co_3_O_4_ shows a heterostructure of Co nanoparticles supported on Co_3_O_4_ nanosheets (Supplementary Figs. 3 and 4).

**Fig. 2:**
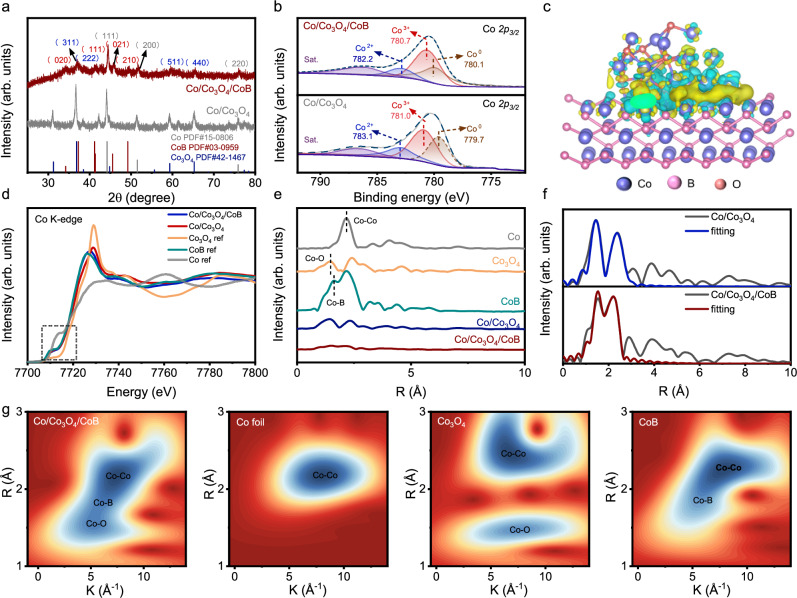
Structural features of Co/Co_3_O_4_/CoB. **a** XRD patterns of Co/Co_3_O_4_ and Co/Co_3_O_4_/CoB. **b** Co *2p*_*3/2*_ XPS spectra of Co/Co_3_O_4_ and Co/Co_3_O_4_/CoB. **c** The charge density difference analysis for Co/Co_3_O_4_/CoB. Yellow and blue colors represent electron accumulation and depletion regions, respectively. The isosurface value is 0.004 e/Å^3^. **d** Co K-edge XANES spectra of Co/Co_3_O_4_, Co/Co_3_O_4_/CoB, Co foil, CoB, and Co_3_O_4_. **e** The FT-EXAFS spectra of Co/Co_3_O_4_, Co/Co_3_O_4_/CoB, Co foil, CoB, and Co_3_O_4_. **f** FT-EXAFS fitting curves for Co/Co_3_O_4_ and Co/Co_3_O_4_/CoB in R space. **g** WT contour plots of Co/Co_3_O_4_/CoB, Co foil, CoB, and Co_3_O_4_. Source data for Fig. 2 are provided as a Source Data file.

The structure and valence state of Co/Co_3_O_4_/CoB were analyzed by X-ray powder diffraction (XRD) and X-ray photoelectron spectroscopy (XPS). As shown in Fig. 2a, the XRD patterns show clear diffraction peaks of Co_3_O_4_ (PDF #42-1467), Co (PDF #15-0806) and CoB (PDF #03-0959) for Co/Co_3_O_4_/CoB, and Co_3_O_4_ (PDF #42-1467) and Co (PDF #15-0806) for Co/Co_3_O_4_. The B *1s* XPS spectrum of Co/Co_3_O_4_/CoB also evidences the formation of CoB (Supplementary Fig. 5). In Fig. 2b, the Co *2p*_*3/2*_ XPS spectrum of Co/Co_3_O_4_ exhibits three peaks at binding energies of 779.7, 781.0 and 783.1 eV, which can be attributed to Co^0^, Co^3+^ and Co^2+^, respectively^42–44^. For Co/Co_3_O_4_/CoB, the Co^0^ peak slightly shifts to a higher binding energy (780.1 eV), while the Co^3+^ (780.7 eV) and Co^2+^ (782.2 eV) peaks shift toward lower binding energies as compared to those of Co/Co_3_O_4_. Because Co^0^ and Co^3+^/Co^2+^ mainly exist in Co and Co_3_O_4_, respectively, it can be inferred that the introduction of CoB leads to more electron loss in Co and more electron gain in Co_3_O_4_. To clarify the electron transfer among different interfaces in Co/Co_3_O_4_/CoB and Co/Co_3_O_4_, DFT calculations were conducted. Herein, by balancing the computation power and calculation accuracy, the Co/Co_3_O_4_/CoB was modeled by Co (111) and Co_3_O_4_ (311) clusters supported on CoB (021), while Co/Co_3_O_4_ was modeled by Co (111) clusters supported on Co_3_O_4_ (311) (for details see DFT calculations in Method section). Firstly, the Bader charge was analyzed over Co/Co_3_O_4_ and Co/Co_3_O_4_/CoB models. For the Co/Co_3_O_4_ model, 3.64 |e| is transferred from Co to Co_3_O_4_ (Supplementary Fig. 6, Supplementary Table 1, and Supplementary Data 1). In Co/Co_3_O_4_/CoB, Co_3_O_4_ gets 2.23 |e| and 2.36 |e| from Co and CoB, respectively (Supplementary Fig. 7, Supplementary Table 1, and Supplementary Data 1). The charge density difference analysis of Co/Co_3_O_4_/CoB indicates a significant charge redistribution at the interface between CoB and Co/Co_3_O_4_ (Fig. 2c), with electron transfer from Co to CoB and from CoB to Co_3_O_4_. Furthermore, the average numbers of electron transfer per Co atom in Co/Co_3_O_4_/CoB and Co/Co_3_O_4_ were analyzed. As compared in Supplementary Table 1, Co loses more electrons (−0.22 e vs. −0.13 e) and Co_3_O_4_ gains more electrons (+0.51 e vs. +0.08 e) in Co/Co_3_O_4_/CoB as compared to Co/Co_3_O_4_, evidencing that CoB introduction can modulate the electronic structure of Co and Co_3_O_4_ in Co/Co_3_O_4_/CoB. X-ray absorption spectroscopy (XAS), including X-ray absorption near-edge structure (XANES) and extended X-ray absorption fine structure (EXAFS), was performed to examine the electronic structure and coordination environment of Co in Co/Co_3_O_4_/CoB and Co/Co_3_O_4_. In the Co K-edge XANES spectra (Fig. 2d), the position of the absorption edge for Co/Co_3_O_4_/CoB and Co/Co_3_O_4_ are located between that for Co foil and Co_3_O_4_ references, indicating that the average valence state of Co atom in Co/Co_3_O_4_/CoB and Co/Co_3_O_4_ are within 0 and +2/+3, owing to co-existence of both metallic Co and Co_3_O_4_. To understand the local structure around Co atom in these samples, the EXAFS spectra are transformed into R-space. The R-space EXAFS spectra (Fig. 2e) display dominant peaks of Co-B (1.60 Å)^45^, Co-Co (2.17 Å)^46^, and Co-O (1.44 Å)^47^ coordination bonds in CoB, Co foil and Co_3_O_4_, respectively. The FT-EXAFS spectra of Co/Co_3_O_4_/CoB and Co/Co_3_O_4_ can be well-fitted using backscattering paths of Co-Co, Co-B and Co-O (Fig. 2f, Supplementary Tables 2 and 3). Notably, the Co-O coordination number (2.2) in Co/Co_3_O_4_/CoB is significantly lower than that (5.7) in Co/Co_3_O_4_. The low-coordinated Co-O structure in Co/Co_3_O_4_/CoB is beneficial for NO adsorption^48^. The wavelet transform (WT) contour plot of Co/Co_3_O_4_/CoB (Fig. 2g) displays three intensity maximums at around 7.2, 5.9, and 5.4 Å^−1^, corresponding to the Co-Co, Co-B, and Co-O coordination, respectively^47,49^. For comparison, Co/Co_3_O_4_ only shows Co-Co and Co-O coordinations (Supplementary Fig. 8).

### Electrochemical NORR performance

**Fig. 3:**
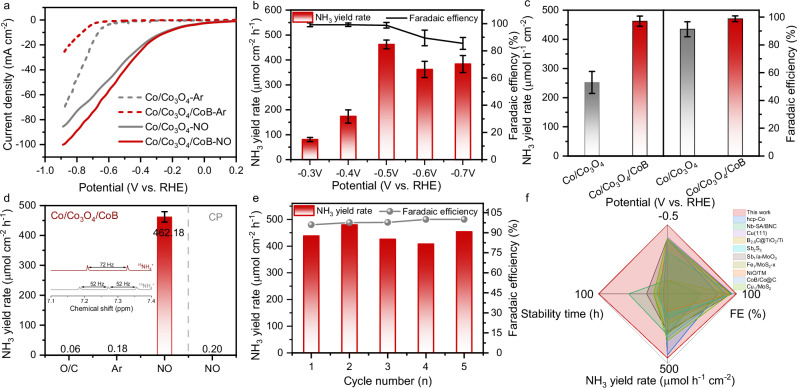
Electrochemical NORR performance. **a** LSV curves of Co/Co_3_O_4_ and Co/Co_3_O_4_/CoB with a scanning of 5 mV/s recorded in 0.1 M Ar-saturated and NO-saturated PBS (pH = 7.3 ± 0.1, with 90% iR-correction). **b** FE_NH3_ and NH_3_ yield rate over Co/Co_3_O_4_/CoB at various cathodic potentials with a mass loading of 0.2 mg cm^−2^ at 25 °C. **c** Comparison of NORR performance between Co/Co_3_O_4_ and Co/Co_3_O_4_/CoB at -0.5 V vs. RHE. **d** NORR performance over Co/Co_3_O_4_/CoB and carbon paper (CP) under different conditions (O/C represents open circuit condition). The inset shows the ^1^H nuclear magnetic resonance spectra to quantify NH_4_^+^. **e** Stability test of Co/Co_3_O_4_/CoB at −0.5 V vs. RHE. **f** Comparison of electrocatalytic NORR performance of Co/Co_3_O_4_/CoB with other reported electrocatalysts in literature. The error bars denote the standard deviation derived from three independent measurements, with the central marker indicating the mean value. Source data for Fig. 3 are provided as a Source Data file.

The electrochemical NO reduction performance over Co/Co_3_O_4_/CoB was assessed in an H-type electrolytic cell filled with 0.1 M phosphate buffer saline (PBS) aqueous solution. As shown in Fig. 3a and Supplementary Fig. 9, the linear sweep voltammetry (LSV) curves (iR-corrected or not) reveal that both Co/Co_3_O_4_/CoB and Co/Co_3_O_4_ exhibit a significantly increased current density in 10 vol.% NO/Ar atmosphere as compared to pure Ar atmosphere, suggesting preferred NORR on Co/Co_3_O_4_/CoB and Co/Co_3_O_4_ surface. Chronoamperometric (1 h electrolysis) and ultraviolet-visible (UV-vis) colorimetric measurements were used to quantitatively determine the NH_3_ Faradaic efficiency (FE) and yield rate (Supplementary Figs. 10 and 11). At −0.5 V vs. RHE, the Co/Co_3_O_4_/CoB displays the optimum FE_NH3_ of 98.80% and NH_3_ yield rate of 462.18 µmol h^−1^ cm^−2^ (2.31 mol h^−1^ g_cat_^−1^), much higher than Co/Co_3_O_4_ (FE_NH3_ of 91.28% and NH_3_ yield rate of 252.34 µmol h^−1^ cm^−2^, Fig. 3b, c). ^1^H nuclear magnetic resonance (NMR) measurements give comparable results (Supplementary Figs. 12–14). The possible by-products produced over Co/Co_3_O_4_/CoB during NORR were checked by differential electrochemical mass spectroscopy (DEMS), UV-vis absorption spectroscopy, and gas chromatography (Supplementary Figs. 15–18). The hydroxylamine (NH_2_OH), N_2_ and N_2_O by-products were not detected. Moreover, we conducted the NORR experiment over Co/Co_3_O_4_/CoB in 0.1 M KOH aqueous electrolyte. The NORR performance was poorer as compared with the experiment conducted in 0.1 M PBS (Supplementary Fig. 19). The better NORR performance of Co/Co_3_O_4_/CoB as compared to that of Co/Co_3_O_4_ can be further reflected by the smaller semicircle radius in the electrochemical impedance spectroscopy (EIS) spectrum (Supplementary Fig. 20), indicating a smaller charge transfer resistance (R_ct_) over Co/Co_3_O_4_/CoB surface. A series of control experiments indicate that the nitrogen in the NH_3_ product originates solely from NO (Fig. 3d). Carbon paper itself shows negligible NORR activity, disclosing that NH_3_ is mainly produced on Co/Co_3_O_4_/CoB (Fig. 3d). Using isotopic ^15^NO as the reactant, the characteristic double peaks (inset in Fig. 3d) appear with a separation of 72 Hz, corresponding to ^15^NH_4_^+^, indicating that ^15^NO is the sole nitrogen source of NH_3_ production. Besides excellent catalytic activity, Co/Co_3_O_4_/CoB also displays good catalytic stability. During five consecutive electrolysis cycles at −0.5 V vs. RHE, the FE_NH3_ and NH_3_ yield rate remain nearly unchanged (Fig. 3e). Moreover, this catalyst exhibits good current stability for ~100 h at −0.5 V vs. RHE (Supplementary Fig. 21). Co/Co_3_O_4_/CoB shows a good structural stability during the NORR based on the XRD, HRTEM and XPS characterizations (Supplementary Figs. 22–24). The ternary Co/Co_3_O_4_/CoB achieves a high FE_NH3_ of 98.80% and NH_3_ yield rate of 462.18 µmol h^−1^ cm^−2^ as well as long-time stability at a low cathodic potential of −0.5 V vs. RHE, superior to most of the reported NORR electrocatalysts (Fig. 3f and Supplementary Tables 4 and 5). To demonstrate the practical application potential of the Co/Co_3_O_4_/CoB catalyst, a Zn-NO battery with Co/Co_3_O_4_/CoB cathode and Zn plate anode was assembled (Supplementary Fig. 25), which could deliver an open circuit voltage (OCV) of 2.04 V (Supplementary Fig. 26) and a maximum power density of 10.06 mW cm^−2^, outperforming all reported results in the literature (Supplementary Figs. 27 and 28, Supplementary Table 6). The output discharge current density increases continuously from 0.5 to 8 mA cm^−2^ and each step exhibits a stable discharging plateau, indicating that our cell has an excellent discharge capability (Supplementary Fig. 29). Moreover, the NH_3_ yield rate exhibits a maximum of 1627.67 µg h^−1^ mg_cat_^−1^ at 8 mA cm^−2^ (Supplementary Fig. 30). Significantly, the Zn-NO battery can remove NO pollutant, produce NH_3_ and generate electricity at the same time.

### Reaction mechanism

**Fig. 4:**
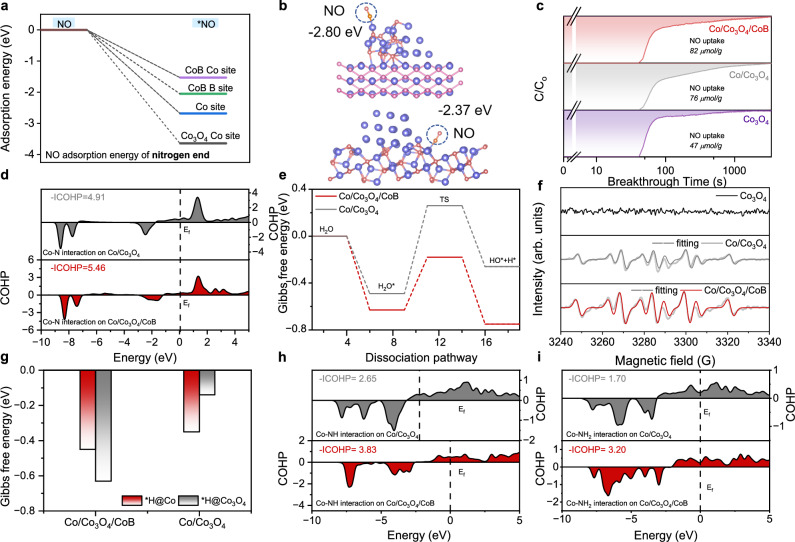
Reaction mechanism. **a** The calculated NO adsorption energy of nitrogen end on Co (111), CoB (021) and Co_3_O_4_ (311). **b** The calculated NO adsorption energy on Co/Co_3_O_4_/CoB and Co/Co_3_O_4_. The blue circle marks the NO molecule. Atom color-coding: Purple, cobalt; red, oxygen; pink, boron; nitrogen, orange. **c** NO adsorption breakthrough curves of Co/Co_3_O_4_/CoB, Co/Co_3_O_4_ and Co_3_O_4_ catalysts at 25 °C. **d** The COHP analysis of Co-N bond during NO adsorption on Co/Co_3_O_4_/CoB and Co/Co_3_O_4_. **e** Gibbs free energy for H_2_O dissociation on Co/Co_3_O_4_/CoB and Co/Co_3_O_4_. **f** EPR spectra recorded over Co/Co_3_O_4_/CoB, Co/Co_3_O_4_ and Co_3_O_4_ upon electrolysis in the absence of NO. **g** Comparison of Gibbs free energy for *H adsorption on Co and Co_3_O_4_ sites of Co/Co_3_O_4_/CoB and Co/Co_3_O_4_. The COHP analysis of **h** *NH and **i** *NH_2_ on Co/Co_3_O_4_/CoB and Co/Co_3_O_4_. Source data for Fig. 4 are provided as a Source Data file.

To understand the excellent NORR performance of Co/Co_3_O_4_/CoB, Brunauer-Emmett-Teller (BET) surface area and electrochemically active surface area (ECSA) of the as-prepared catalysts were first measured. Compared to Co/Co_3_O_4_, Co/Co_3_O_4_/CoB displays a higher BET surface area (Supplementary Fig. 31) and ECSA (Supplementary Fig. 32), which shall offer more active sites to catalyze electrochemical NORR. To clarify the reactive sites for NO adsorption/activation and H_2_O dissociation as well as the protonation process of adsorbed intermediates on Co/Co_3_O_4_/CoB, a series of DFT calculations, NO breakthrough and electron paramagnetic resonance (EPR) measurements were conducted. The NO adsorption energies with N-end/O-end on the Co sites of Co (111), Co_3_O_4_ (311), and CoB (021) and the B sites of CoB (021) were computed by DFT (Fig. 4a and Supplementary Figs. 33–35). The results indicate that NO prefers to adsorb on Co sites of Co_3_O_4_ (311) in the form of N-end adsorption, having the most negative adsorption energy of −3.64 eV. Compared to Co/Co_3_O_4_, Co/Co_3_O_4_/CoB binds to NO more strongly (Fig. 4b and Supplementary Data 1). The low-coordinated Co-O structure in Co/Co_3_O_4_/CoB (Fig. 2f and Supplementary Table 2) contributes to NO adsorption as demonstrated by NO breakthrough analysis (Fig. 4c)^47^. A crystal orbital Hamilton population (COHP) was further employed to investigate the NO adsorption strength on Co/Co_3_O_4_/CoB and Co/Co_3_O_4_ (Fig. 4d)^50–52^. Compared to Co/Co_3_O_4_, Co/Co_3_O_4_/CoB displays a more negative integrated COHP (ICOHP) value of the Co-N bond, indicating a stronger NO adsorption on the Co/Co_3_O_4_/CoB surface^53^. DFT calculation was further performed to explore the water dissociation process. The energy barrier for water transformation from the adsorbed state (H_2_O*) to the transition state (TS) on Co/Co_3_O_4_/CoB and Co/Co_3_O_4_ are 0.50 eV and 0.66 eV, respectively (Fig. 4e, Supplementary Figs. 36 and 37, Supplementary Table 6). This result indicates that the dissociation of H_2_O is more kinetically favorable on Co/Co_3_O_4_/CoB. In order to further investigate the H_2_O dissociation ability of Co/Co_3_O_4_/CoB and Co/Co_3_O_4_, in-situ Raman spectroscopy measurements were conducted at different applied cathodic potentials. Three types of O-H stretching modes belonging to 4-HB·H_2_O, 2-HB·H_2_O, and M·H_2_O (M = Na^+^/K^+^), respectively^54^, are observed. The relative proportion of M·H_2_O displays a little increasing trend along with increasing the applied cathodic potential, suggesting that M·H_2_O is closer to the catalyst’s surface and could be more affected by the electric field. The M·H_2_O proportion over Co/Co_3_O_4_/CoB is always higher than that over Co/Co_3_O_4_ at all studied cathodic potentials, suggesting better water dissociation ability over Co/Co_3_O_4_/CoB (Supplementary Figs. 38 and 39)^55,56^. Consequently, Co/Co_3_O_4_/CoB could generate more *H as reflected in the liquid EPR measurement using 5,5-dimethyl-1-pyrroline-N-oxude (DMPO) as the *H trapping regent (Fig. 4f)^57^. The DMPO-H signal completely disappeared when NO was introduced into the reaction system (Supplementary Fig. 40), indicating that the generated *H could be rapidly consumed by NO on the Co/Co_3_O_4_/CoB surface. This further verifies that *H is more favorable for bonding with NO to form NH_x_ intermediates rather than generating H_2_^58^. The *H generated on the metallic Co sites in Co/Co_3_O_4_/CoB can be quickly transferred to the neighboring Co_3_O_4_ surface to trigger the *NO hydrogenation reaction (Fig. 4g), thanks to the relatively electron-richer Co_3_O_4_ in Co/Co_3_O_4_/CoB than that in Co/Co_3_O_4_. As a result, the enhanced charge and *H transfer over Co/Co_3_O_4_/CoB promote electrochemical NO reduction to produce NH_3_. The COHP analysis was performed to investigate the adsorption capacity of nitrogen-containing intermediates on Co/Co_3_O_4_/CoB and Co/Co_3_O_4_. The ICOHP value of the Co-NH/Co-NH_2_ bond is −3.83/3.20 and −2.65/1.70 on Co/Co_3_O_4_/CoB and Co/Co_3_O_4_, respectively (Fig. 4h, i, Supplementary Figs. 41 and 42), indicating a higher bonding state energy of *NH/*NH_2_ on Co/Co_3_O_4_/CoB than Co/Co_3_O_4_. The projected density of states (PDOS) of Co/Co_3_O_4_/CoB and Co/Co_3_O_4_ upon adsorption of *NH and *NH_2_ were analyzed (Supplementary Figs. 43 and 44). The E_p_ (the highest peak below the Fermi level) position of Co/Co_3_O_4_/CoB is higher than that of Co/Co_3_O_4_ upon adsorption of *NH and *NH_2_, indicating that the antibonding states of NORR intermediates are located at higher energies with lower occupancies on Co/Co_3_O_4_/CoB surface, which results in stronger bindings of intermediates on Co/Co_3_O_4_/CoB during NORR^59^. Both the COHP and PDOS results indicate enhanced adsorption of *NH and *NH_2_ intermediates over Co/Co_3_O_4_/CoB, which is conducive to NORR for producing NH_3_.

**Fig. 5:**
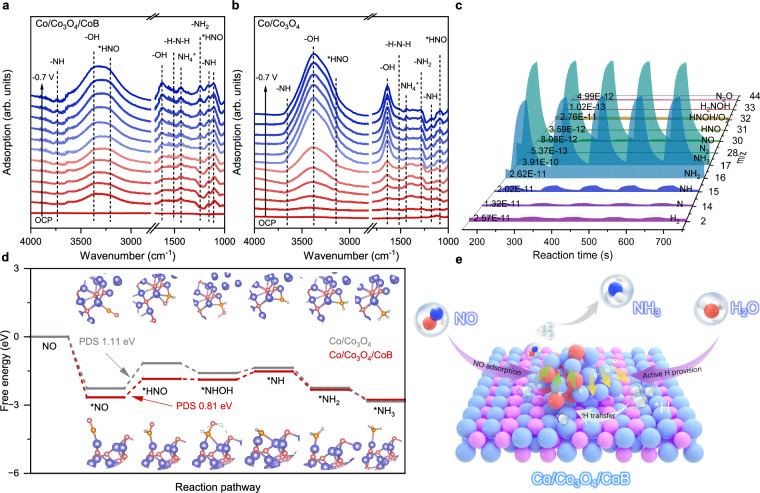
Reaction pathway. In-situ ATR-SEIRAS spectra recorded over Co/Co_3_O_4_/CoB **a** and Co/Co_3_O_4_ **b** in 0.1 M PBS at the applied cathodic potential from OCP to −0.7 V vs. RHE. **c** Online DEMS measurement results over Co/Co_3_O_4_/CoB at −0.5 V vs. RHE. **d** The calculated NORR Gibbs free energy diagrams over Co/Co_3_O_4_ and Co/Co_3_O_4_/CoB at 0 V vs. RHE. Atom color-coding: Purple, cobalt; red, oxygen; orange, nitrogen; gray, hydrogen. **e** Schematic illustration showing the NORR mechanism over Co/Co_3_O_4_/CoB electrocatalyst. Atom color-coding: Light blue, cobalt; pink, boron; red, oxygen; deep blue, nitrogen; gray, hydrogen. Source data for Fig. 5 are provided as a Source Data file.

To reveal the reaction pathway of electrochemical NORR, in-situ attenuated total reflection surface enhanced infrared absorption spectroscopy (ATR-SEIRAS) measurements were conducted to probe the reaction intermediates in the potential range from OCP to −0.7 V (vs. RHE). Over the Co/Co_3_O_4_/CoB surface (Fig. 5a), the bands at 1103 and 3224 cm^−1^ belong to *HNO species while the bands at 1167, 1245, and 1504 cm^−1^ are related to N-H stretching, -NH_2_ wagging, and -H-N-H bending, respectively^60,61^. The gradually increased bands at 1456 and 1635 cm^−1^ can be attributed to NH_4_^+^ and -OH species accumulation on the Co/Co_3_O_4_/CoB surface^62^. Additionally, a wide overlapped absorption at around 3000 ~ 3800 cm^−1^ is observed, in which the bands at 3380 and 3731 cm^−1^ are assigned to the stretching of -OH and -NH, respectively^63^. The in-situ ATR-SEIRAS spectra as a function of time also display the same reaction intermediates over Co/Co_3_O_4_/CoB surface (Supplementary Fig. 45). Compared to Co/Co_3_O_4_ (Fig. 5b), a large number of important reactive intermediates including *NH and *NH_2_ can be observed over Co/Co_3_O_4_/CoB surface at less negative cathodic potentials, suggesting much enhanced NORR reaction kinetics on Co/Co_3_O_4_/CoB. On the other hand, a larger amount of *NH and *NH_2_ intermediates were observed accumulating on Co_3_O_4_ surface compared with Co/Co_3_O_4_ and Co/Co_3_O_4_/CoB, implying the weaker ability of supplying *H on Co_3_O_4_ (Supplementary Fig. 46). To further confirm the key reaction intermediates, the DEMS measurement was performed over Co/Co_3_O_4_/CoB at −0.5 V vs. RHE. As displayed in Fig. 5c and Supplementary Fig. 47, it can be observed that NO decreases with a decrease of NOH and HNOH intermediates, and meanwhile NH, NH_2_, and NH_3_ increase. These intermediates observed in DEMS align well with the results obtained from in-situ ATR-SEIRAS measurements. Therefore, the NORR pathway taking place on the Co/Co_3_O_4_/CoB surface is speculated to follow *NO → *HNO→ *NHOH → *NH → *NH_2_ → *NH_3_ → NH_3_. Additionally, the signal of NH_3_ in the DEMS measurement is much stronger than that of H_2_, N_2_, NH_2_OH, and N_2_O, explaining the high FE_NH3_ in NORR over Co/Co_3_O_4_/CoB. Figure 5d compares the reaction energy diagram of NORR over Co/Co_3_O_4_/CoB and Co/Co_3_O_4_ surface (the structural models of intermediates are shown in Supplementary Figs. 48 and 49). Compared with *NOH, the first protonation of NO is more energetically favorable to form *HNO (Supplementary Figs. 50 and 51), which is the potential-determining step (PDS) of NORR on Co/Co_3_O_4_/CoB and Co/Co_3_O_4_. Clearly, Co/Co_3_O_4_/CoB shows a lower reaction energy barrier (0.81 eV) in PDS than that (1.11 eV) of Co/Co_3_O_4_, owing to the accelerated protonation process of NO over Co/Co_3_O_4_/CoB. The second proton preferentially reacts with *HNO to generate *HNOH with a ΔG of −1.88 eV, rather than *N with a ΔG of −0.82 eV. Finally, three successive protonation processes occur until NH_3_ is formed. Besides, the reaction energy of NORR was also calculated over Co_3_O_4_, Co, and CoB (Supplementary Figs. 52–55 and Table 7). The reaction energy of the potential-determining step (*NO to *HNO) is 1.25, 2.81 and 1.6 eV over Co_3_O_4_, Co, and CoB, respectively, much higher than that over Co/Co_3_O_4_/CoB. This result evidences that the first step of NO hydrogenation can be notably boosted over the ternary Co/Co_3_O_4_/CoB heterostructure.

## Discussion

In summary, we have designed and prepared a ternary Co/Co_3_O_4_/CoB heterostructure through a thermal solid-state reduction reaction of Co_3_O_4_ by NaBH_4_. The prepared Co/Co_3_O_4_/CoB displayed a high FE_NH3_ of 98.80% with NH_3_ yield rate of 462.18 µmol cm^−2^ h^−1^ (2.31 mol h^−1^ g_cat_^−1^) and long-time stability at a low cathodic potential of −0.5 V vs. RHE, outperforming most of the reported NORR electrocatalysts in the literature. The extraordinary NORR performance of Co/Co_3_O_4_/CoB was resulted from the unique charge and mass transfer within the Co/Co_3_O_4_/CoB heterostructure (Fig. 5e). The enhanced electron transfer in Co/Co_3_O_4_/CoB yielded electron-deficient Co and electron-rich Co_3_O_4_. The electron-deficient Co sites could effectively boost H_2_O dissociation to generate *H while the electron-rich low coordination Co_3_O_4_ sites promoted NO adsorption. The *H formed on electron-deficient Co site was more favorable to transfer to electron-rich Co_3_O_4_ site adsorbed with NO, facilitating the selective hydrogenation of NO. Thanks to the enhanced charge and proton transfer, the energy barrier of the potential-determining step from *NO to *HNO in NORR over Co/Co_3_O_4_/CoB was significantly reduced. Moreover, the introduction of CoB in Co/Co_3_O_4_/CoB could also facilitate the adsorption of *NH and *NH_2_ intermediates. All of which greatly promoted electrochemical NORR. This study paves the way for designing and developing highly efficient electrocatalysts for reducing NO to NH_3_.

## Methods

### Reagents and materials

Carbon paper (99.99%) and Ketjen carbon were purchased from Suzhou Sinero Technology Co., Ltd. Cobalt(II) acetate tetrahydrate (Co(CH_3_COO)_2_^.^4H_2_O, ≥99%), polyvinyl pyrrolidone (PVP, ≥99%), sodium borohydride (NaBH_4_, ≥98.0%), chloride (NH_4_Cl, 99.99%), ethanol (C_2_H_5_OH, ≥99.5%), were purchased from Shanghai Titan Scientific Co., Ltd. Hydroxylammonium chlorideammonium (NH_2_OH·HCl, ≥99%), dimethyl Sulfoxide-D6 (C_2_D_6_OS, 99.8%), sodium hydroxide (NaOH, ≥98%), trisodium citrate (C_6_H_5_Na_3_O_7_, 98%), sodium nitroprusside dihydrate (C_5_H_4_FeN_6_Na_2_O_3_·H_2_O, ≥99.98%), sulfuric acid (H_2_SO_4_, >95%), hydrogen peroxide (H_2_O_2_, 30%), and phosphate buffer solution (PBS, 10X, pH = 7.3 ± 0.1) were purchased from Shanghai Adamas Reagents Co., Ltd. Sodium hypochlorite solution (NaClO, available chlorine 4.0%), salicylic acid (C_7_H_6_O_3_, 99%) were purchased from Macklin Biochemical Technology Co., Ltd. Deionized water (18.25 MΩ cm resistivity) was obtained via an ultrapure water equipment in laboratory. All the reagents are analytical-grade and directly used without further purification.

### Synthesis of Co_3_O_4_ nanosheets

The Co_3_O_4_ nanosheets were prepared by a hydrothermal method as follows: Co(CH_3_COO)_2_.4H_2_O (0.75 g) and PVP (2.4 g) were dissolved in 70 mL methanol under stirring for 30 min. The mixture was transferred into a 100 mL Teflon-lined stainless-steel autoclave that was sealed and heated to 190 °C for 12 h. After cooling to 25 °C, the products were harvested by centrifugation, washed three times with ethanol, dried at 60 °C, and calcined at 350 °C for 5 h in air^64^.

### Synthesis of Co/Co_3_O_4_/CoB and Co/Co_3_O_4_

In a typical synthesis of Co/Co_3_O_4_/CoB, Co_3_O_4_ and NaBH_4_ were uniformly mixed in a mortar at a mass ratio of 1:1.5. The mixture was then placed in a horizontal quartz tube and heated to 500 °C at a ramp rate of 2 °C min^−1^ under an Ar atmosphere, which was maintained at 500 °C for 4 h. The resulting products were washed extensively by an ethanol/deionized water mixture and collected by filtration. Finally, the washed Co/Co_3_O_4_/CoB was dried at 80 °C for 12 h in a vacuum oven. For preparing Co/Co_3_O_4_, the mass ratio of Co_3_O_4_ and NaBH_4_ was adjusted to 1.5:1, while other preparation parameters were kept unchanged.

### Electrochemical measurements

Electrochemical measurements were performed on a CHI660E electrochemical workstation in an H-type electrochemical cell (Supplementary Fig. 56) separated by a Nafion 117 membrane (10*10 cm, thickness was 183 µm, Dupont) in a three-electrode configuration at 25 °C. We chose 0.1 M PBS buffer solution (pH = 7.3 ± 0.1) to maintain the pH of the electrolyte during NORR. The membrane was sequentially treated in a H_2_O_2_ (5 wt.%) aqueous solution at 80 °C for 1 h, then in a 0.5 M H_2_SO_4_ solution at 80 °C for 2 h and finally in deionized water at 25 °C for 6 h. The catalyst coated on carbon paper (CP,1 cm^2^) was used as the working electrode, a platinum plate (1 cm^2^) was used as the counter electrode, and an Ag/AgCl (saturated 3.5 M KCl aqueous solution) electrode was used as the reference electrode. The Ag/AgCl reference electrode was calibrated in H_2_-saturated 0.1 M PBS using a symmetric Pt electrode system.

To prepare the working electrode, 2.5 mg of electrocatalyst and 2.5 mg of Ketjen carbon were ultrasonically dispersed in 300 μL ethanol, 150 μL ultrapure water, and 25 μL Nafion solution (5 wt.%, Du Pont) to form a homogeneous catalyst ink followed by dropping 38 µL of the catalyst ink onto a piece of CP (1 cm^2^) that was dried at 25 °C, the catalyst mass loading was 0.2 mg cm^−2^. Before all electrochemical tests, 30 min of high-purity Ar gas (99.999%) and 30 min of NO/Ar gas (10 vol.%) saturated the electrolyte to exclude air in the reaction system.

Linear sweep voltammetry (LSV) was performed at a scanning rate of 5 mV/s prior to 50 cycles of cyclic voltammetry at a scan rate of 50 mV/s to obtain a stable curve with 90% iR-correction. The non-iR corrected data for all catalysts are provided in the Supplementary Information. The electrochemical impedance spectroscopy (EIS) was obtained without iR-correction in the frequency range from 0.01 Hz to 100 kHz upon an AC voltage amplitude of 5 mV at an open-circuit potential under 25 °C. The chronoamperometry was operated to evaluate the stability under continuous stirring (1600 rpm) at different current densities. All potentials in this study were converted to the reversible hydrogen electrode (RHE) scale according to the following equation:

1 $${{{\rm{E}}}}({{{\rm{V}}}}\,{{{\rm{vs}}}}.{{{\rm{RHE}}}})={{{\rm{E}}}}({{{\rm{V}}}}\, {{{\rm{vs}}}}.{{{\rm{Ag}}}}/{{{\rm{Ag}}}}{{{\rm{Cl}}}})+0.198\, {{{\rm{V}}}}+0.059\times {{{\rm{pH}}}}$$

Calculation of Faradaic efficiency (FE) and yield rate (Y)

2 $${{{{\rm{FE}}}}}_{i}\left( \% \right)=\frac{{{{{\rm{Q}}}}}_{i}}{{{{{\rm{Q}}}}}_{{{{\rm{total}}}}}}\times 100 \%=\frac{{{{{\rm{c}}}}}_{i}\times {{{\rm{V}}}}\times {{{\rm{n}}}}\times {{{\rm{F}}}}}{{M}_{i}\times {{{{\rm{Q}}}}}_{{{{\rm{total}}}}}}\times 100 \% $$

3 $${{{{\rm{Y}}}}}_{i}({{{\rm{\mu mol}}}}\, {h}^{-1}\, {{cm}}^{-2})=\frac{{{{\rm{V}}}}{\times {{{\rm{c}}}}}_{i}}{{M}_{i}\times {{{\rm{t}}}}\times {{{\rm{s}}}}}$$

Where Q_*i*_ is the charge of product *i*, Q_total_ is the total charge, c_*i*_ is the concentration of products (µg mL^−1^), V is the volume of electrolyte in the cathode compartment (25 mL), n: the number of electron transfer in products; F: Faraday constant (96485 C mol^−1^); M_*i*_: the molar mass of products (g mol^−1^); s: the area of the electrode (1 cm^2^); t: the reaction time (1 h).

### ECSA analysis

Electrochemical active surface area (ECSA) was evaluated through cyclic voltammetry (CV) measurements conducted within the non-Faradaic potential region in 0.1 M PBS electrolyte, where iR drop is negligible. The electrochemical double-layer capacitance (C_dl_) was determined from the linear slope of capacitive current (Δj = 0.5 × |j_charge_ − j_discharge_|) plotted against scan rate (20–120 mV s^−1^). The ECSA of the catalyst was determined by normalizing the double-layer capacitance (C_dl_) against the standard specific capacitance (C_s_) for smooth metal surfaces in 1.0 M netural electrolyte, following the relation: ECSA = C_dl_/C_s_.

### Quantification of NH_3_

The yield rate of NH_3_ in NORR was quantitatively determined by the indophenol blue method and NMR. For the indophenol blue method, the concentration-absorbance curves were calibrated using a standard NH_3_ solution with a series of concentrations. The fitting curve (y = 0.4149x – 0.0264, R^2^ = 0.9999) shows a good linear relationship between absorbance and NH_3_ concentration. For NMR measurement, after NORR, the electrolyte was taken out, whose pH was adjusted to 3 using a 0.5 M HCl aqueous solution, and then sent for quantification by ^1^H NMR (600 MHz) with internal standard of maleic acid (3.5 M). The number of scans was 128 for all NMR measurements. The fitting curve (y = 2.7375x – 1.9166, R^2^ = 0.9993) shows a good linear relationship.

### ^15^N isotope labeling experiment

The ^15^N isotope labeling experiment proceeded on the ^1^H NMR spectroscopy (600 MHz) to identify the nitrogen source for NORR. Replace ^14^NO with ^15^NO to conduct the NORR experiment at −0.5 V vs. RHE. After the experiment, take 2 mL of the catholyte were acidized by H_2_SO_4_ (2 mL, 1 M). 60 μL of the above solutions were respectively added to a NMR tube and mixed with maleic acid aqueous solution (20 μL, 3.6 mM), H_2_SO_4_ aqueous solution (20 μL, 4 M) and DMSO-d_6_ (500 μL), and then analyzed by ^1^H NMR measurements.

### Quantification of hydroxylamine (NH_2_OH)

To quantify NH_2_OH, the following literature procedures were executed: 1.0 mL of the catholyte (or 0.1 mM~0.5 mM standard NH_2_OH solution) was added 1.0 mL PBS buffer (pH = 7.3 ± 0.1) and 1.0 mL 1% 8-hydroxylquinoline. Under vigorous shaking, 1.0 mL 0.1 M Na_2_CO_3_ was added and the mixture was heated at 100 °C for 1 min. In the presence of NH_2_OH, the solution would turn from light yellow to blue-green, showing an absorption peak at ~705 nm. Plotting the peak absorbance with NH_2_OH concentration yielded a calibration curve.

### Zn-NO battery

A Co/Co_3_O_4_/CoB coated on carbon paper was employed as the cathode to perform the NORR in a cathodic electrolyte (0.1 M PBS). A polished Zn plate was applied as the anode in an anodic electrolyte (1 M KOH), and a Nafion 117 membrane was used to separate the cathode from the anode. The Zn-NO battery was assessed on a CHI660E electrochemical workstation under an ambient atmosphere at 25 °C.

### Characterizations

The morphological information was characterized by atomic force microscopy (AFM, Park NX10), field emission scanning electron microscopy (SEM, Zeiss-sigma-300), and transmission electron microscopy (TEM, JEOL JEM-1400). High-resolution transmission electron microscopy (HRTEM) and aberration-corrected high-angle annular dark-field scanning transmission electron microscopy (HAADF-STEM) were conducted on a JEOL JEM 2100 F and JEM-ARM 300 F Grand ARM. X-ray diffraction (XRD) patterns were recorded on a Bruker D8 Advance Diffractometer (Cu-Kα radiation: λ = 0.15406 nm). The surface valence states were studied by X-ray photoelectron spectroscopy (XPS, PHI-5300) with Mg Kα radiation (hν = 1486.6 eV). The X-ray absorption near edge structure (XANES) and extended X-ray absorption fine structure (EXAFS) spectra at the Co K-edge were collected at the HXMA beamline of the Canadian Light Source (CLS) using Fluorescence mode and Si (111) monochromator. The samples were pressed into wafers for collecting the data at 25 °C. The EXAFS raw data were background subtracted, normalized and Fourier transformed by standard procedures using the ATHENA program. The acquired EXAFS data were processed according to the standard procedures using the ATHENA module implemented in the IFEFFIT software packages. The k^3^-weighted EXAFS spectra were obtained by subtracting the post-edge background from the overall absorption and then normalizing for the edge-jump step. The ultraviolet-visible (UV‒vis) absorbance spectra were measured on an Agilent S3 Cary 5000. A Bruker 600 M NMR instrument with water suppression was used to record the ^1^H NMR spectra. The Brunauer-Emmett-Teller (BET) surface area was measured on a U.S. Quantachrome ASAP 2020 M at 77 K.

### NO adsorption breakthrough measurements

The NO adsorption breakthrough measurements were carried out on a dynamic sorption analyzer (mixSorb S) equipped with a thermal conductivity detector (TCD) combined with a mass spectrometer (OMNISTARTM). Firstly, 40 mg of catalyst was pre-treated in a high purity He atmosphere at a flow rate of 30 mL/min for 1 h. Afterwards, the sample was exposed to NO gas (0.02 vol.% NO balanced by He) at a total gas flow rate of 20 mL/min at 25 °C accompanied by recording the TCD signal and the MS signal of NO at m/z = 30 until the outlet mass signal of NO achieved saturation.

### In-situ attenuated total reflection-surface enhanced infrared absorption spectroscopy (ATR-SEIRAS) measurements

In-situ ATR-SEIRAS spectra were recorded on an INVENIO-R FTIR spectrometer (Bruker) equipped with a liquid nitrogen-cooled mercury cadmium telluride (MCT) detector (Supplementary Fig. 57). The catalyst ink was prepared by mixing 5 mg of catalyst, 300 μL ethanol, 150 μL ultrapure water, and 25 μL Nafion solution under sonication for 30 min. Next, 40 μL catalyst ink was slowly dropped onto a face-angled Si crystal to prepare the working electrode of the custom-made spectroelectrochemical cell fixed on the ATR accessory. Ag/AgCl electrode was employed as the reference electrode and a Pt wire was used as the counter electrode. Electrolyte was added in advance with continuous Ar flow at a flow rate of 50 mL/min for 30 minutes to remove interference of H_2_O and O_2_ prior to ATR-SEIRAS measurement. The background was measured at open circuit potential. Subsequently, 10% NO/Ar gas was injected and the absorption spectra were recorded at different applied potentials from OCP to −0.7 V vs. RHE. Afterwards, spectrum was collected at −0.5 V vs. RHE every 5 min for 60 min.

### Online differential electrochemical mass spectroscopy (DEMS) measurements

In-situ DEMS measurement was conducted on Linglu (Supplementary Fig. 58). A Teflon layer was applied to separate the electrolyte from the vacuum system. The vacuum system, comprising of two dry pumps and one turbo pump, kept the vacuum degree below 10^−7 ^Pa. The catalyst ink was prepared by mixing 3 mg of catalyst, 460 μL ethanol, 500 μL ultrapure water, and 40 μL Nafion solution under sonication for 30 min. Next, 40 μL catalyst ink was slowly dropped onto gold film to prepare the working electrode. Ag/AgCl electrode was employed as the reference electrode and a Pt wire was used as the counter electrode. Before the DEMS measurement, NO/Ar was bubbled into the electrolyte until saturation. I-*t* test for 100 s was used to conduct DEMS investigations.

### In-situ Raman spectroscopy

The in-situ Raman measurements were carried out jointly by an RXN1 Raman instrument (KAISER OPTICAL SYSTEM) and a CHI660E electrochemical workstation. A custom-made spectroelectrochemical cell was used as a reactor to enable the in-situ measurements. The obtained Co/Co₃O₄/CoB catalyst, Ag/AgCl, and platinum wire served as the working electrode, reference electrode, and counter electrode, respectively. The working electrode was immersed in the electrolyte and positioned such that the electrode plane was perpendicular to the laser. In-situ Raman spectra were obtained while the electrodes were under potentiostatic control. The experiment was conducted for 200 s under each fixed potential.

### EPR measurements

5,5-dimethyl-1-pyrroline N-oxide (DMPO) was used to capture the unstable hydrogen radical to form the DMPO-H adduct to generate EPR spectrum. Briefly, 5 mL of electrolyte was mixed with 100 μL of DMPO and the mixture was deoxygenated by bubbling Ar. The constant current electrolysis was carried out for 10 min in the H-type cell under the protection of Ar. EPR measurement was performed on a JES X320, JEOL Co. spectrometer operated at a frequency near 9.5 GHz, sweep width of 200 G, and power of 20 mW. Co/Co_3_O_4_/CoB fitting parameters: g = 2.0045, AN = 15.5 G, AH = 21.0 G, lwpp = 0.3. Co/Co_3_O_4_ fitting parameters: g = 2.0043, AN = 15.6 G, AH = 20.8 G, lwpp = 0.3.

### Density functional theory (DFT) calculations

Considering the limitations of computational power, we opted for a simplified mode, namely CoB supported neighboring Co and Co_3_O_4_ clusters, combininng with the morphology and three interfaces of Co/Co_3_O_4_/CoB heterostructure. The choice of using crystal facets of Co (111), Co_3_O_4_ (311), and CoB (021) to model Co/Co_3_O_4_/CoB heterostructure is primarily based on the XRD characterizations. These crystal facets exhibit the strongest diffraction in the XRD patterns and are also the thermodynamically stable facets. It is noted that the Co (111) and Co_3_O_4_ (311) clusters are taken from (111) facet of Co single crystal and (311) facet of Co_3_O_4_ single crystal, respectively.

All calculations were performed in the framework of the density functional theory with the projector augmented plane-wave method, as implemented in the Vienna ab initio simulation package (VASP)^65^. The generalized gradient approximation proposed by Perdew, Burke, and Ernzerhof was selected for the exchange-correlation potential^66^. The Grimme D3 correction used a coordination number dependent dispersion correction^67^. The cut-off energy for the plane wave was set to 450 eV. The energy criterion was set to 10^−5^ eV in the iterative solution of the Kohn-Sham equation. A vacuum layer of 15 Å was added perpendicular to the sheet to avoid artificial interaction between periodic images. The Brillouin zone integration was performed using a 2 × 2 × 1 k-mesh^68^. All the structures were relaxed until the residual forces on the atoms had declined to less than 0.05 eV/Å. The maximum atomic force in Co/Co_3_O_4_/CoB is 0.047, which meets the convergence requirement. In addition, we also evaluated the structural stability by using ab initio molecular dynamics (AIMD). The Brillouin zone was sampled using 1 × 1 × 1 k-point grid. Self-consistent calculations were conducted with an energy convergence threshold of 10^−5 ^eV. The AIMD was performed within the canonical (NVT) ensemble by Nosé-Hoover thermostats with a time step of 1.0 fs at a finite temperature of 300 K^69^. In the AIMD simulation up to 10 ps, the heterostructure of Co/Co_3_O_4_/CoB was not destroyed, and the basic crystal structure was stable, proving the rationality of the structure (Supplementary Figs. 59–61 and Supplementary Data 2). Spin polarization was included in the calculations and the default setting of magnetic moment (MAGMOM= number of atoms of Co*1.0) was chosen for all calculations. The Gibbs free energy (ΔG) of reaction intermediates was calculated by the following:

4 $$\Delta {{{\rm{G}}}}=\Delta {{{\rm{E}}}}+{\Delta {{{\rm{E}}}}}_{{{{\rm{ZPE}}}}}-{{{\rm{T}}}}\Delta {{{\rm{S}}}}$$

where ΔE is the adsorption energy. ΔE_ZPE_ and ΔS are the difference for the zero-point energy and entropy, respectively. The zero-point energy and entropy were calculated at the standard conditions corresponding to the pressure of 101325 Pa ( ~ 1 bar) of H_2_ at the temperature of 298.15 K. The climbing image nudged elastic band (cNEB) method was used to search the reaction path and transition state, and the vibration frequency calculation was used to confirm it further^70^.

## Supplementary information

Supplementary Information

Description Of Additional Supplementary File

Supplementary Data 1

Supplementary Data 2

Transparent Peer Review file

## Source data

Source data

## Data Availability

Source data for all the figures and tables generated in this study are provided as a Source Data file. Source data are provided with this paper.
