# Supplementary material for: Boosted charge and proton transfer over ternary Co/Co3O4/CoB for electrochemical nitric oxide reduction to ammonia
Source: Nat Commun. 2025 May 26;16:4874. doi: 10.1038/s41467-025-60043-6 (PMC12106605; doi:10.1038/s41467-025-60043-6)
Supplement: Supplementary file 1 — Supplementary Information [file 41467_2025_60043_MOESM1_ESM.pdf]

Supplementary Information for

Boosted charge and proton transfer over ternary $Co/Co_3O_4/CoB$ for electrochemical nitric oxide reduction to ammonia

Xiaoxuan Fan[1], Zhenyuan Teng[2], Lupeng Han[1,*], Yongjie Shen[3], Xiyang Wang[4], Wenqiang Qu[1], Jialing Song[1], Zhenlin Wang[1], Haiyan Duan[1], Yimin A. Wu[4], Bin Liu[2,5,*] and Dengsong Zhang[1,*]

[1]Innovation Institute of Carbon Neutrality, International Joint Laboratory of Catalytic Chemistry, State Key Laboratory of Advanced Special Steel, Department of Chemistry, College of Sciences, Shanghai University, Shanghai 200444, China.

[2]Department of Materials Science and Engineering, City University of Hong Kong, Hong Kong SAR 999077, China.

[3]Institute for Chemical Reaction Design and Discovery (WPI-ICReDD), Hokkaido University, Sapporo 001-0021, Japan.

[4]Department of Mechanical and Mechatronics Engineering, Waterloo Institute for Nanotechnology, University of Waterloo, Waterloo, Ontario, N2L 3G1, Canada.

[5]Department of Chemistry, Hong Kong Institute of Clean Energy (HKICE) & Center of Super-Diamond and Advanced Films (COSDAF), City University of Hong Kong, Hong Kong SAR 999077, China.

[*]Corresponding author. Email: lphan@shu.edu.cn (L. Han), bliu48@cityu.edu.hk (B. Liu) and dszhang@shu.edu.cn (D. Zhang)

Supplementary Figures

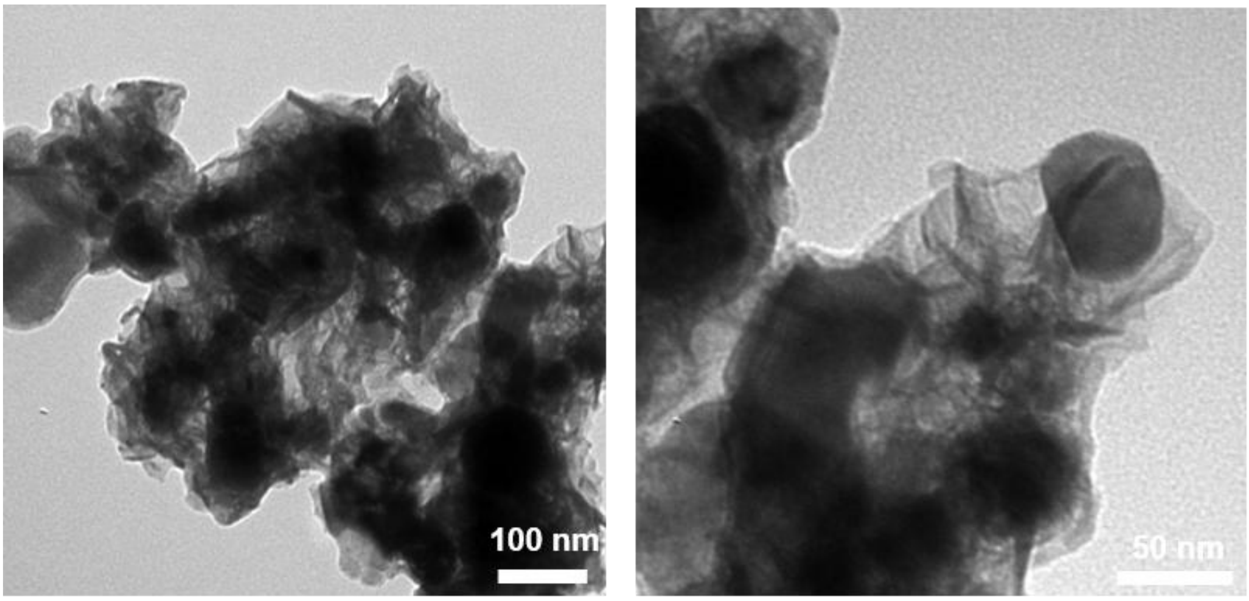

Supplementary Figure 1. TEM images of $Co/Co_3O_4/CoB$.

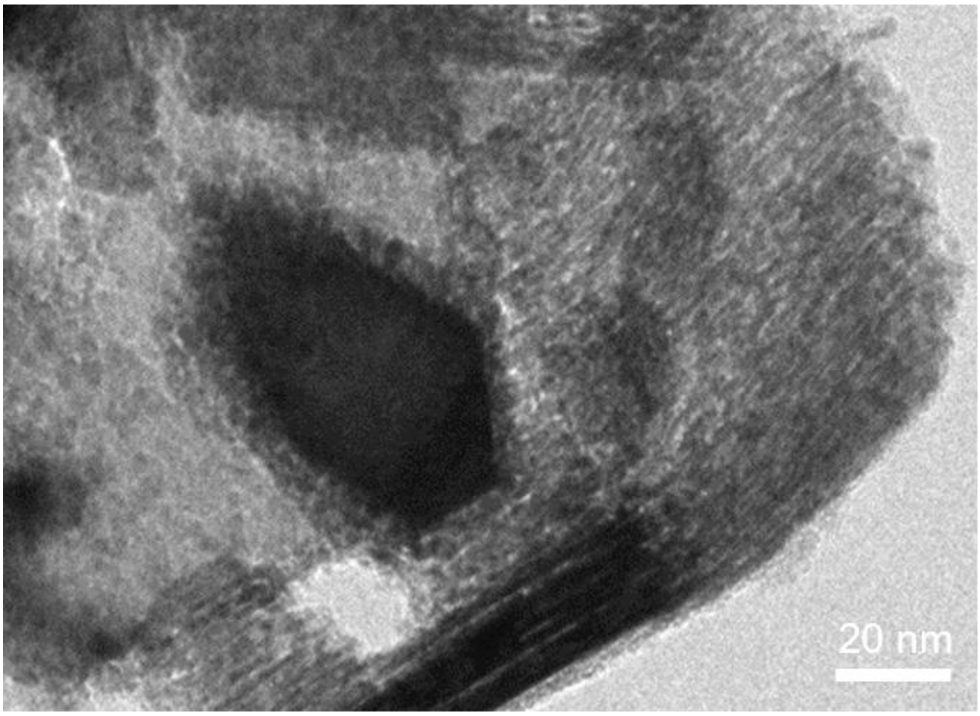

Supplementary Figure 2. The HRTEM image of $Co/Co_3O_4/CoB$.

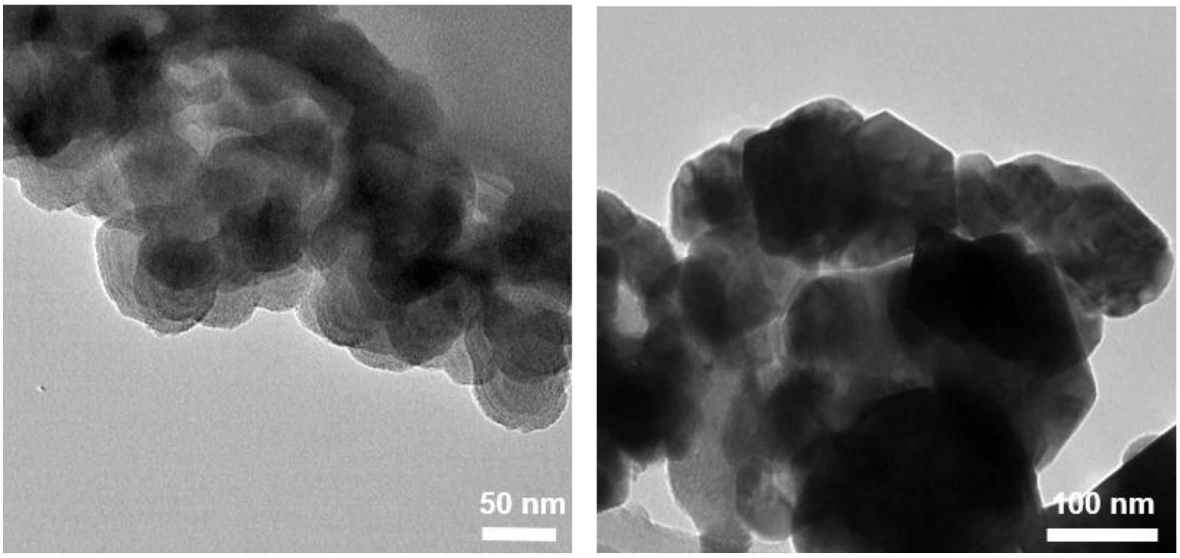

Supplementary Figure 3. TEM images of Co/Co_3O_4.

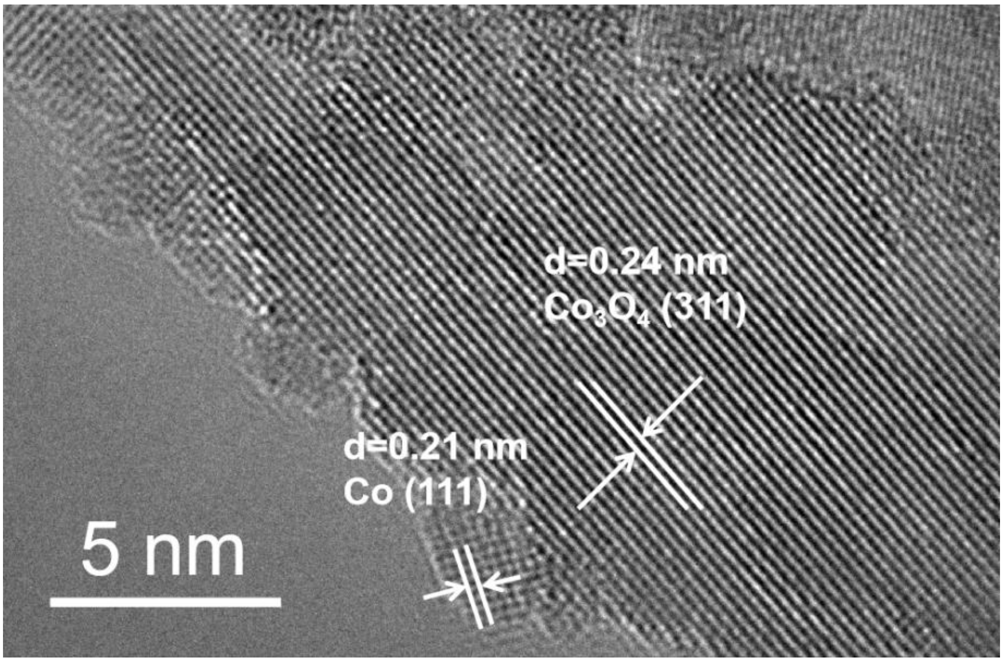

Supplementary Figure 4. The HRTEM image of Co/Co_3O_4.

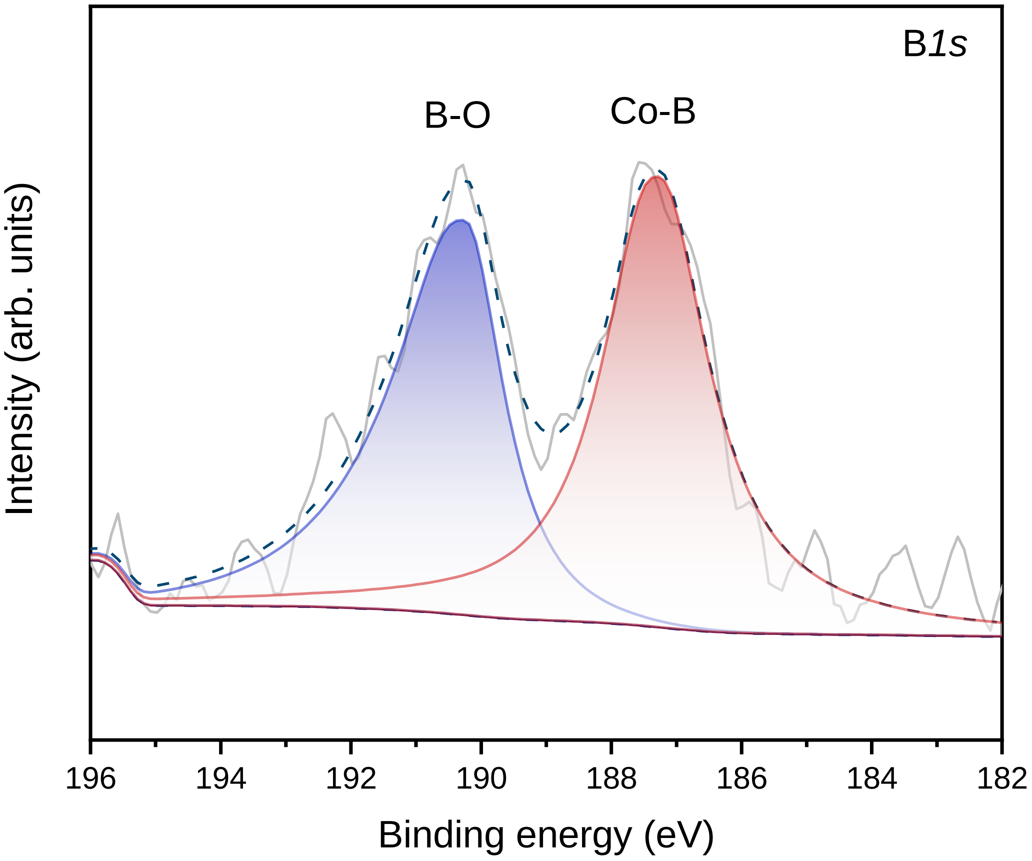

Supplementary Figure 5. B *1s* XPS spectrum for $Co/Co_3O_4/CoB$.

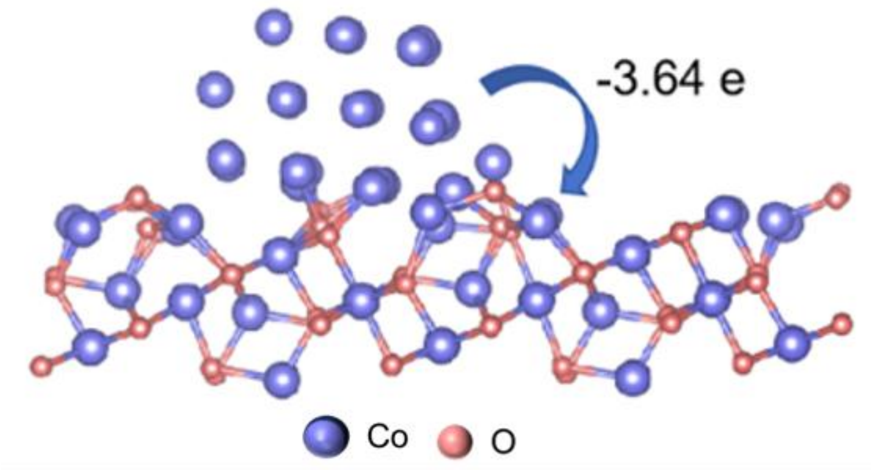

Supplementary Figure 6. The Bader charge analysis for Co/Co_3O_4.

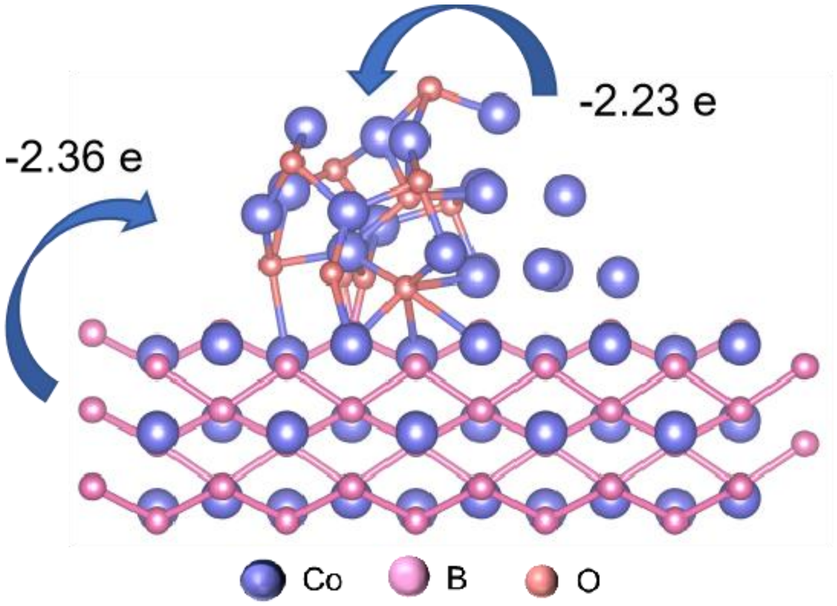

Supplementary Figure 7. The Bader charge analysis for $Co/Co_3O_4/CoB$.

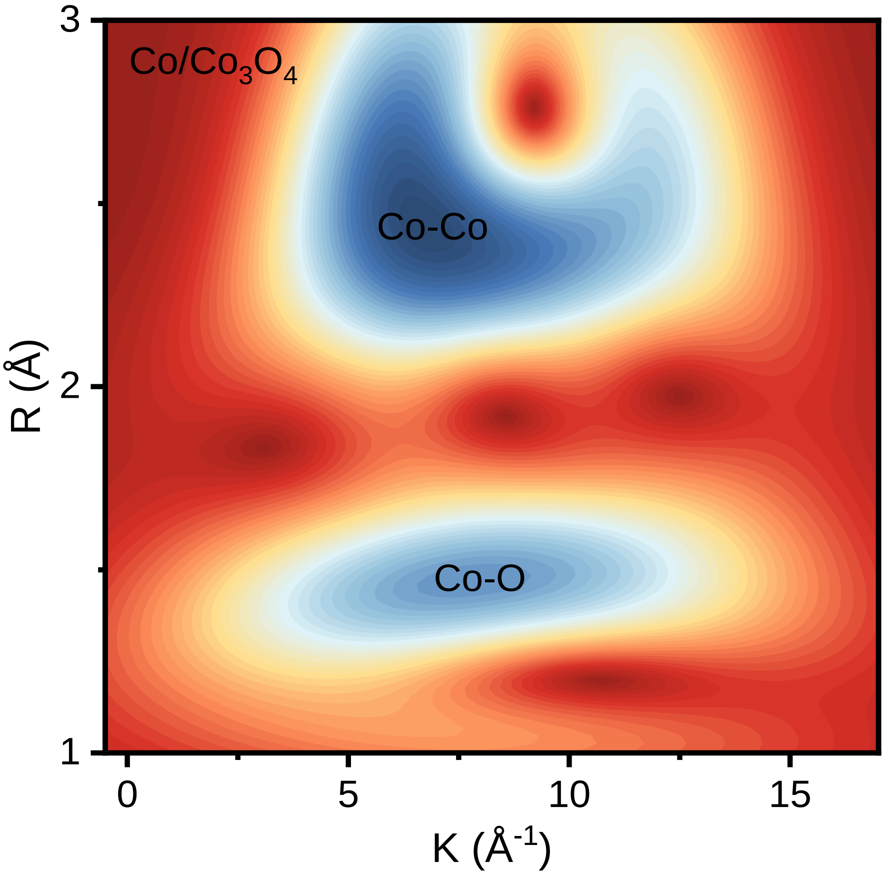

Supplementary Figure 8. Wavelet transform (WT) contour plot for Co/Co_3O_4.

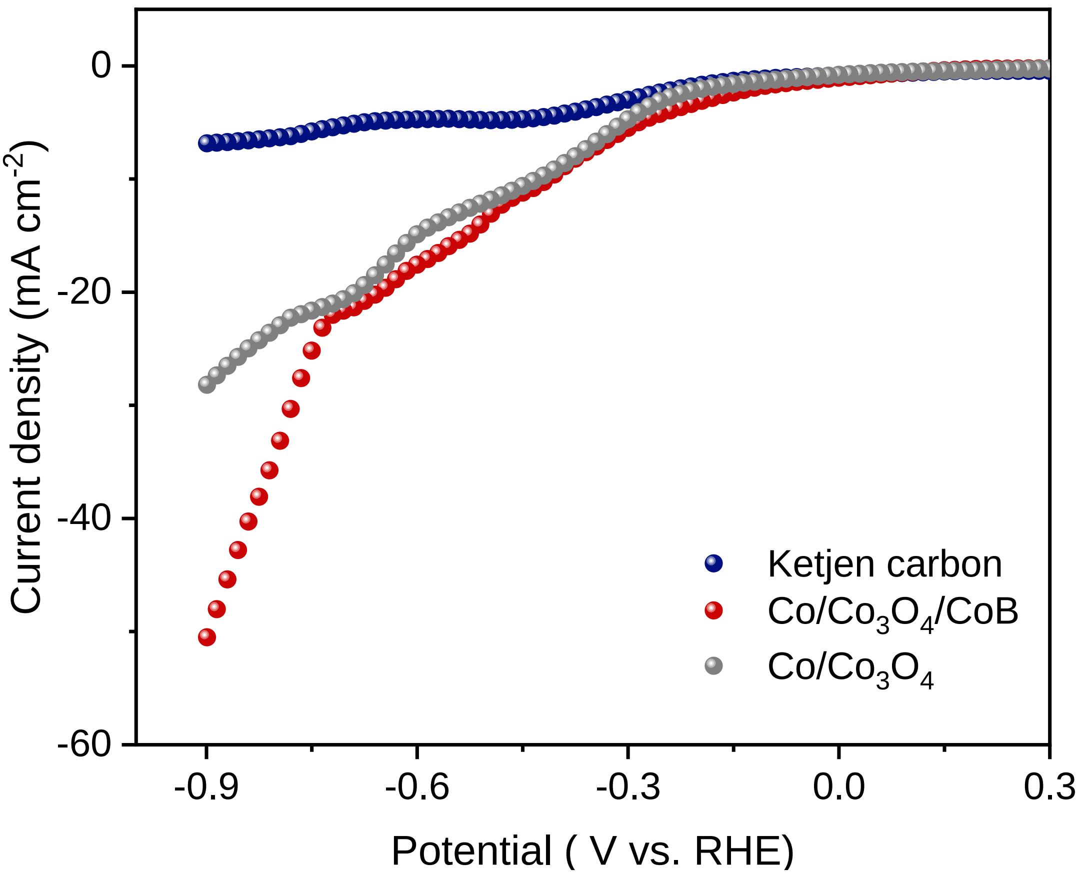

Supplementary Figure 9. The LSV curves without iR-compensation for all catalysts in 0.1 M NO-saturated PBS (electrode surface area: 1cm^2; resistance refers to the EIS section, Supplementary Figure 20).

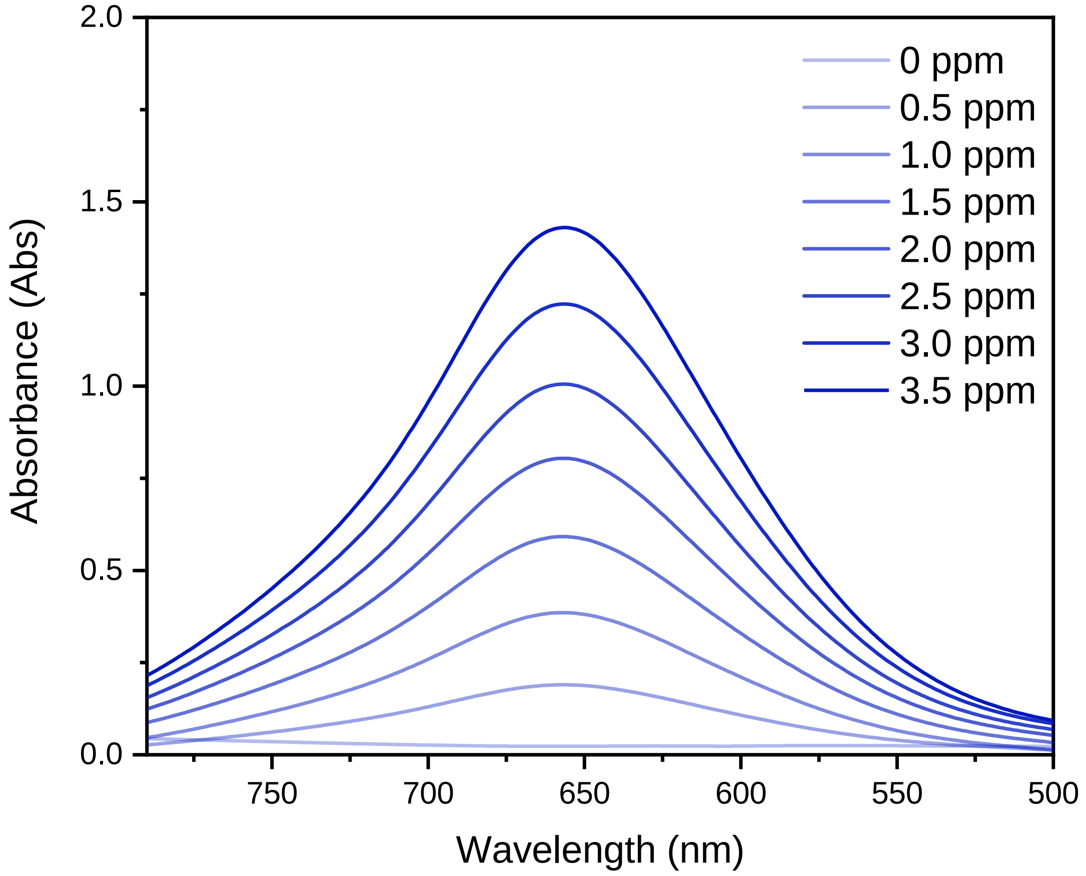

Supplementary Figure 10. UV-vis absorption spectra of NH_4^+ with concentration in the range of 0-3.5 ppm (mg/L) in 0.1 M PBS.

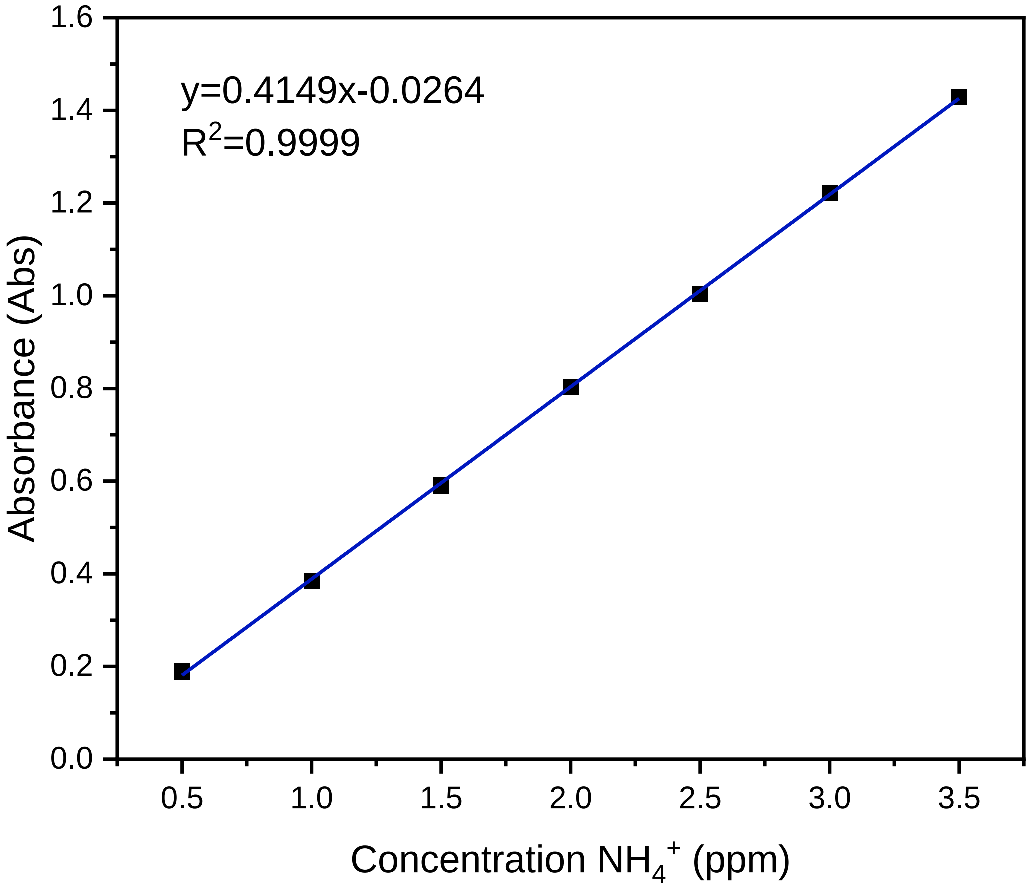

Supplementary Figure 11. The linear calibration curve of UV-vis for qantifying NH_4^+.

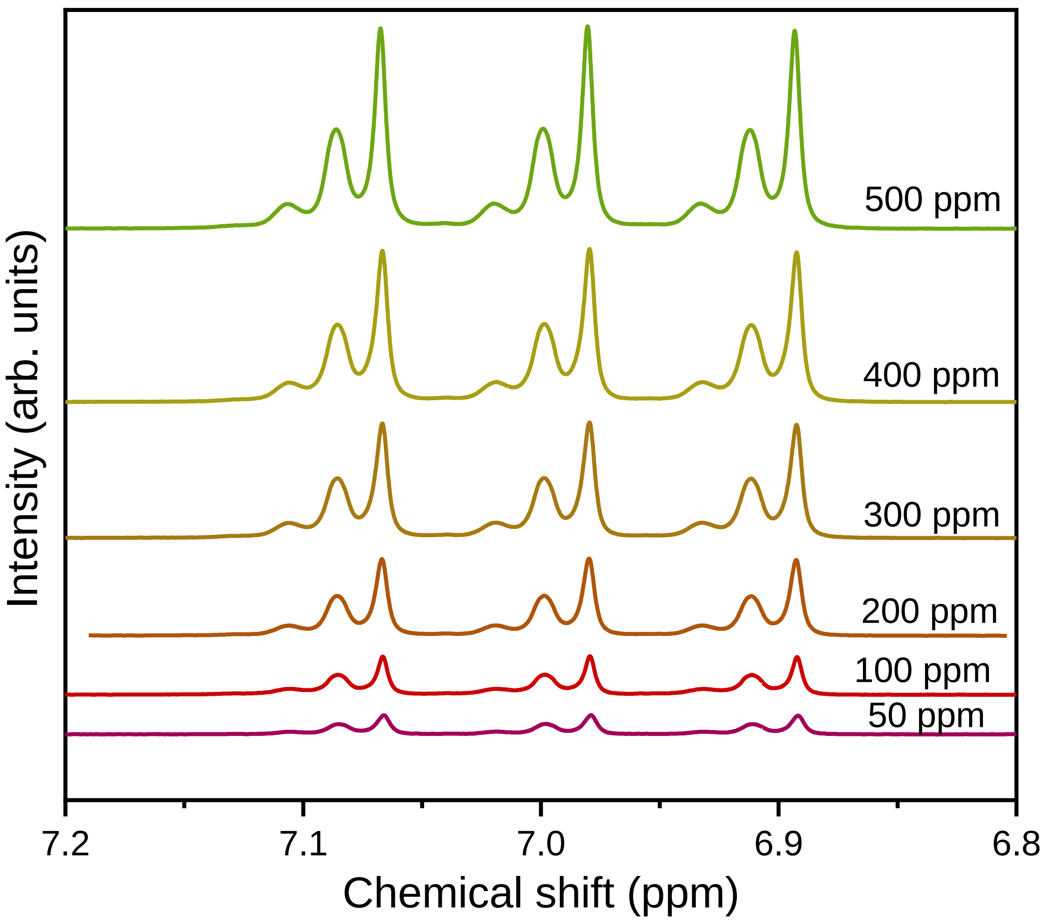

Supplementary Figure 12. ^{1}H NMR spectra of NH_4^+ with different concentrations.

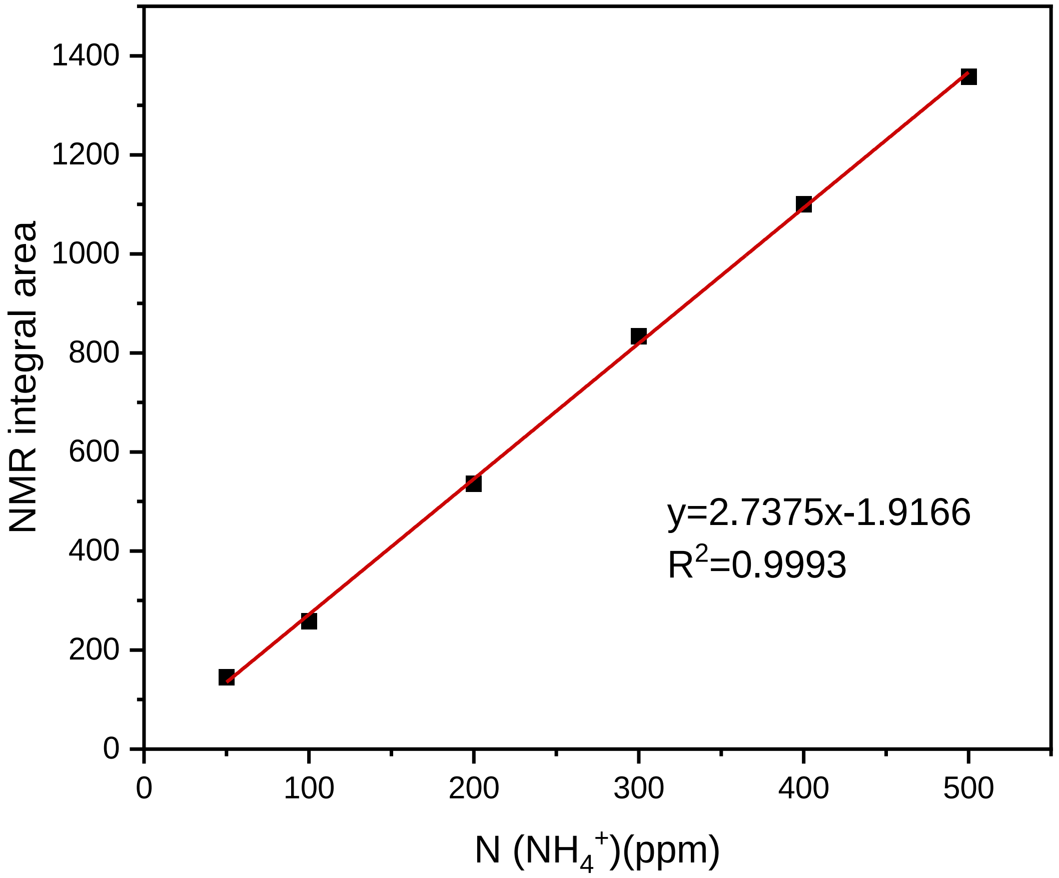

Supplementary Figure 13. The linear calibration curve of ^{1}H NMR for quantifying NH_4^+.

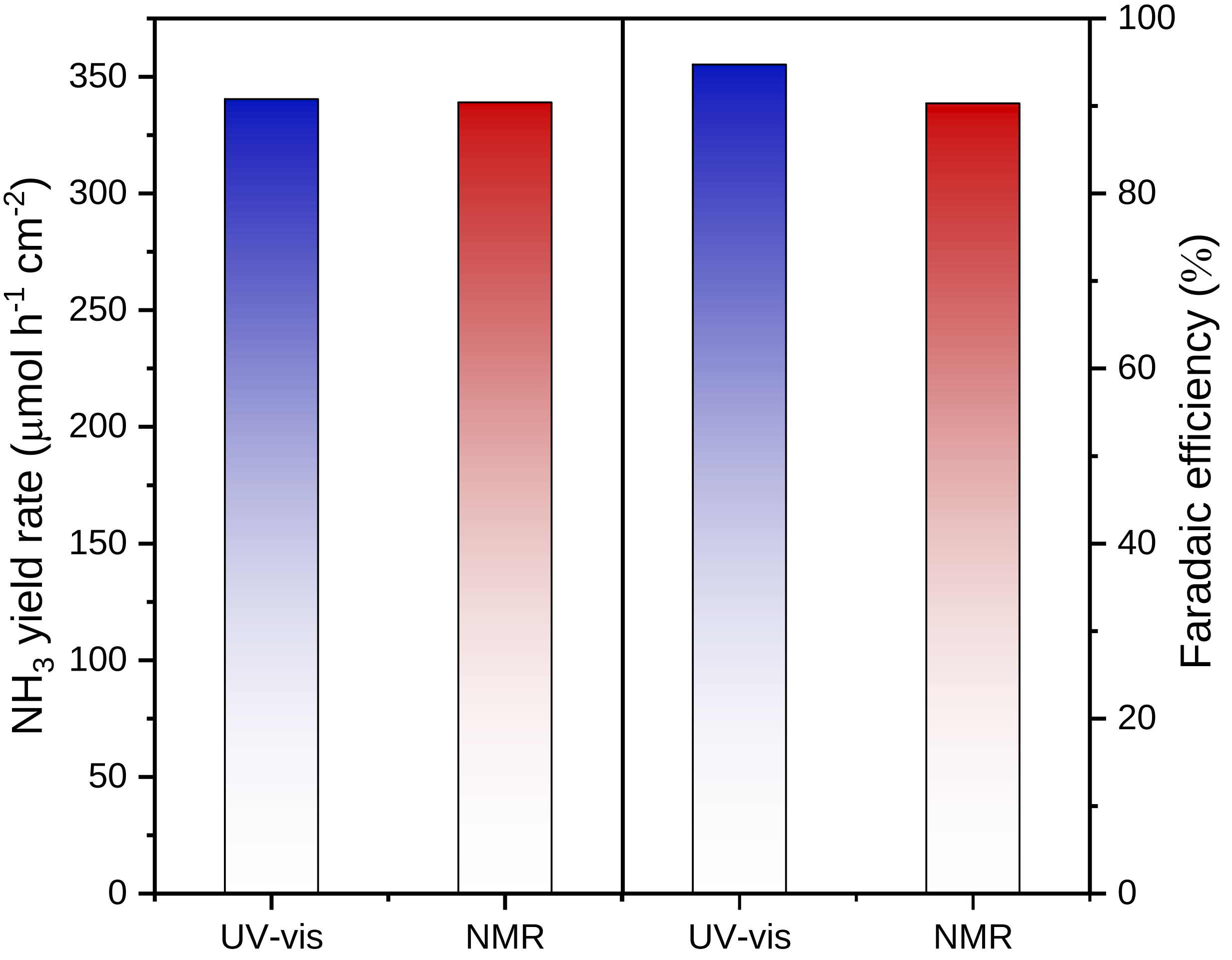

Supplementary Figure 14. Comparison of NH_3 yield rate and FE_{NH_3} over Co/Co_3O_4/CoB after 1h of NO electrolysis at -0.6 V vs. RHE determined by UV-vis and NMR method.

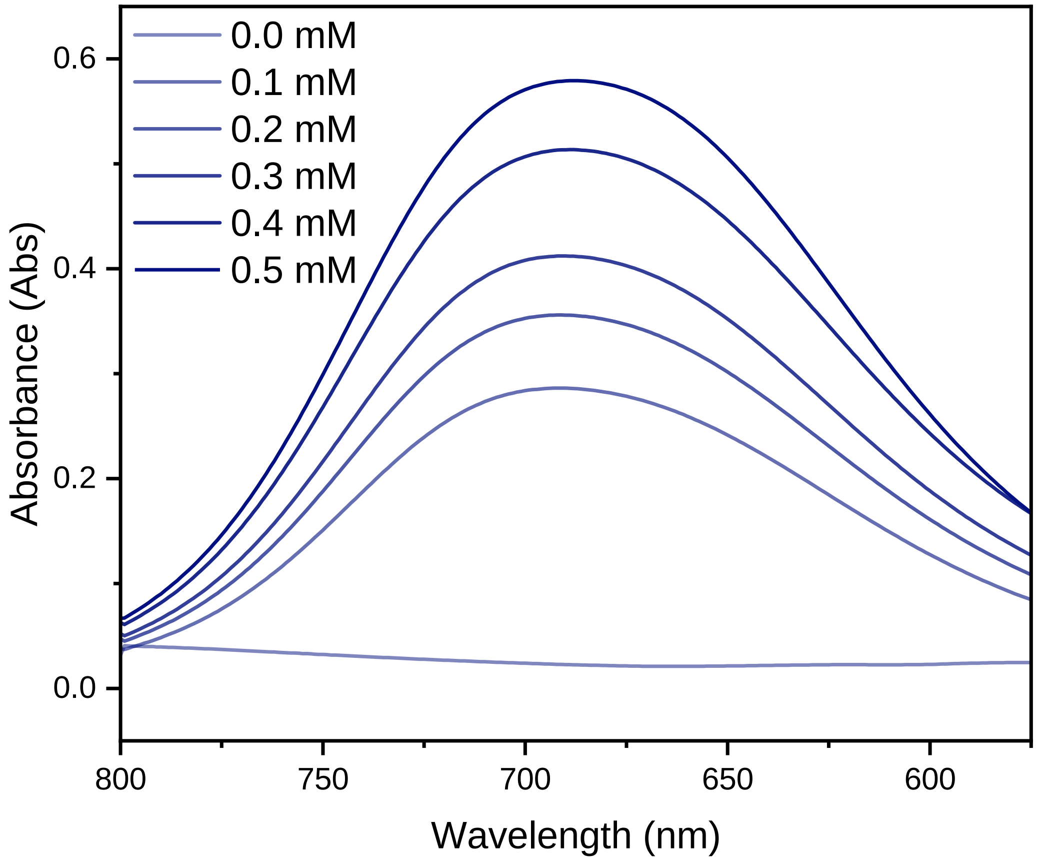

Supplementary Figure 15. UV-vis absorption spectra of NH_2OH solution with concentration in the range of 0-0.5 mM in 0.1 M PBS.

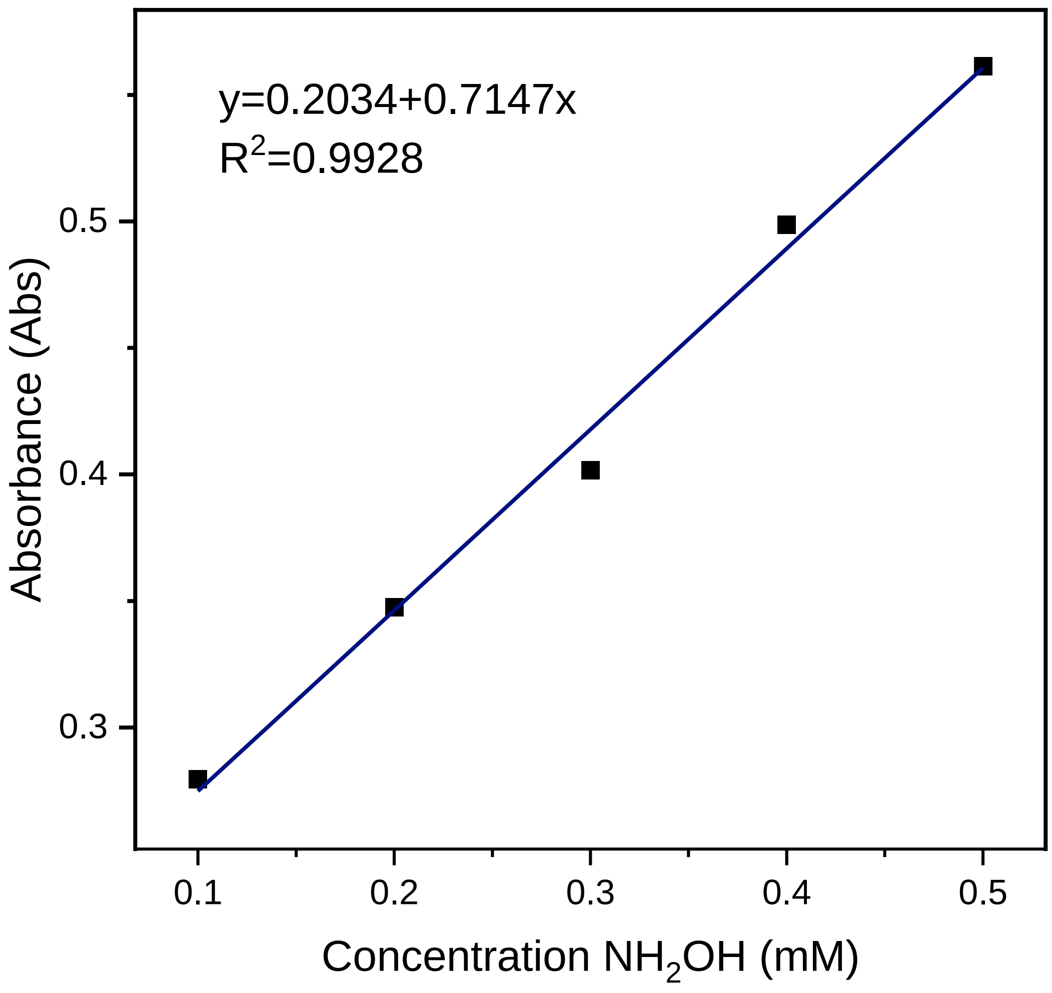

Supplementary Figure 16. The linear calibration curve for quantifying NH_2OH.

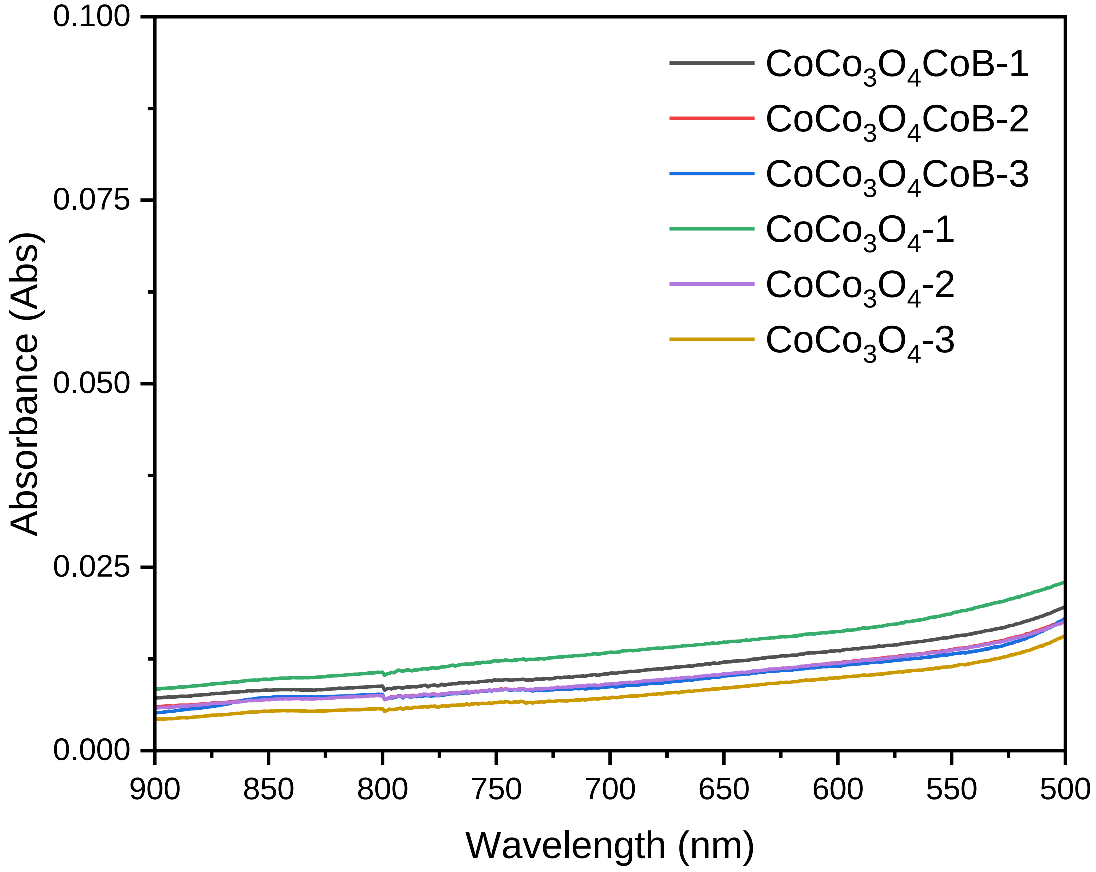

Supplementary Figure 17. UV-vis absorption spectra of the catholyte collected after 1 h of NORR at -0.5 V vs. RHE.

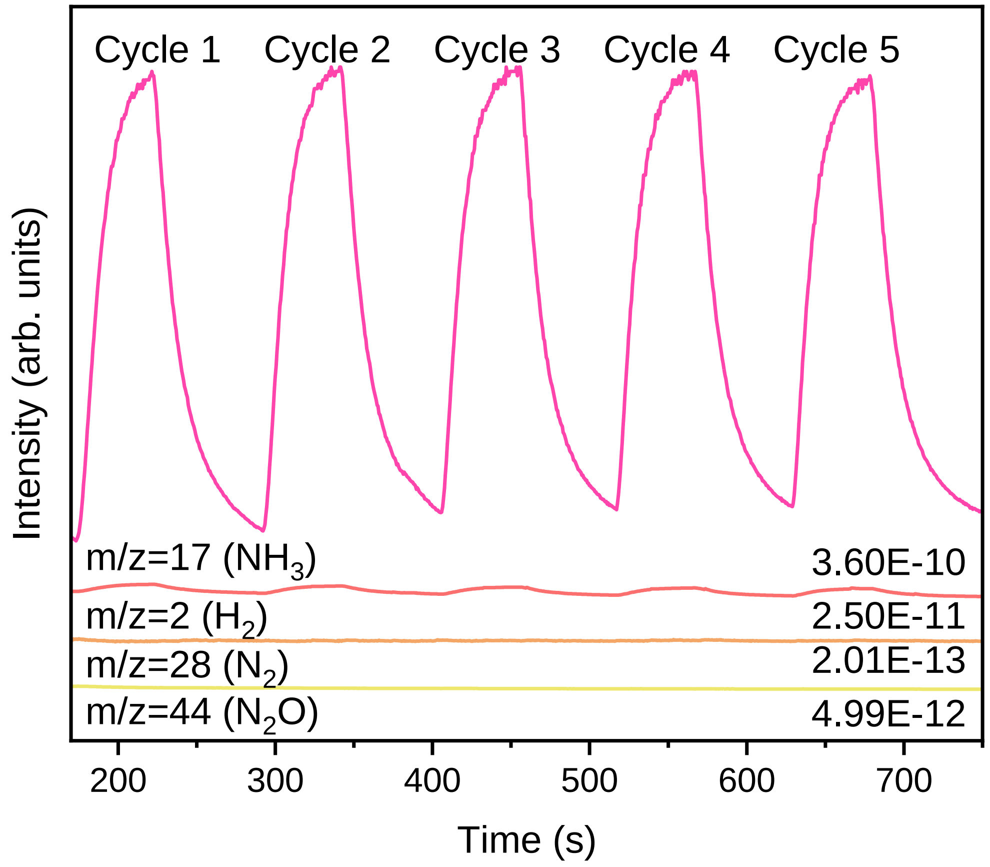

Supplementary Figure 18. The DEMS signals.

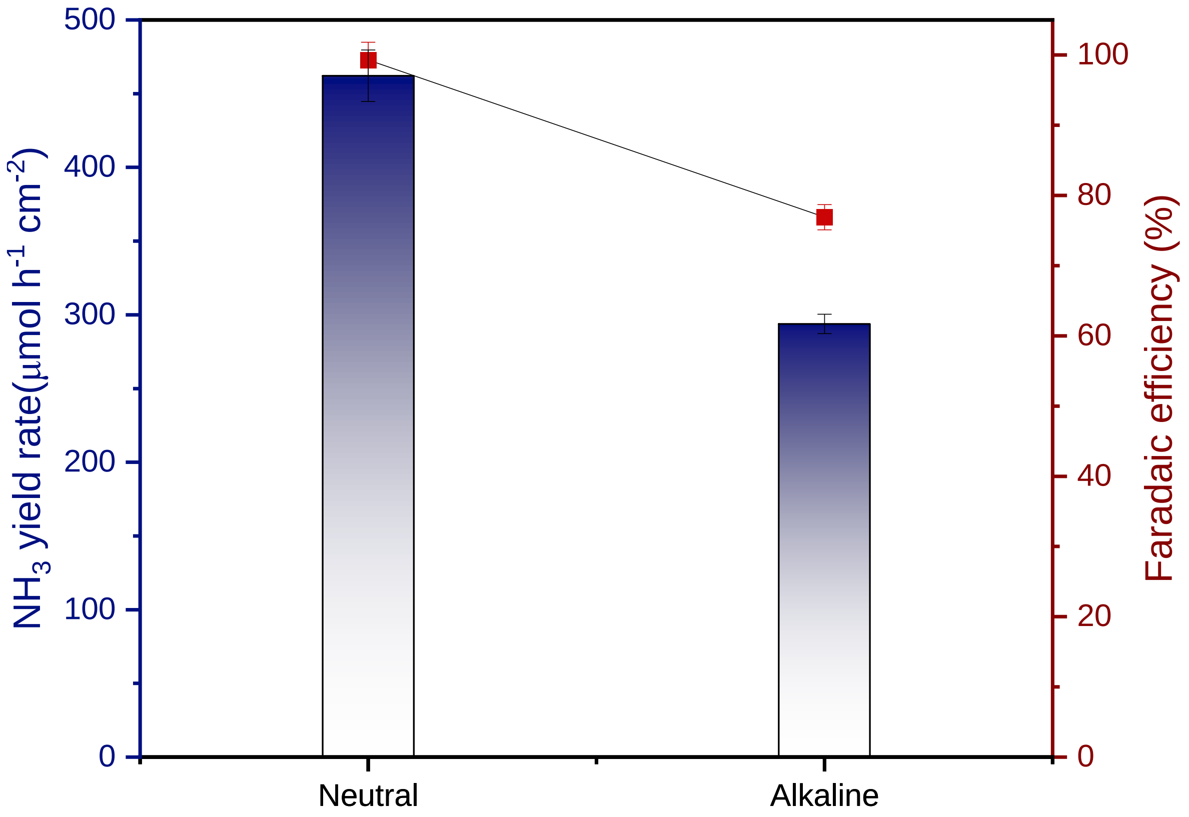

Supplementary Figure 19. The electrochemical NORR performance over $Co/Co_3O_4/CoB$ recorded in 0.1 M PBS and 0.1 M KOH electrolyte. Error bars are determined from three replicate trials at different electrolytes.

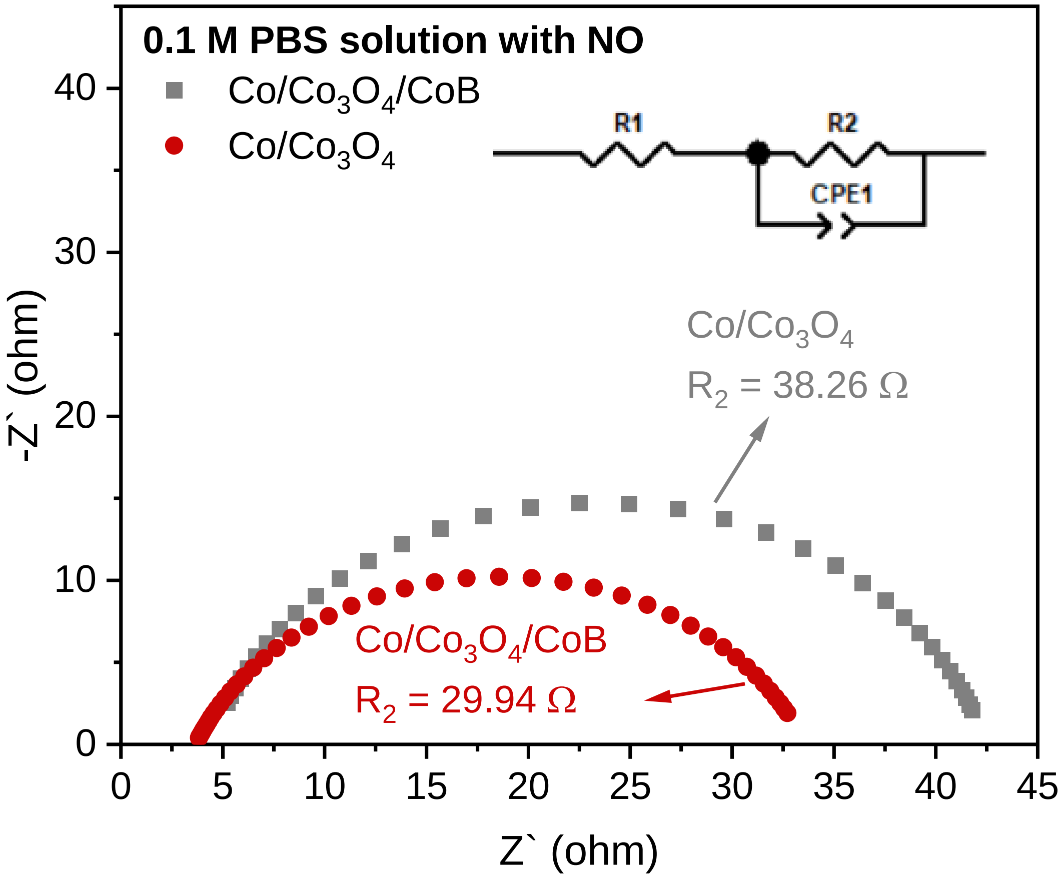

Supplementary Figure 20. EIS spectra of Co/Co_3O_4/CoB and Co/Co_3O_4.

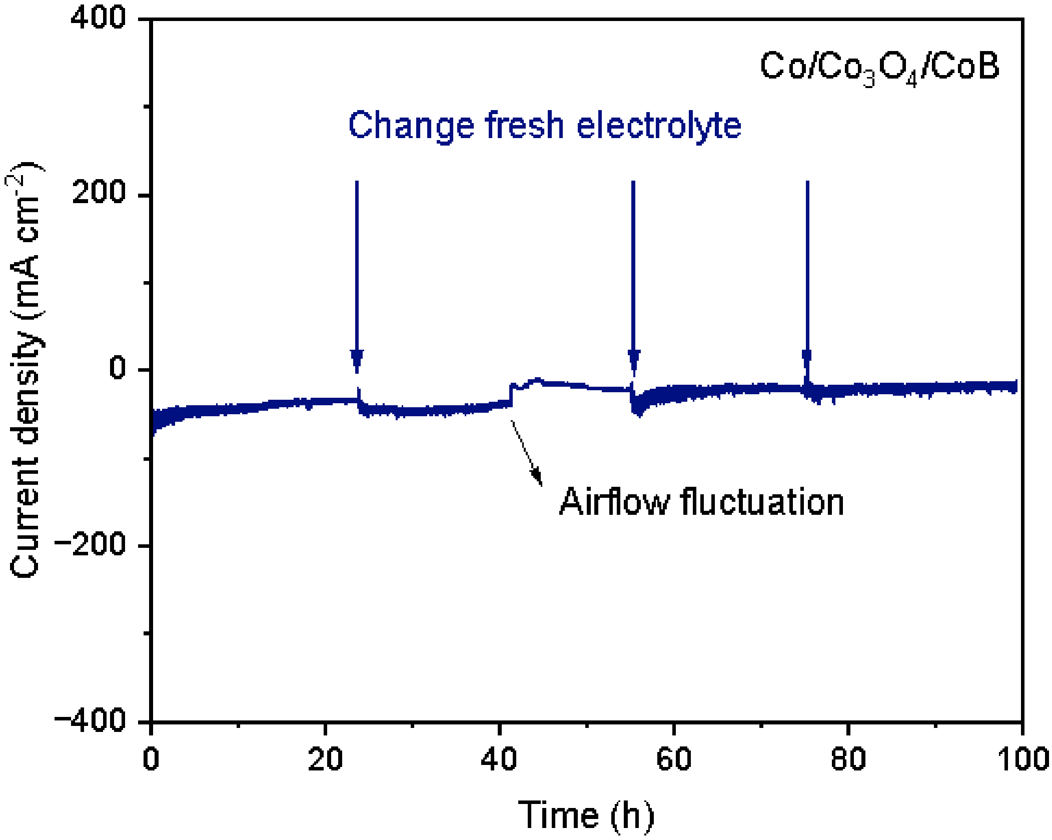

Supplementary Figure 21. The long-term chronoamperometric curve recorded over $Co/Co_3O_4/CoB$ at -0.5 V vs. RHE (The electrolyte was updated every 24 hours. Blue arrows indicate the renewal of fresh electrolyte).

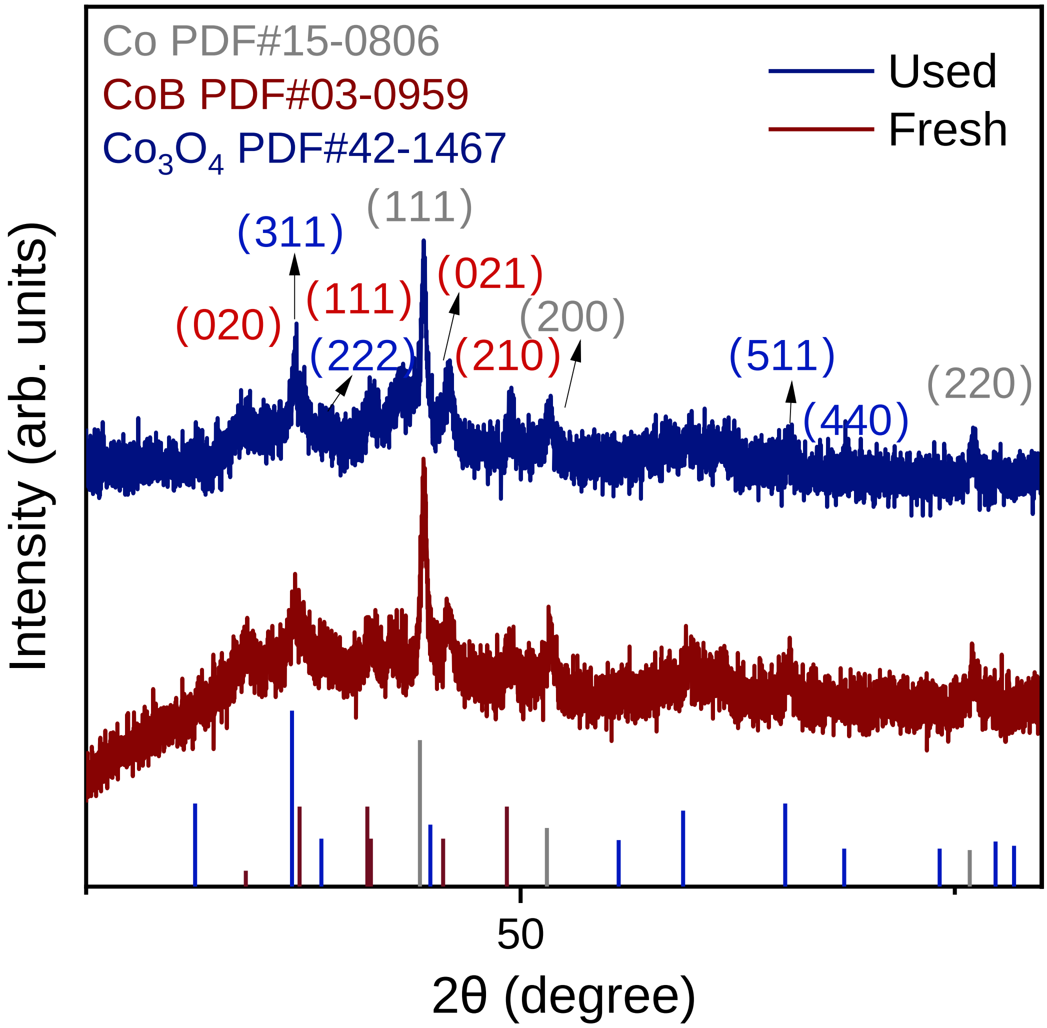

Supplementary Figure 22. XRD patterns of Co/Co_3O_4/CoB before and after NORR durability test.

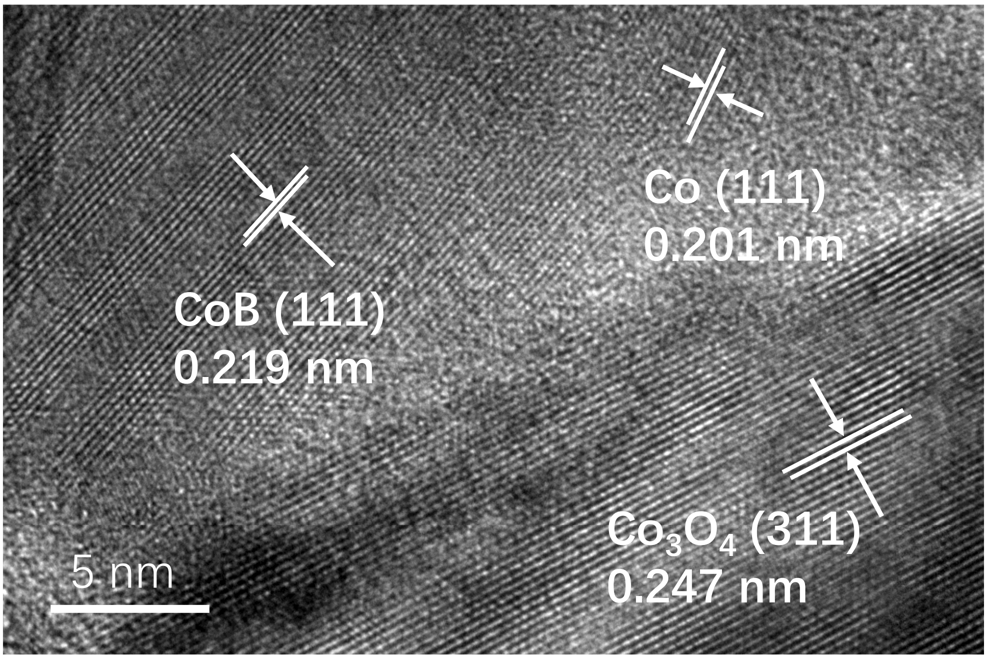

Supplementary Figure 23. The HRTEM image of Co/Co_3O_4/CoB after NORR durability test.

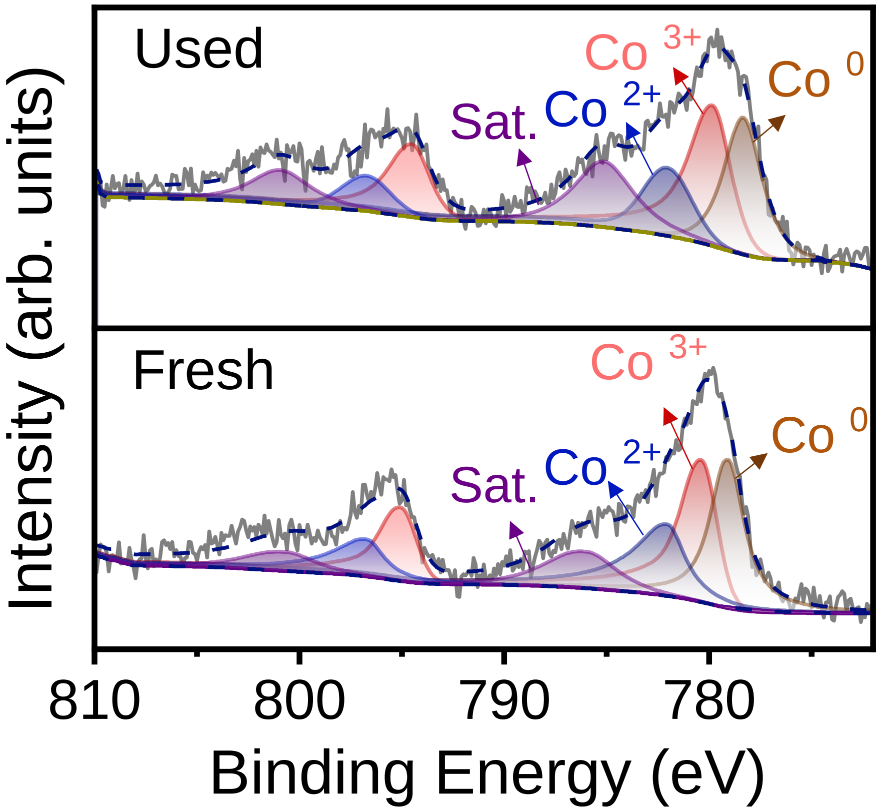

Supplementary Figure 24. Co *2p*$_{3/2}$ XPS spectra of Co/Co_3O_4/CoB before and after NORR durability test.

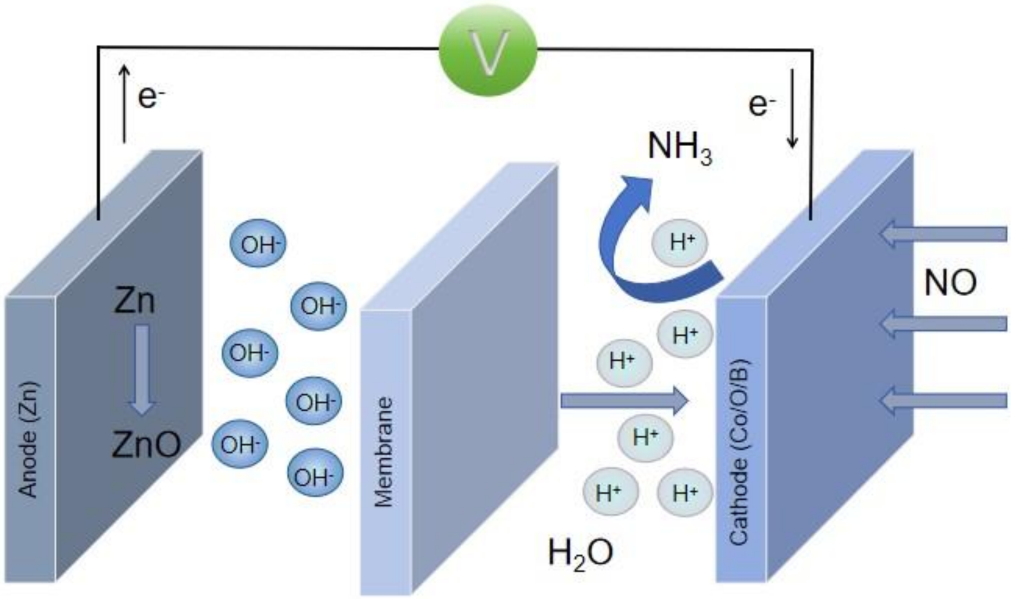

Supplementary Figure 25. Schematic diagram showing the working principle of a Zn-NO battery.

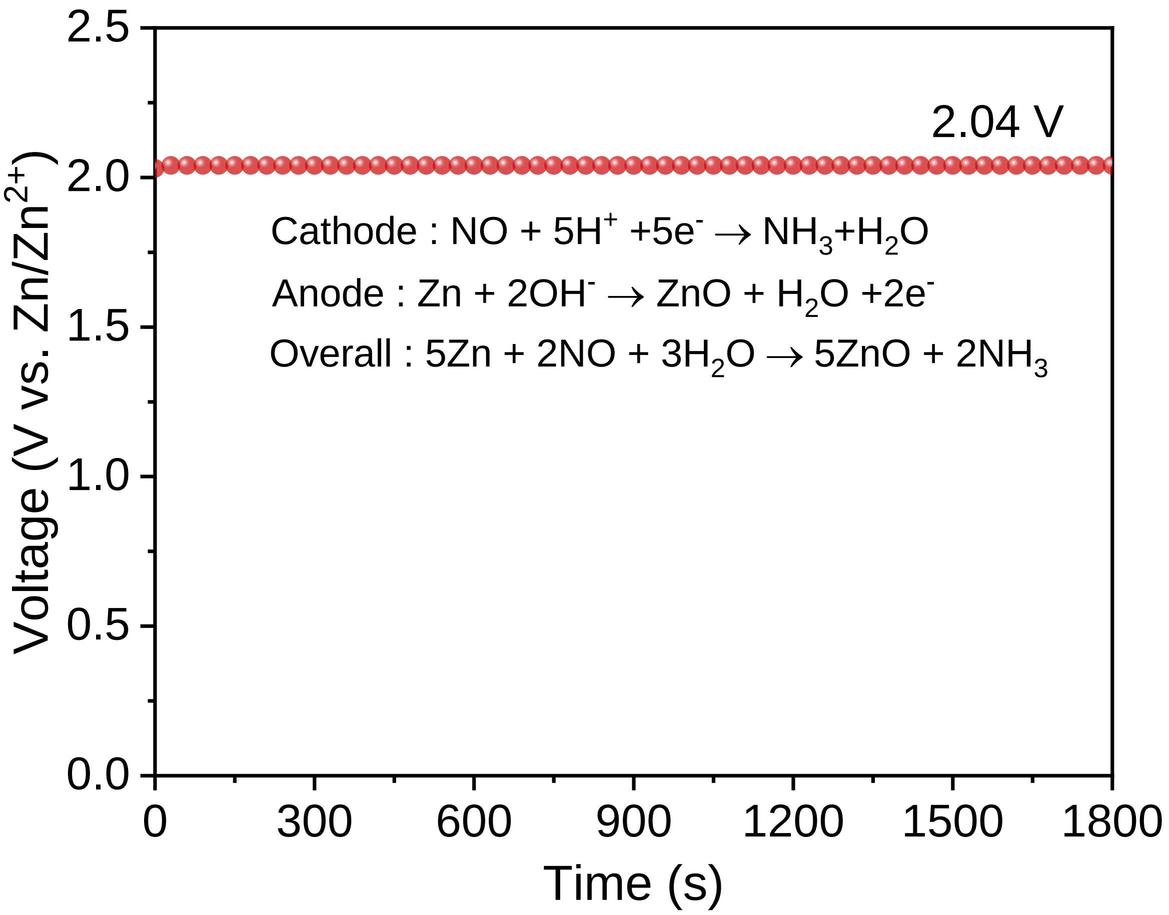

Supplementary Figure 26. OCV of the Zn−NO battery with Co/Co_3O_4/CoB cathode.

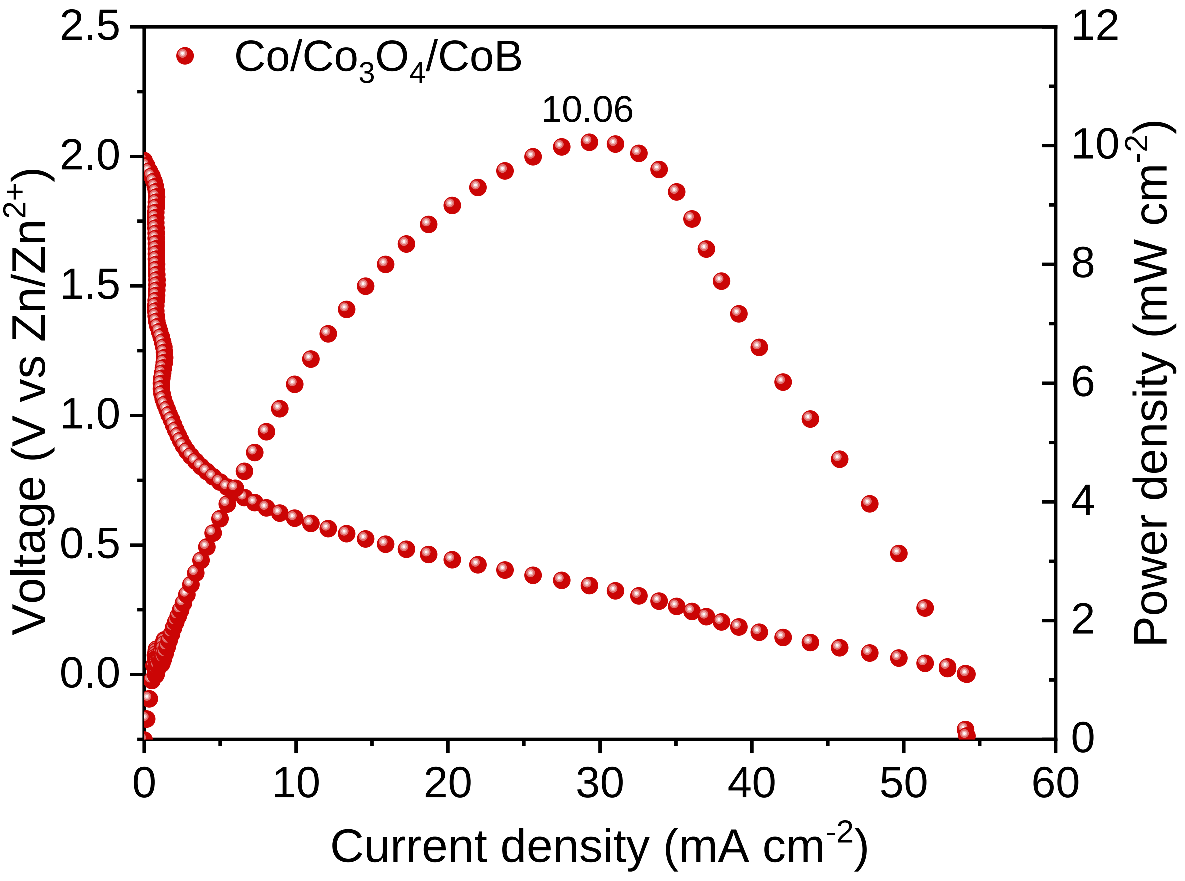

Supplementary Figure 27. Polarization curve and power density plot of the Zn−NO battery with $Co/Co_3O_4/CoB$ cathode.

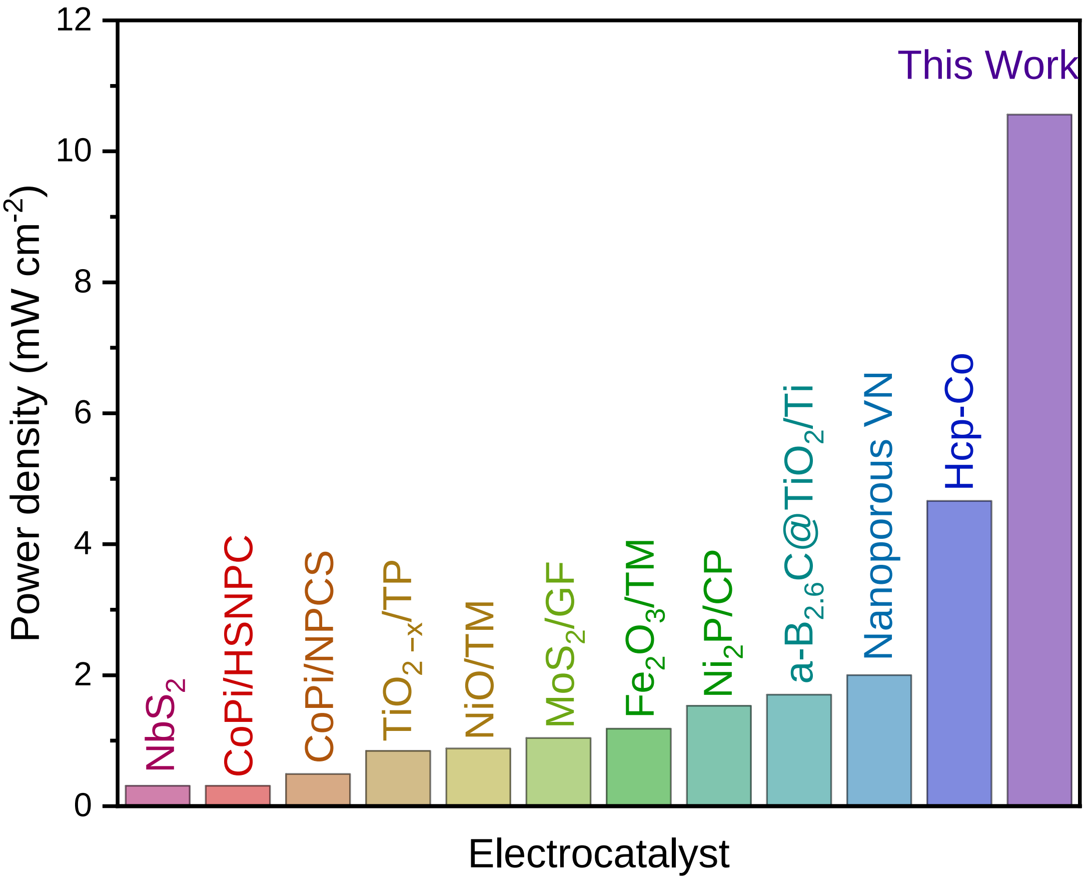

Supplementary Figure 28. Comparison of the Zn-NO battery performance.

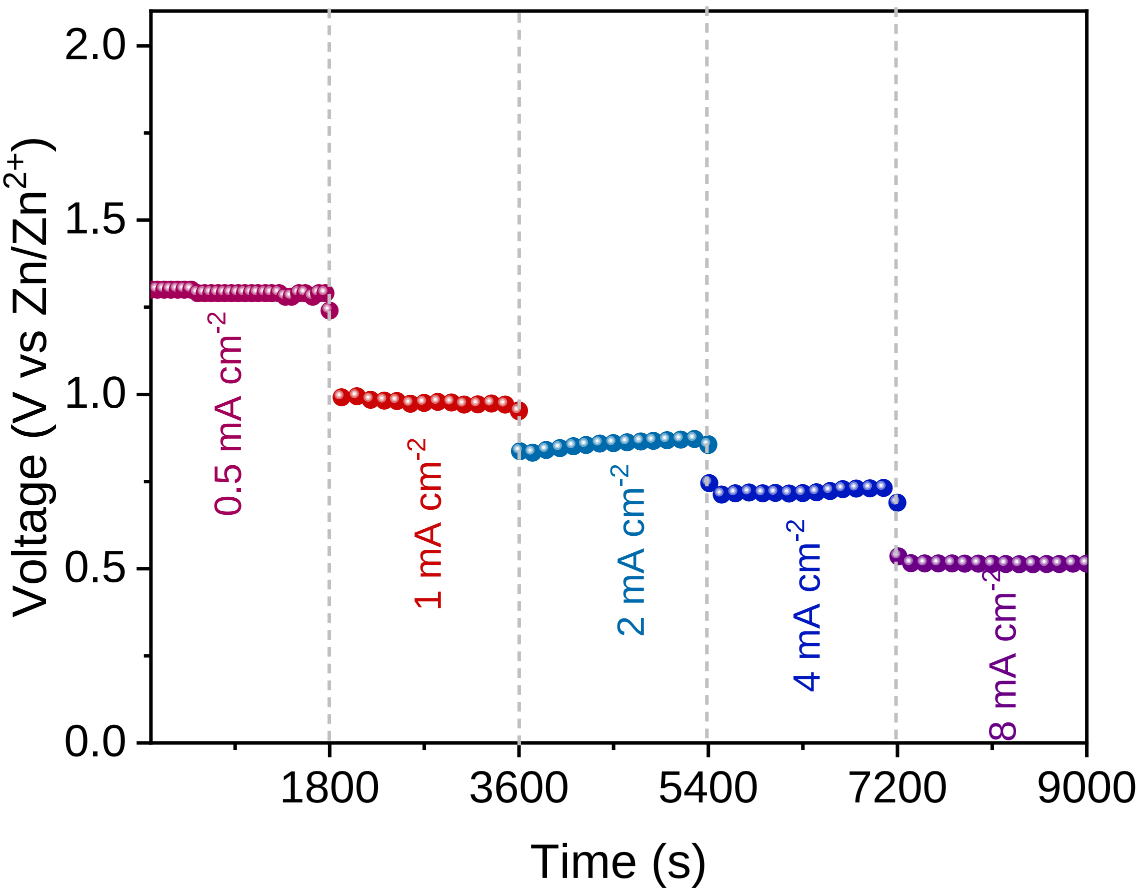

Supplementary Figure 29. Discharging tests at various current densities.

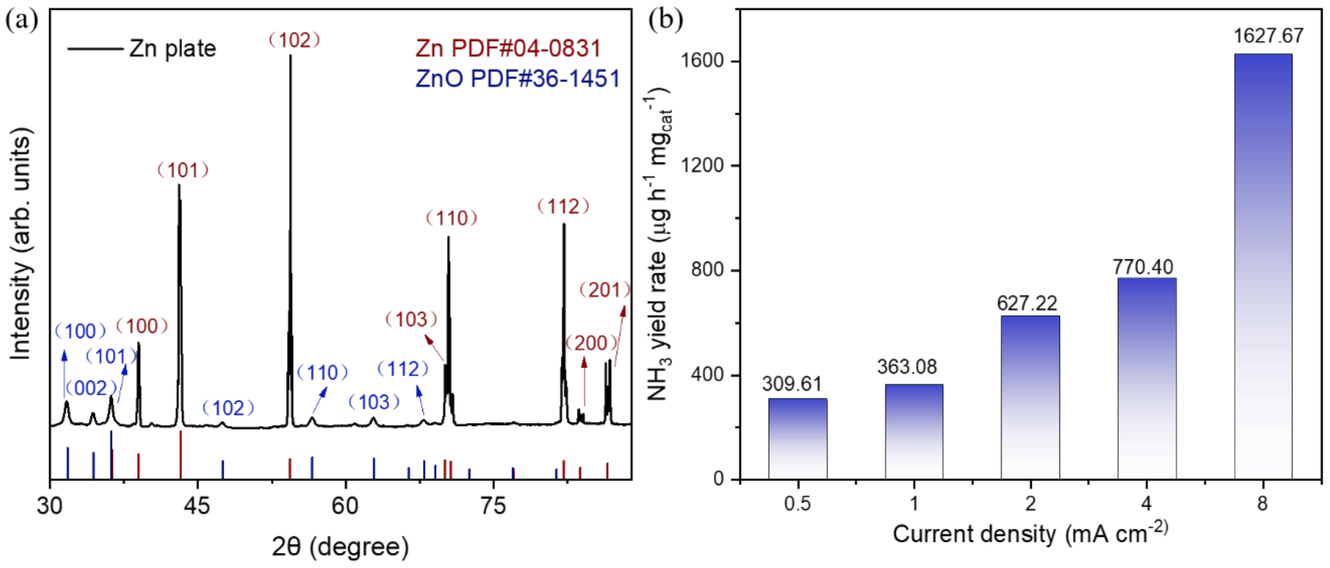

Supplementary Figure 30. a. The XRD pattern of used Zn plate from Zn-NO battery test. The XRD result indicated that ZnO was produced on the Zn plate after Zn-NO battery testing. **b.** Ammonia production in the Zn-NO battery at different current densities.

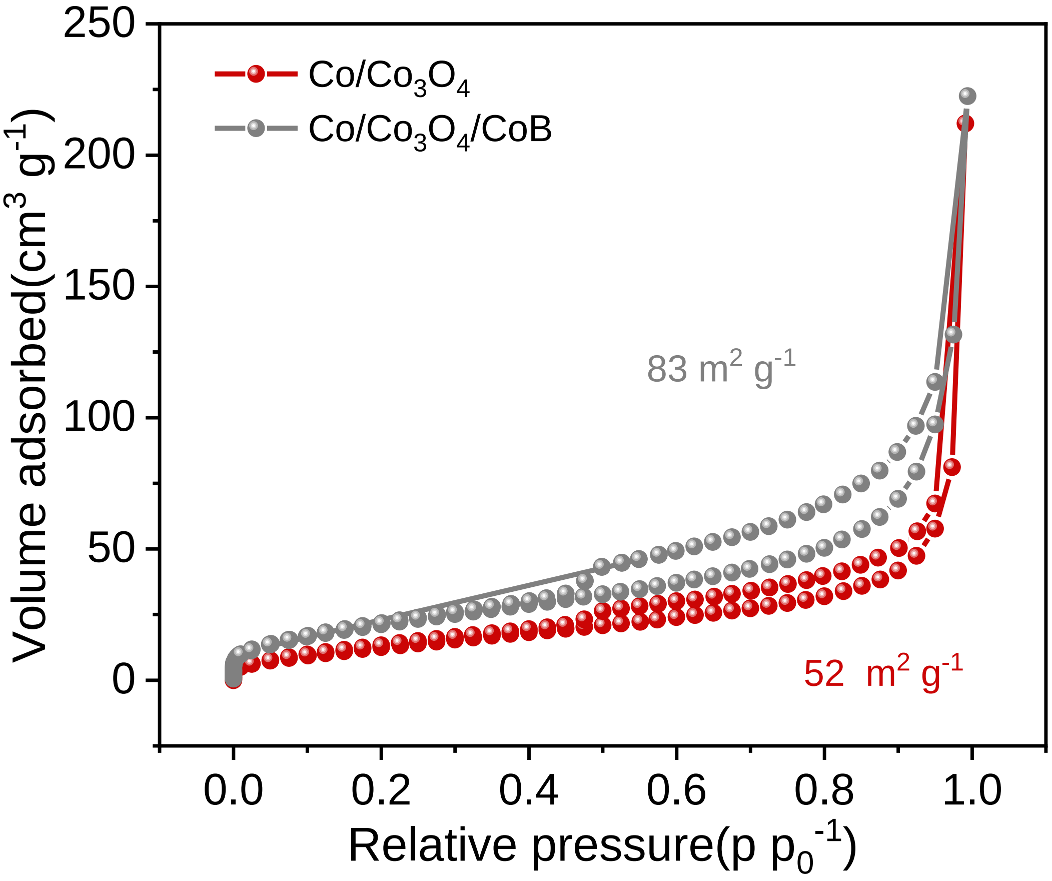

Supplementary Figure 31. N_2 adsorption-desorption isotherms of Co/Co_3O_4/CoB and Co/Co_3O_4.

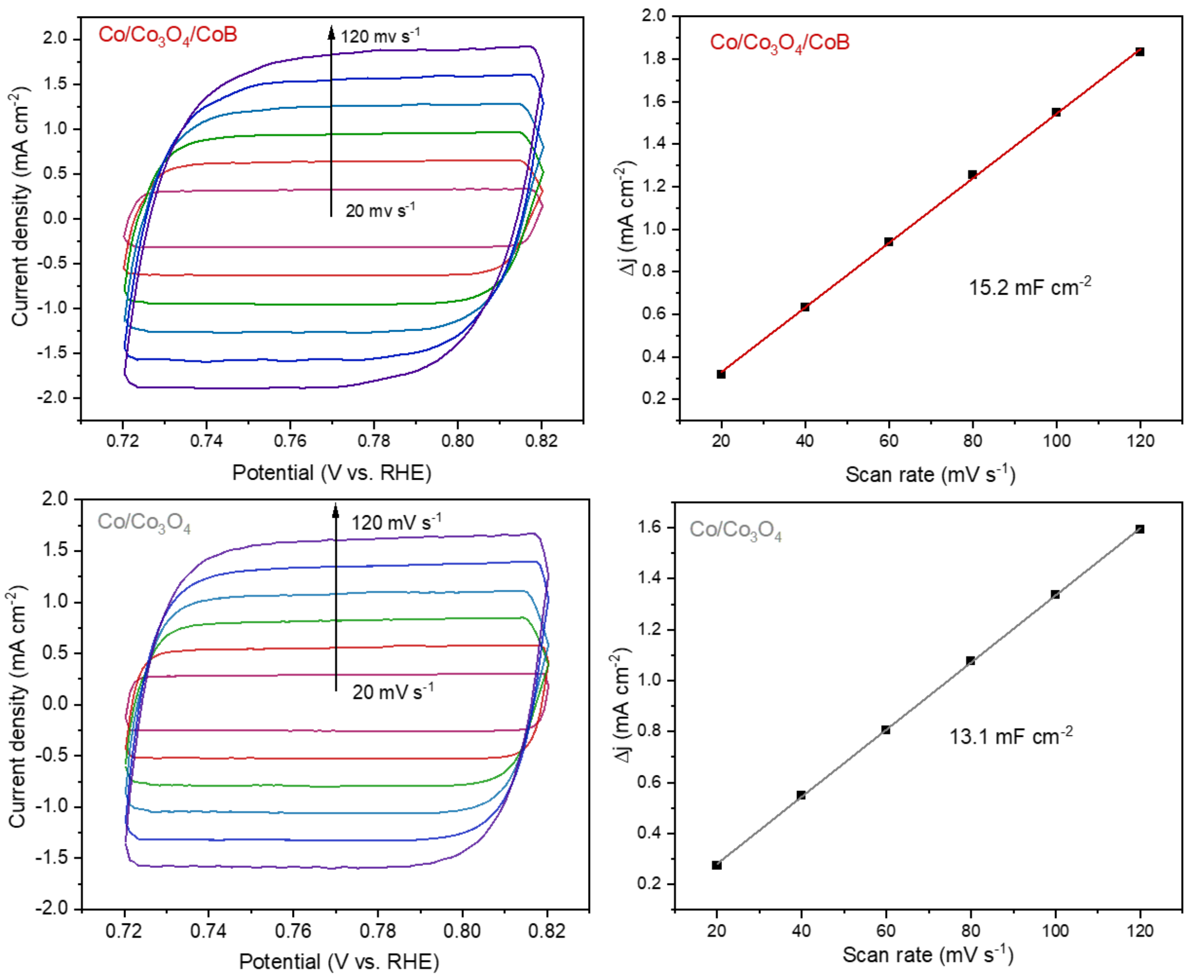

Supplementary Figure 32. CV measurements conducted at different scanning rates in the range of 20 ~ 120 mV s^{-1} for determining the electrochemically active surface area of $Co/Co_3O_4/CoB$ and Co/Co_3O_4.

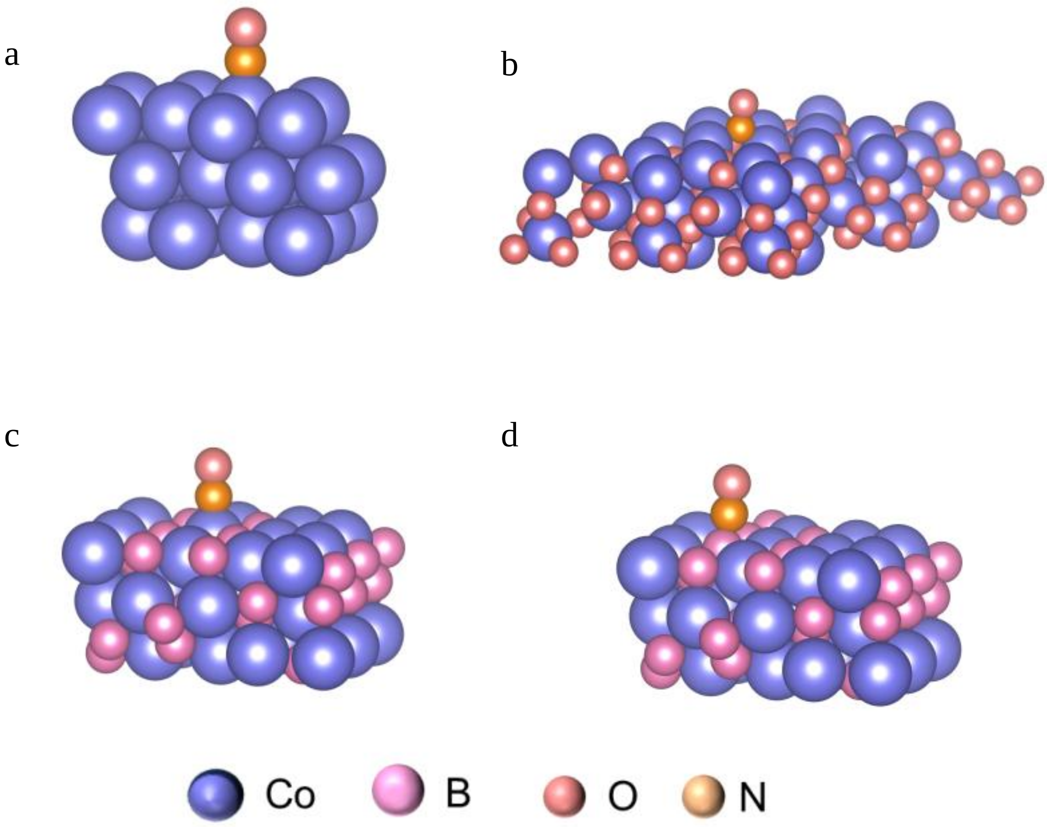

Supplementary Figure 33. Schematic diagrams showing the calculation models for NO adsorption energy of nitrogen end on Co site of Co (a) and Co_3O_4 (b), as well as Co and B sites on CoB (c & d).

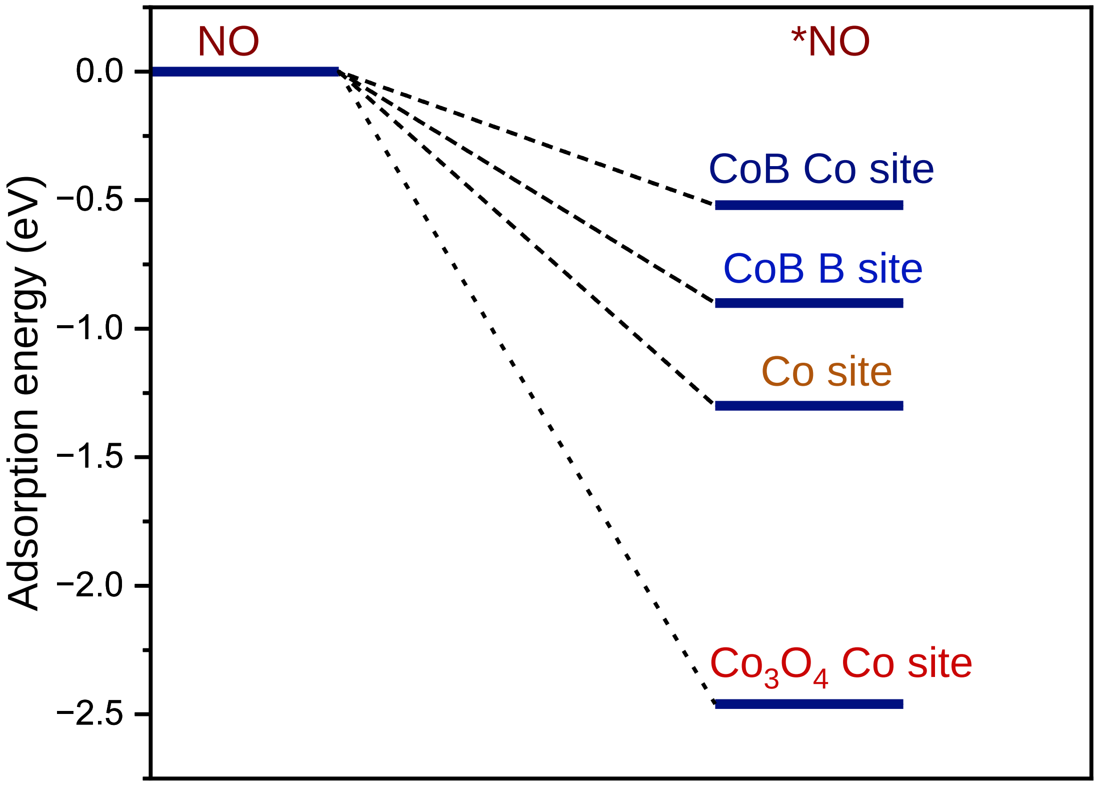

Supplementary Figure 34. The calculated NO adsorption energy of oxygen end on Co (111), CoB (021) and Co_3O_4 (311).

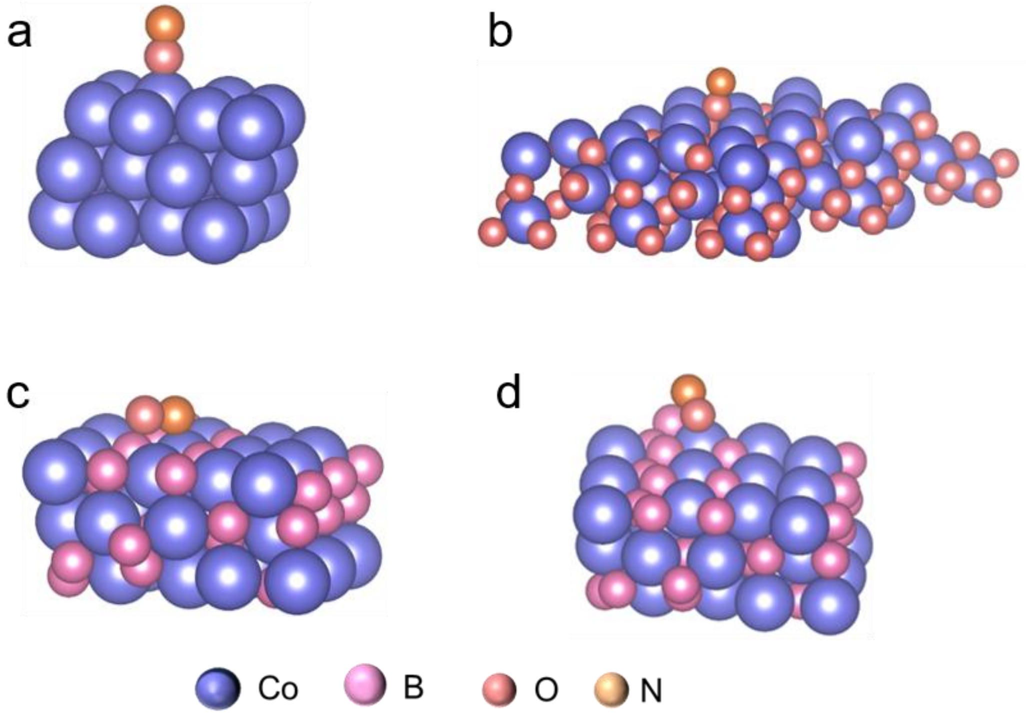

Supplementary Figure 35. Schematic diagrams showing the calculation models for NO adsorption energy of oxygen end on Co site of Co (a) and Co_3O_4 (b), as well as B and Co sites on CoB (c & d).

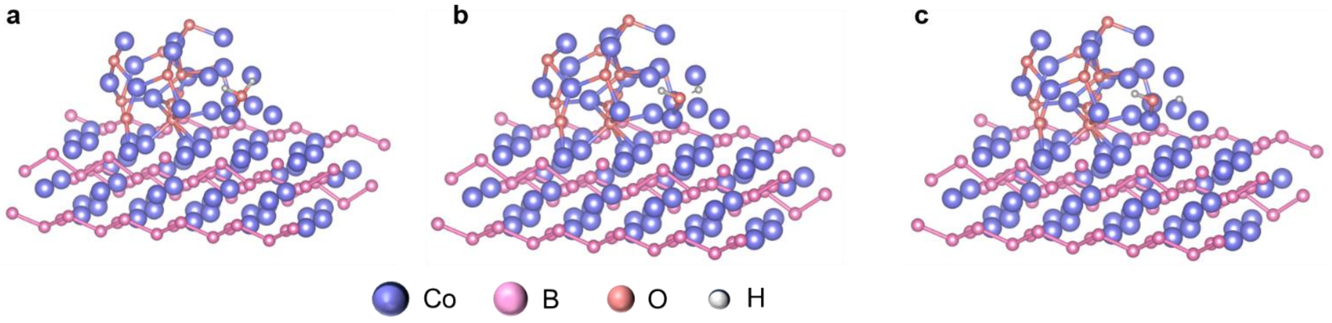

Supplementary Figure 36. Water dissociation on Co/Co_3O_4/CoB. (a) initial state (IS), (b) transition state (TS), (c) final state (FS).

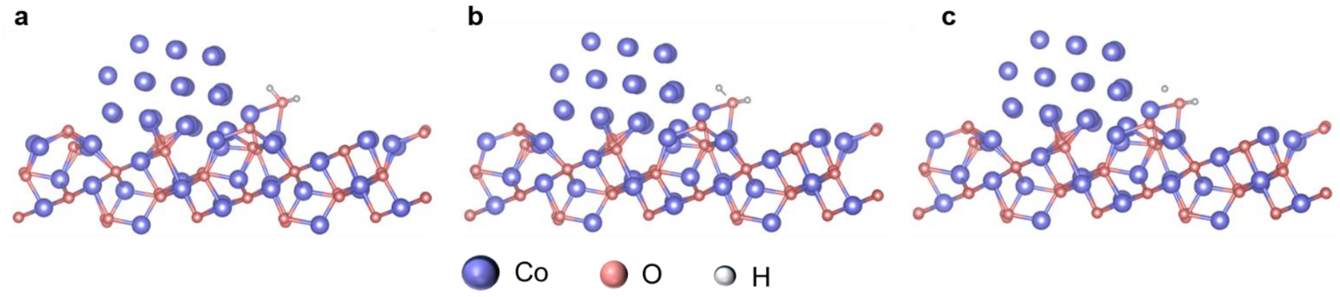

Supplementary Figure 37. Water dissociation on Co/Co_3O_4. (a) initial state (IS), (b) transition state (TS), (c) final state (FS).

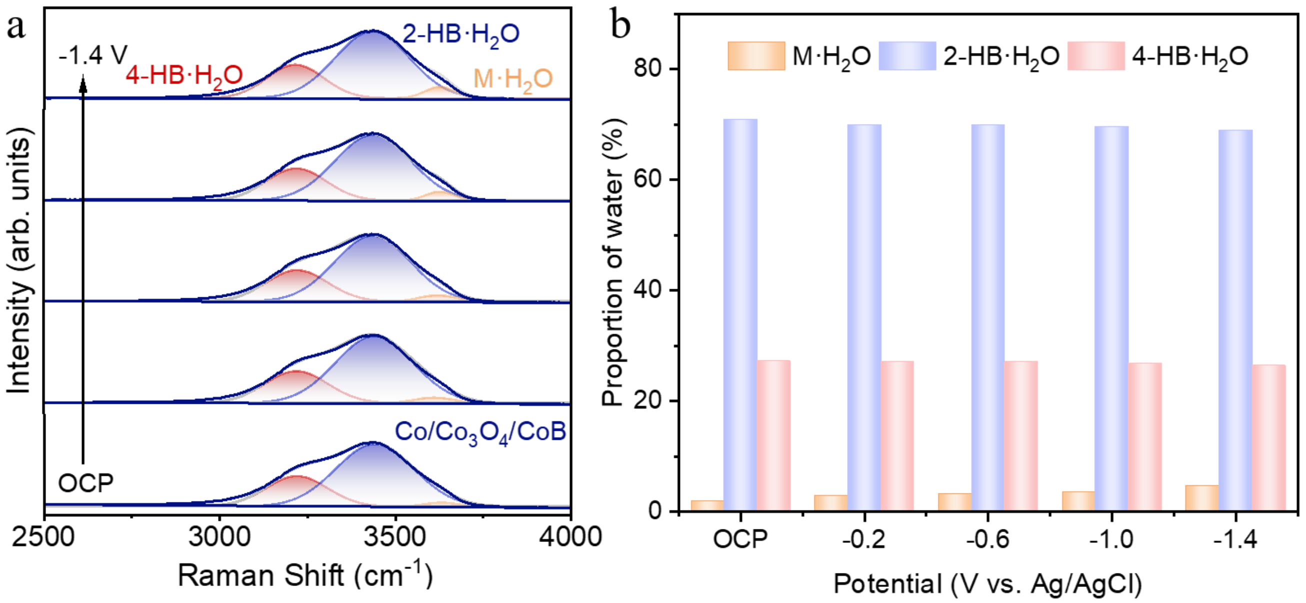

Supplementary Figure 38. (a) The *in-situ* Raman spectra recorded over Co/Co_3O_4/CoB at different applied cathodic potentials (M refers to Na^+ or K^+). (b) The proportion of various water over Co/Co_3O_4/CoB at different applied cathodic potentials.

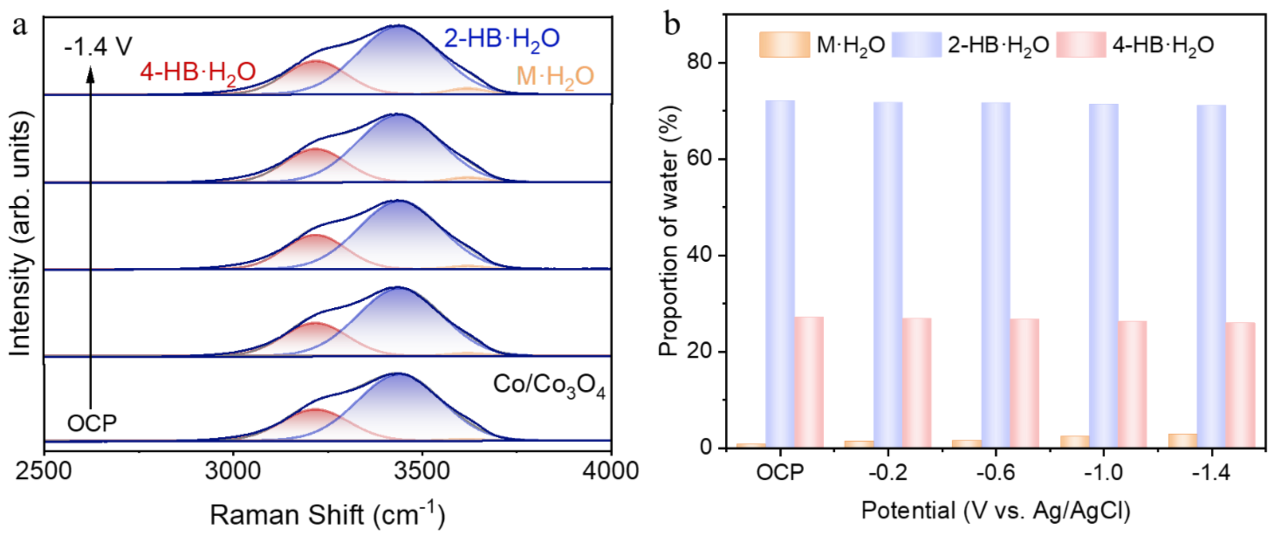

Supplementary Figure 39. (a) The *in-situ* Raman spectra recorded over Co/Co_3O_4 at different applied cathodic potentials (M refers to Na^+ or K^+). (b) The proportion of various water over Co/Co_3O_4 at different applied cathodic potentials.

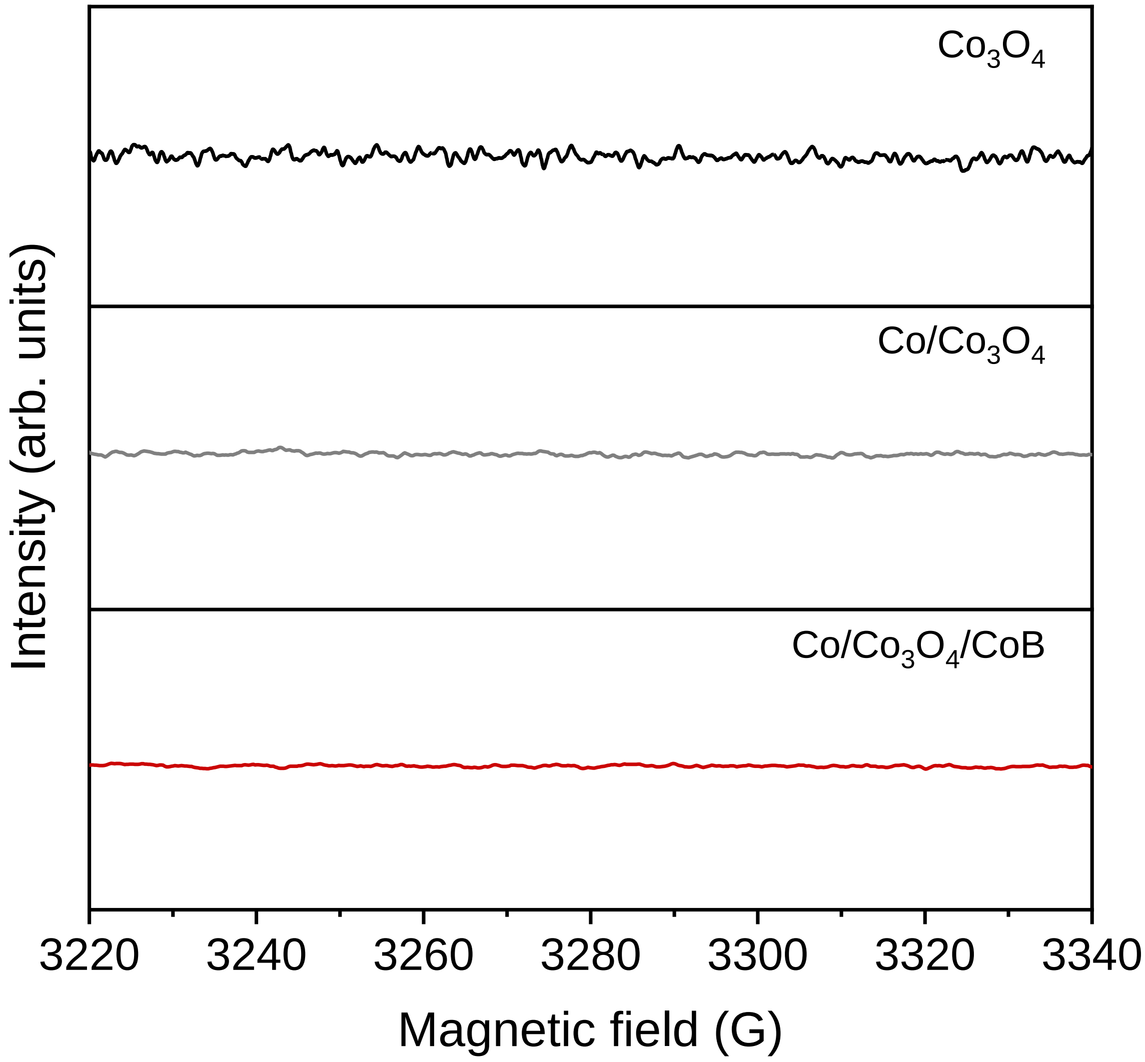

Supplementary Figure 40. EPR spectra recorded over $Co/Co_3O_4/CoB$, Co/Co_3O_4 and Co_3O_4 upon NO electrolysis.

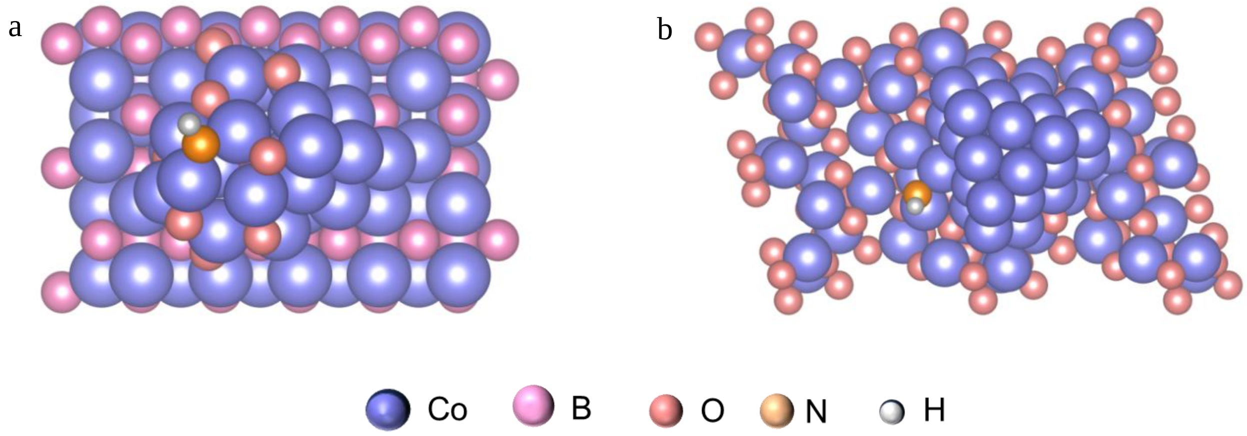

Supplementary Figure 41. The adsorption model of *NH on Co/Co_3O_4/CoB (a) and Co/Co_3O_4 (b).

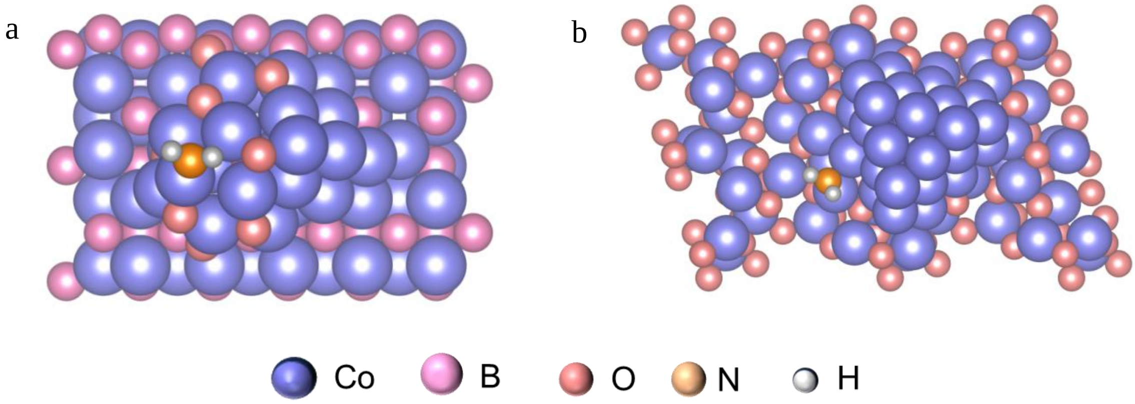

Supplementary Figure 42. The adsorption model of $*NH_2$ on $Co/Co_3O_4/CoB$ (a) and Co/Co_3O_4 (b).

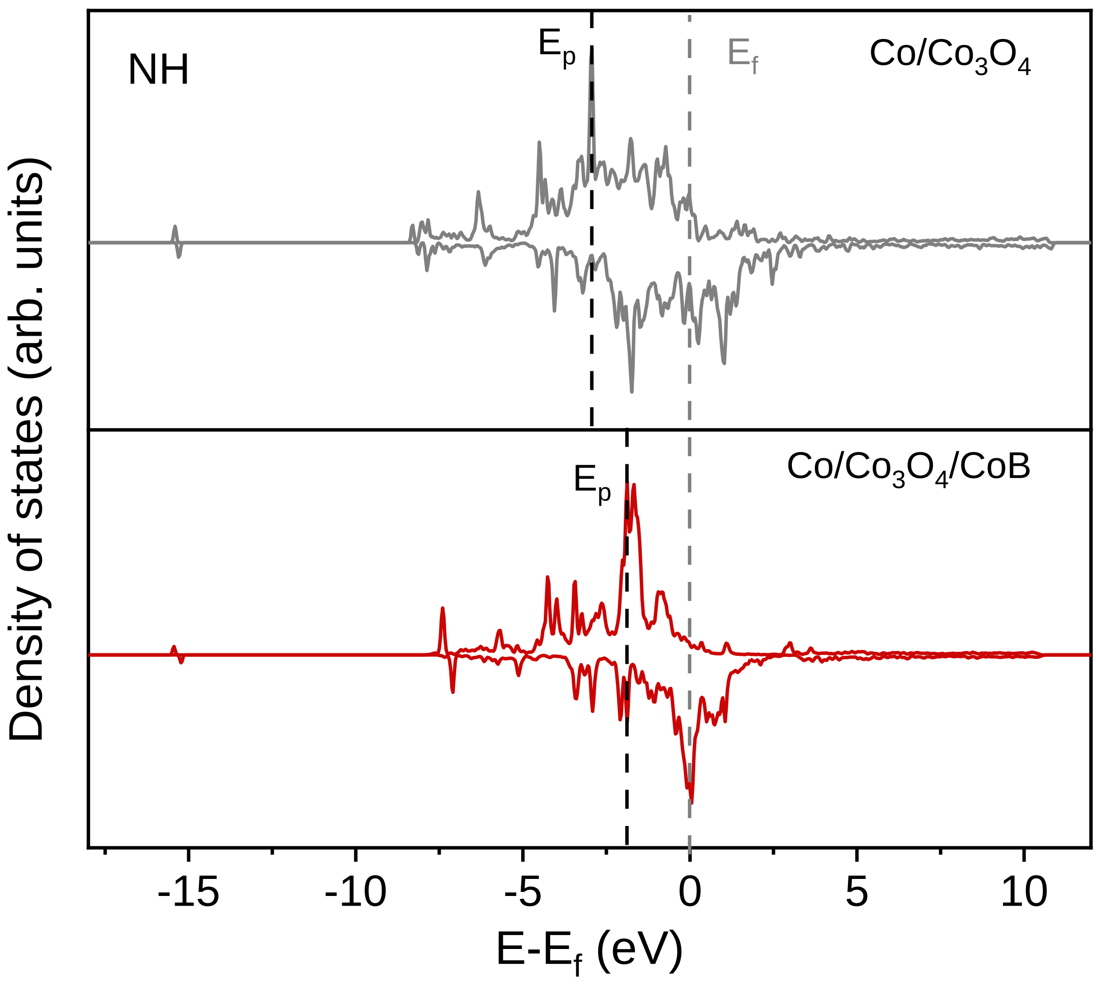

Supplementary Figure 43. The PDOS of *NH on $Co/Co_3O_4/CoB$ and Co/Co_3O_4.

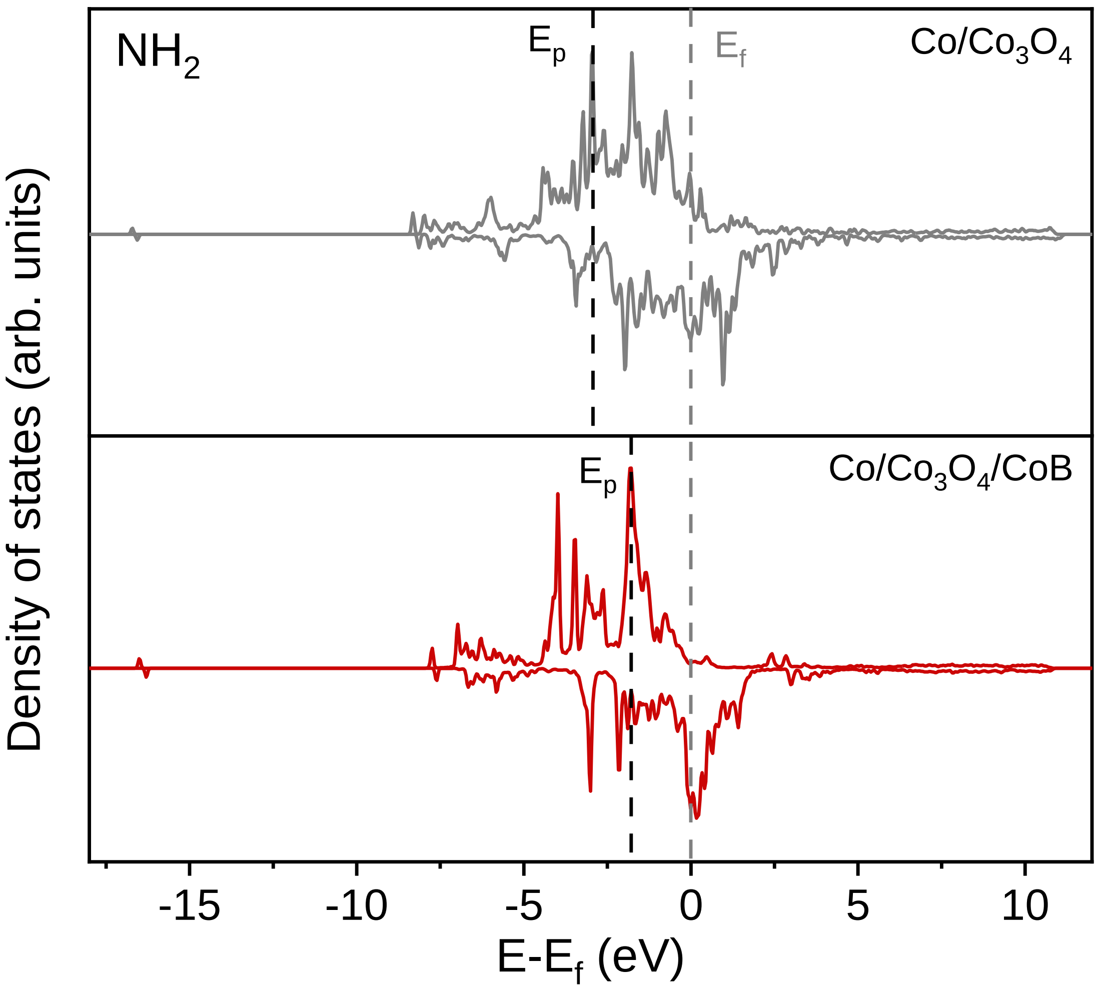

Supplementary Figure 44. The PDOS of $*NH_2$ on $Co/Co_3O_4/CoB$ and Co/Co_3O_4.

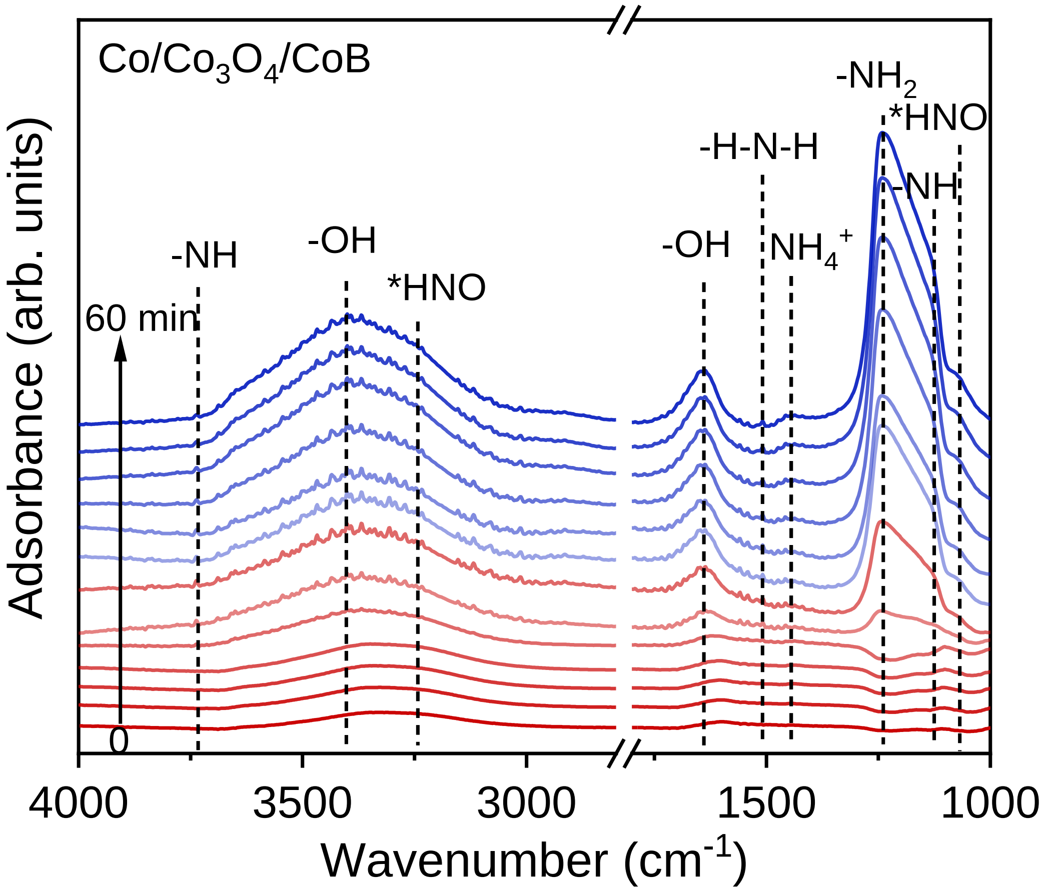

Supplementary Figure 45. *In-situ* ATR-SEIRAS spectra recorded over Co/Co_3O_4/CoB during NORR at -0.5 V vs. RHE in 0.1 M PBS.

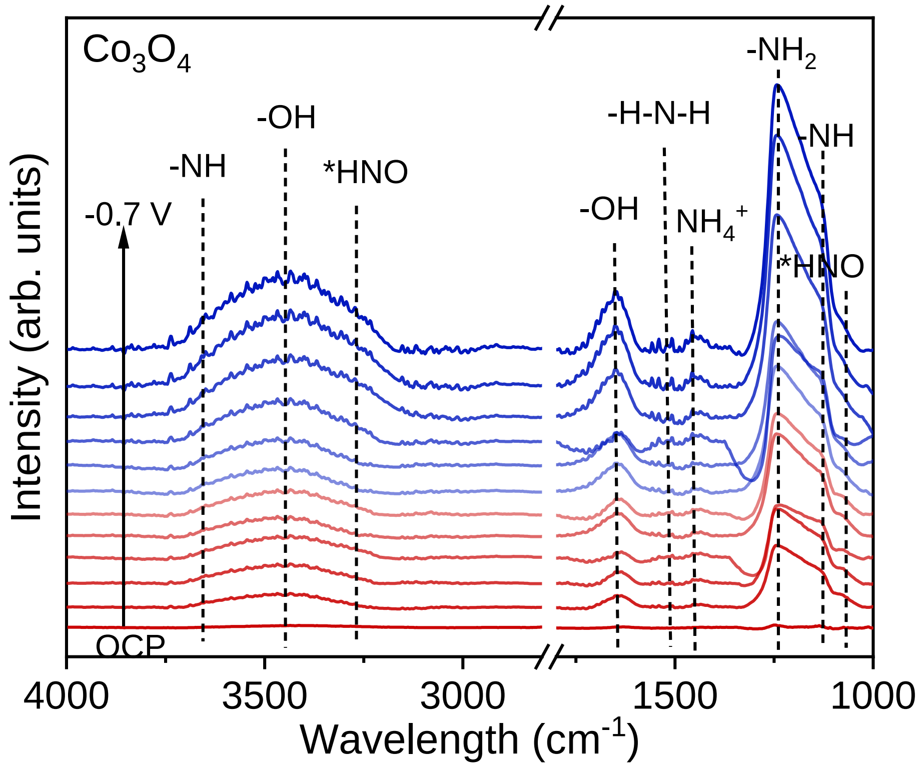

Supplementary Figure 46. *In-situ* ATR-SEIRAS spectra recorded over Co_3O_4 in 0.1 M PBS at the applied cathodic potential from OCP to -0.7 V vs. RHE.

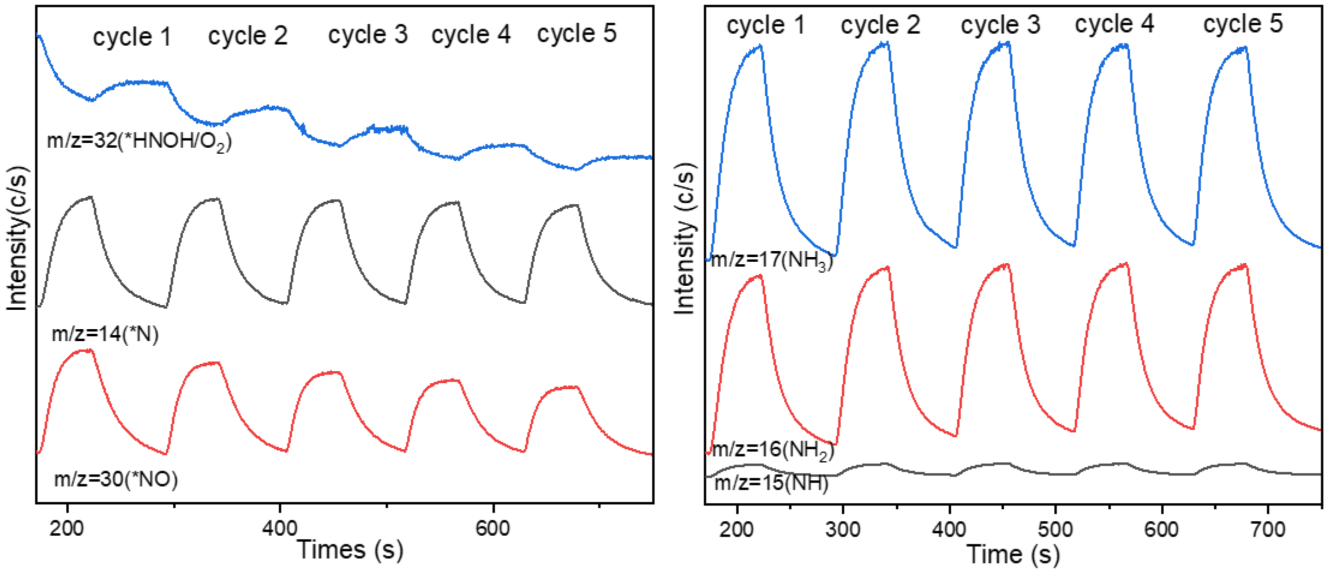

Supplementary Figure 47. Online DEMS measurements conducted over $Co/Co_3O_4/CoB$ for NORR.

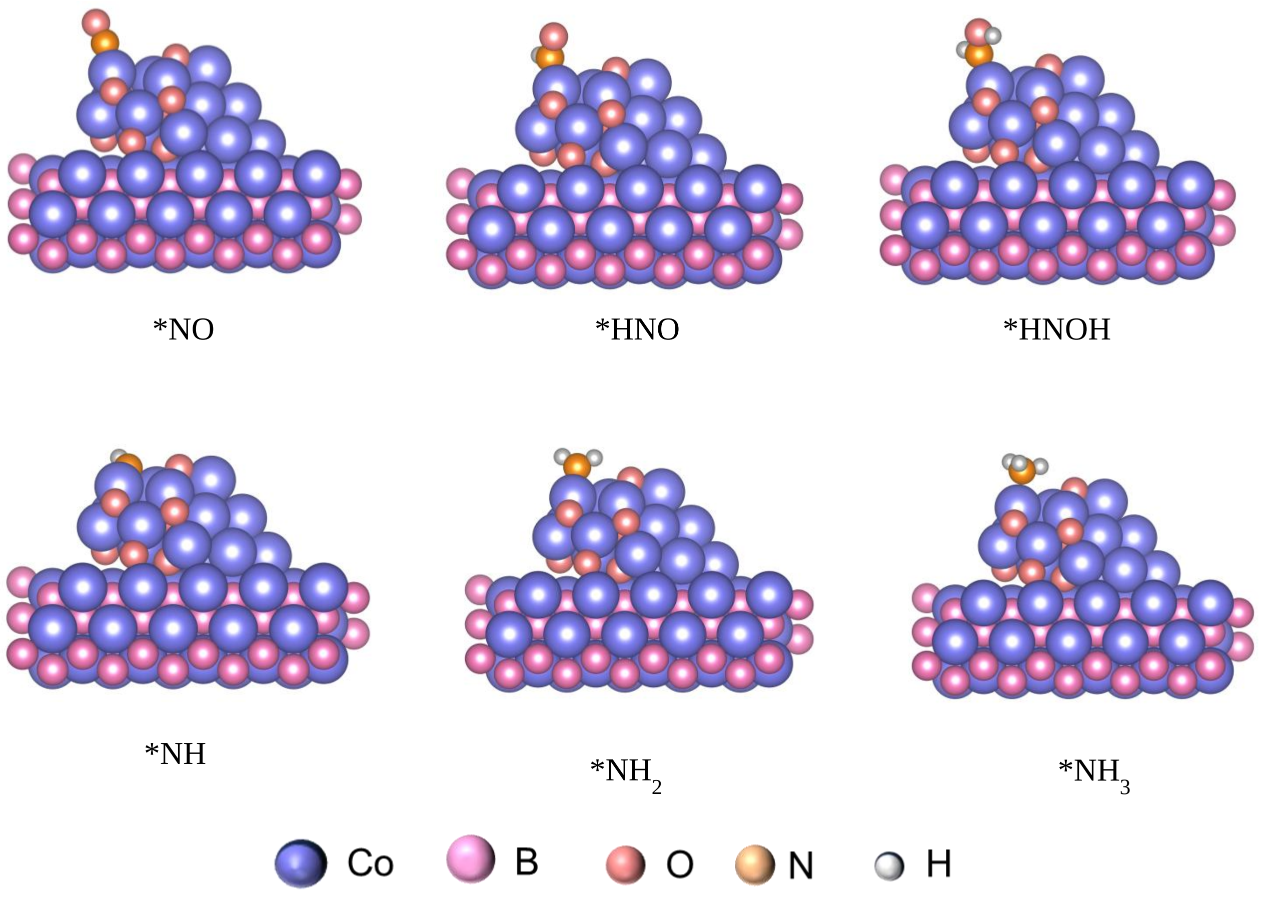

Supplementary Figure 48. Structural models showing NORR on Co/Co_3O_4/CoB.

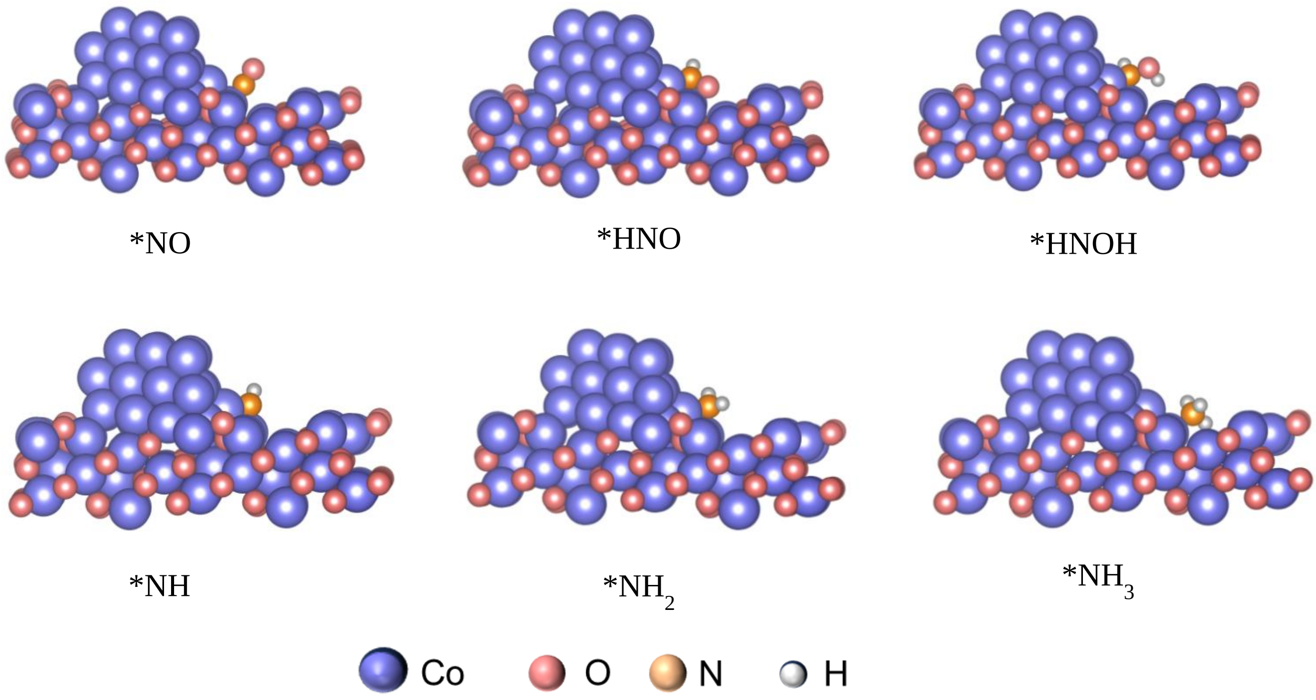

Supplementary Figure 49. Structural models showing NORR on Co/Co_3O_4.

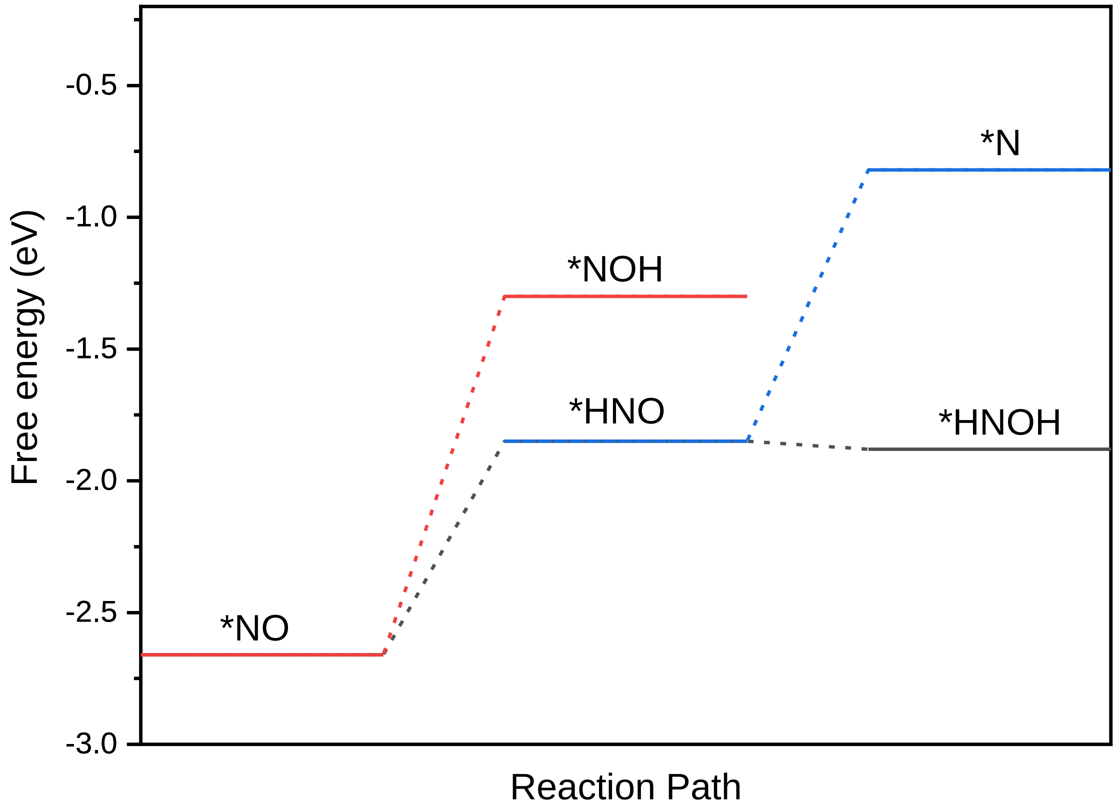

Supplementary Figure 50. Reaction free energy of first and second protonation of *NO on Co/Co_3O_4/CoB.

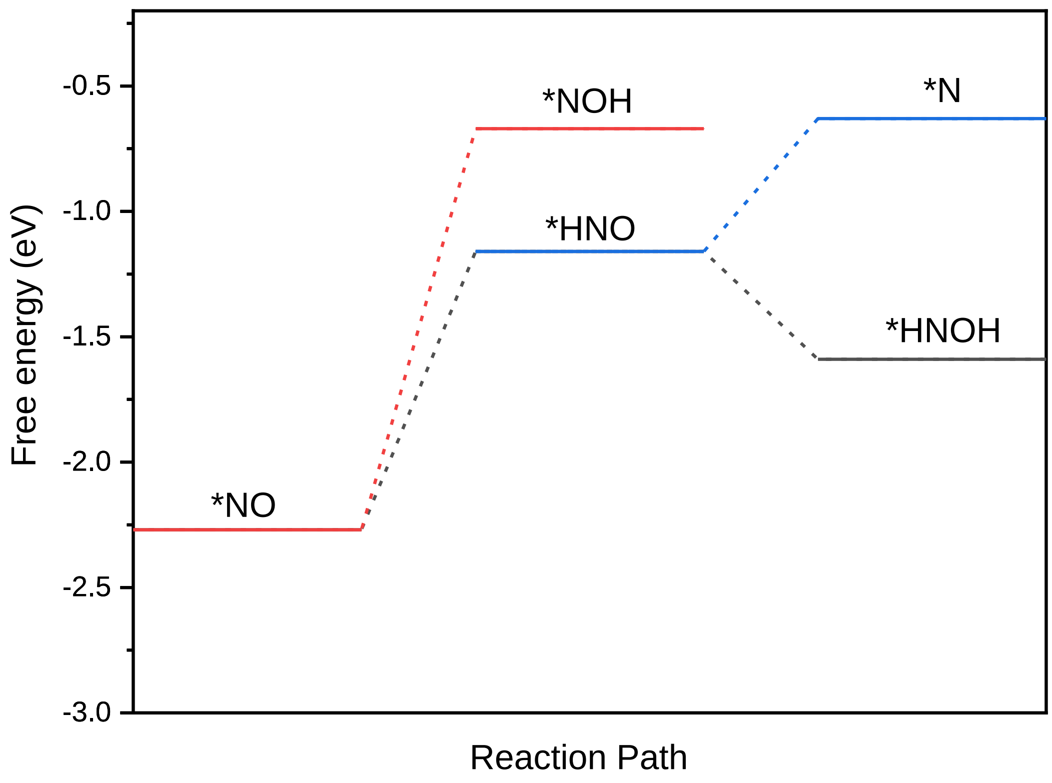

Supplementary Figure 51. Reaction free energy of first and second protonation of *NO on Co/Co_3O_4.

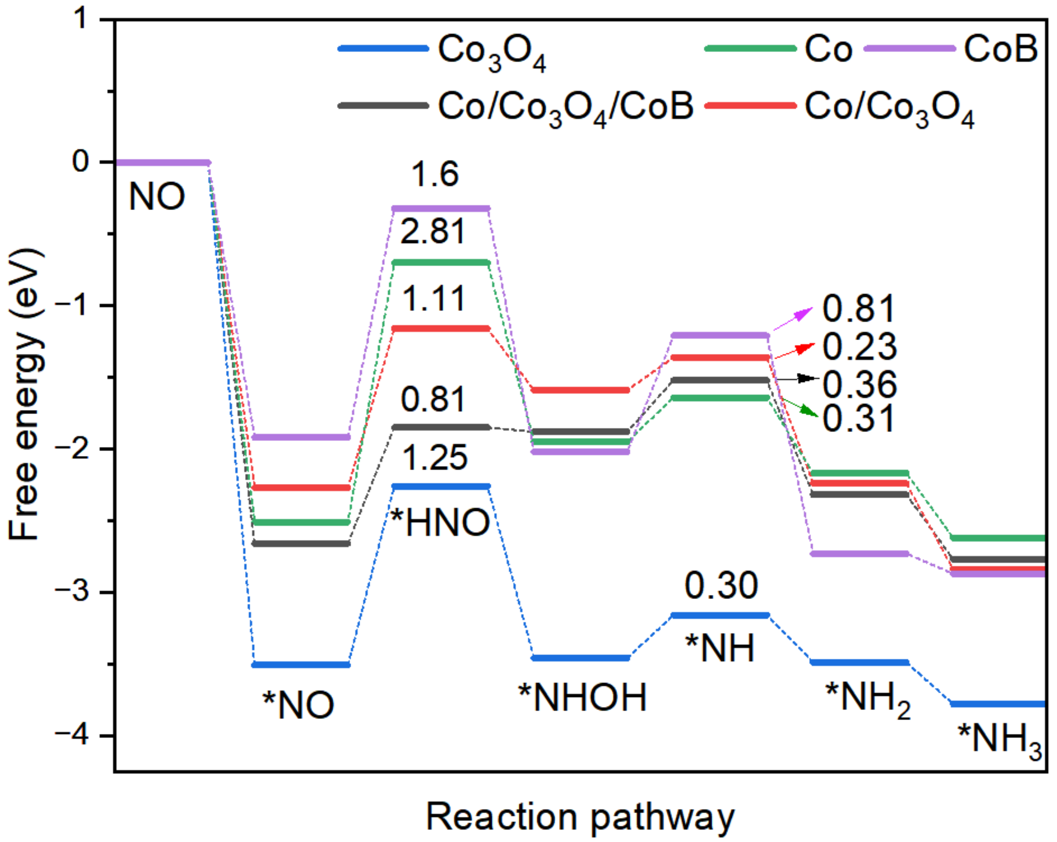

Supplementary Figure 52. The calculated NORR Gibbs free energy diagrams over Co_3O_4, Co, CoB, Co/Co_3O_4 and $Co/Co_3O_4/CoB$ at 0 V vs. RHE.

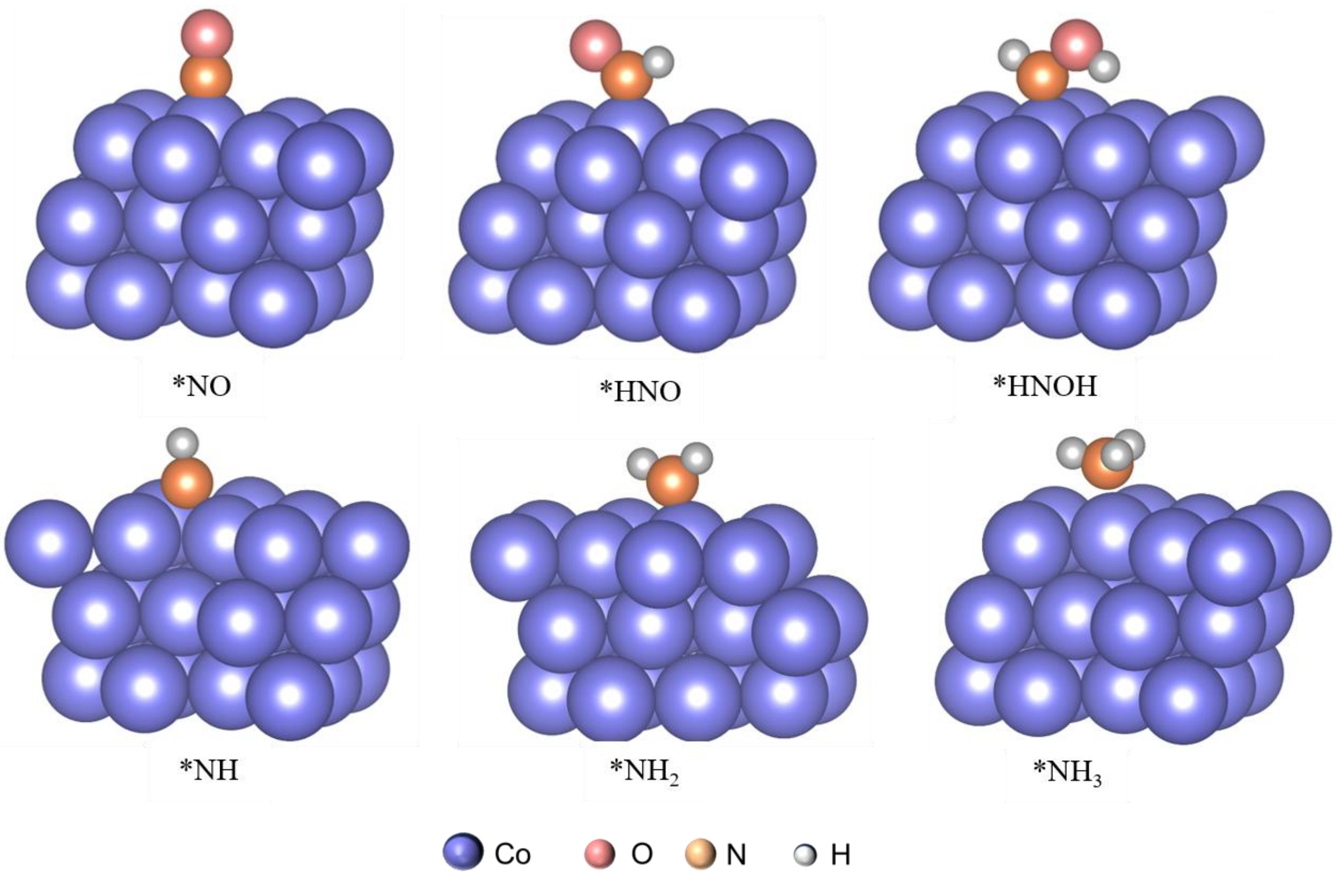

Supplementary Figure 53. Structural models showing NORR on Co.

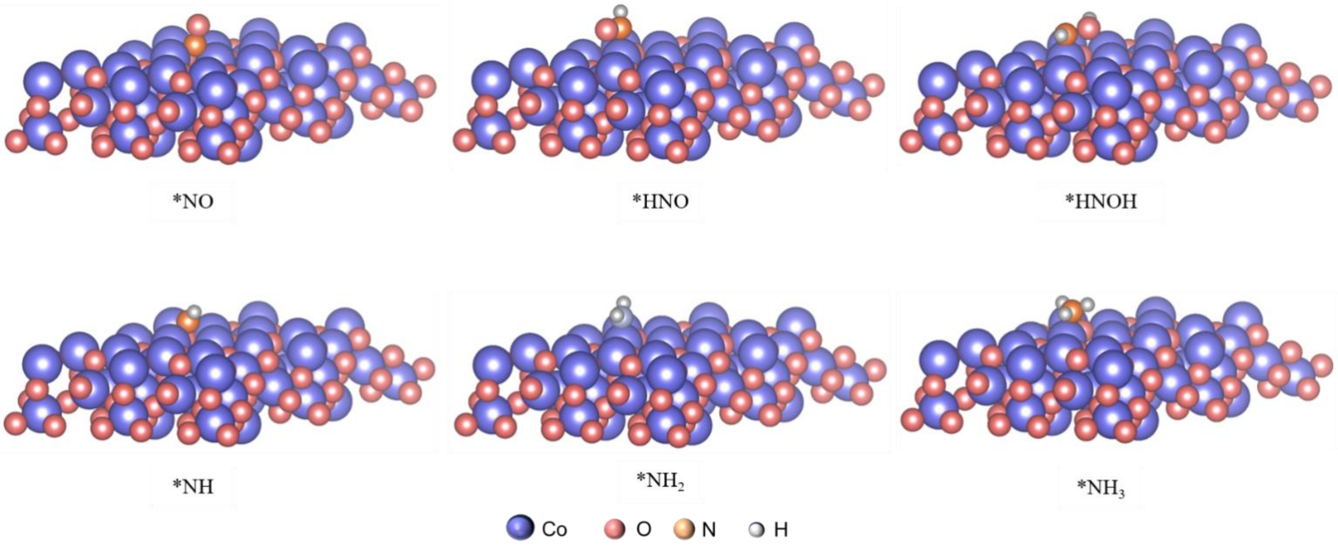

Supplementary Figure 54. Structural models showing NORR on Co_3O_4.

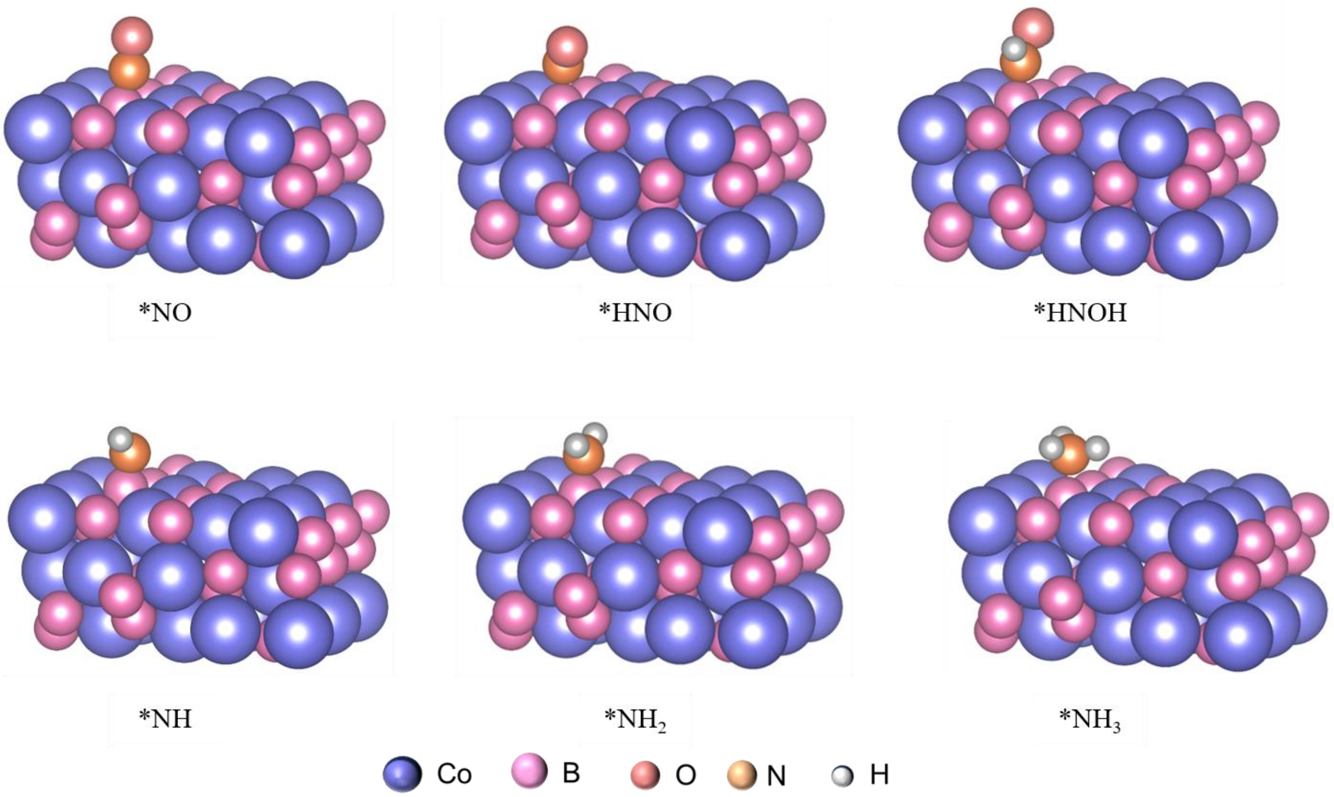

Supplementary Figure 55. Structural models showing NORR on CoB.

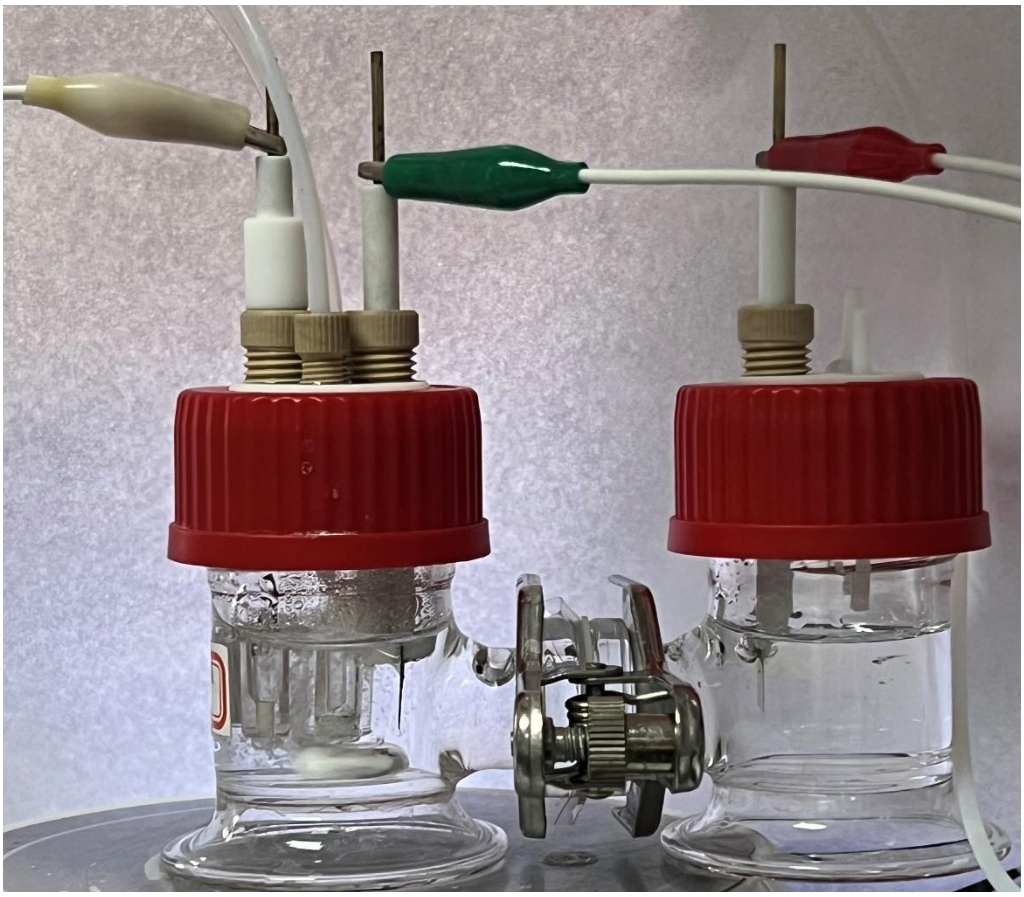

Supplementary Figure 56. The custom-built electrochemical cells for NORR.

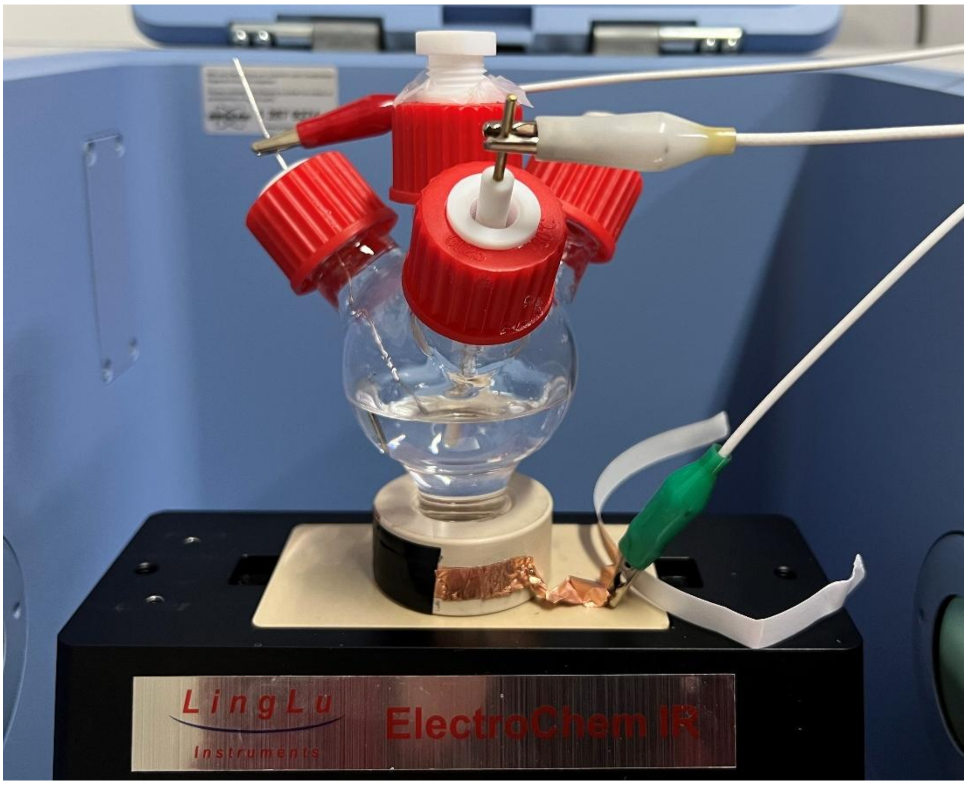

Supplementary Figure 57. The custom-built *in-situ* ATR-SEIRAS device for NORR.

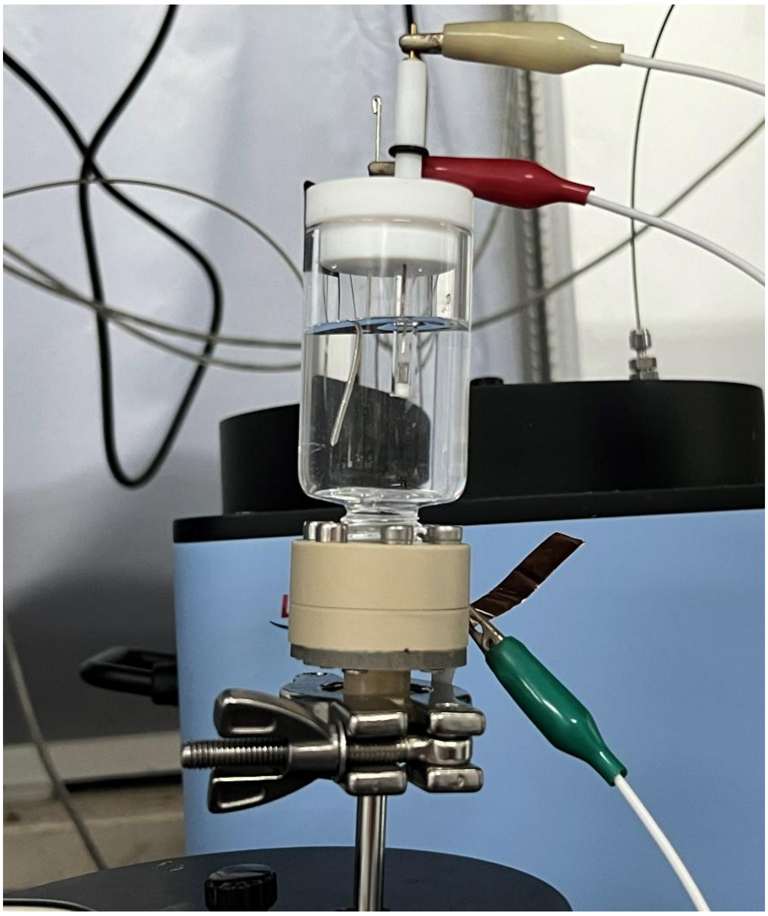

Supplementary Figure 58. The custom-built *in-situ* DEMS device for NORR

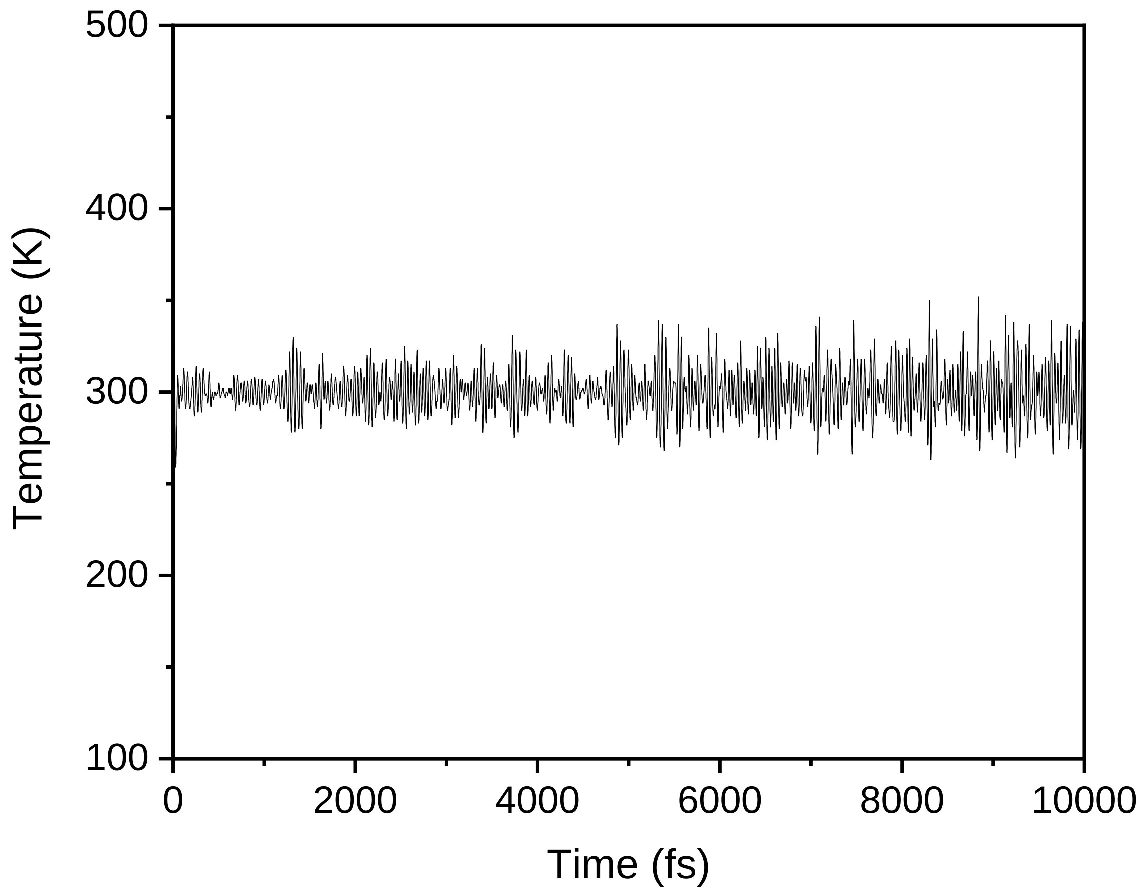

Supplementary Figure 59. Variations of temperature during the AIMD simulation for assessing the stability of Co/Co_3O_4/CoB model.

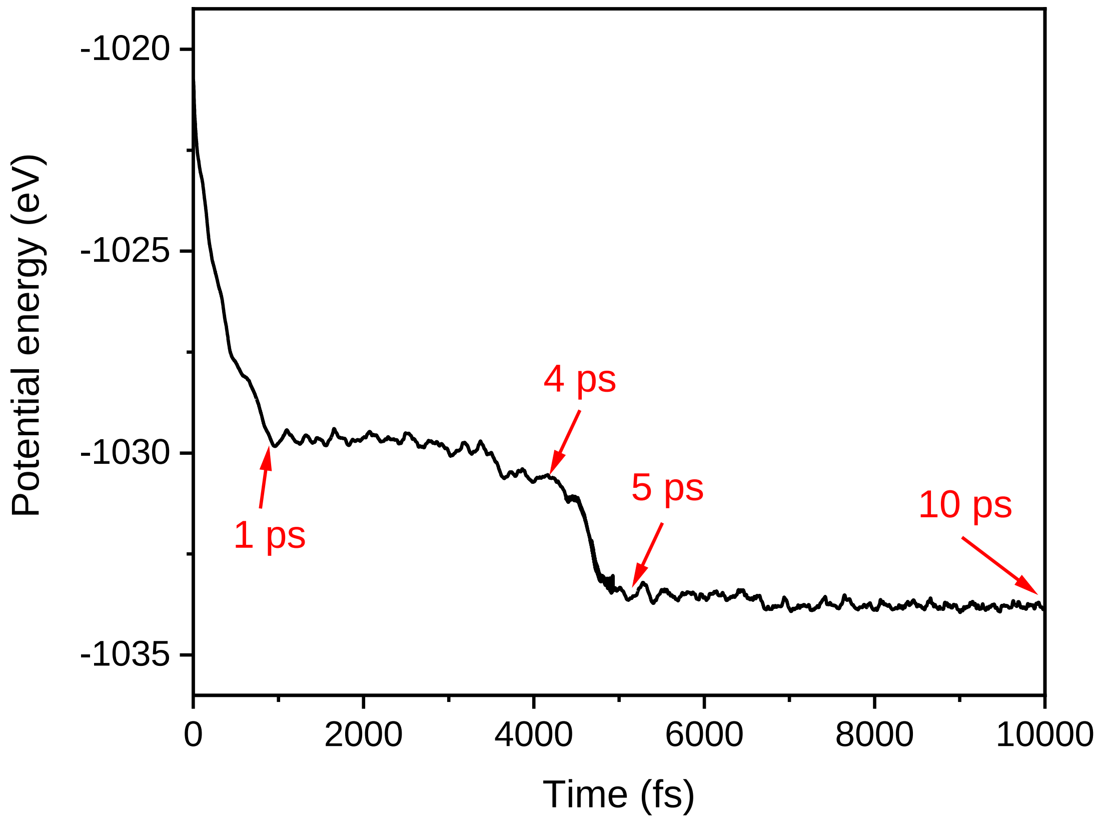

Supplementary Figure 60. Variations of potential energy during the AIMD simulation for assessing the stability of the Co/Co_3O_4/CoB model.

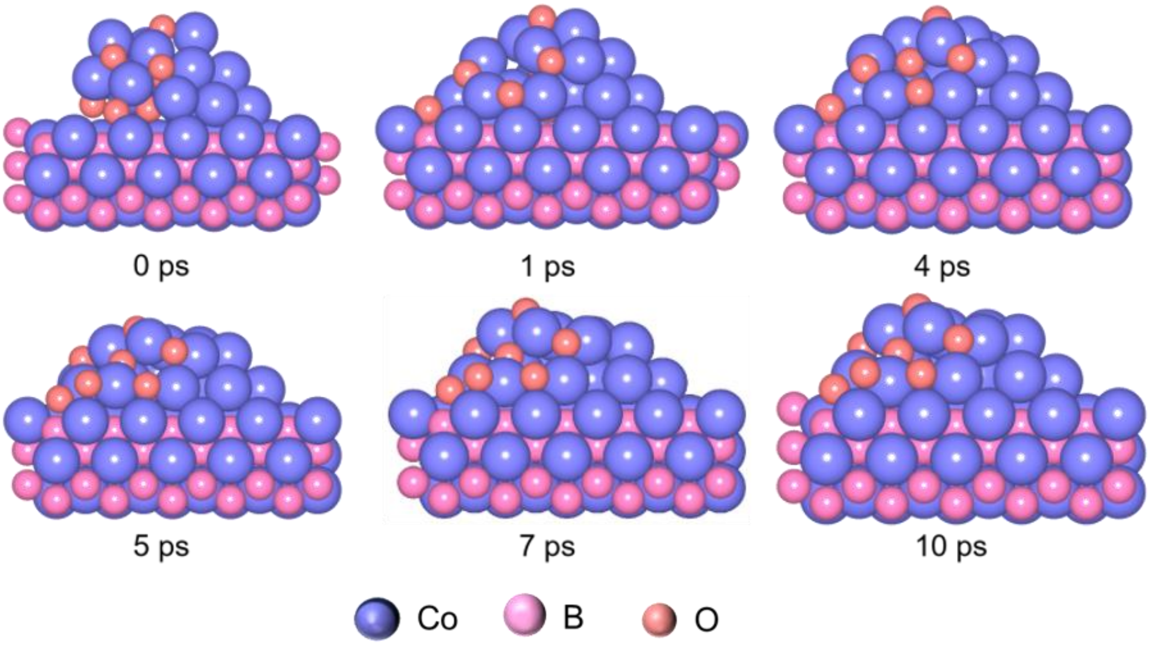

Supplementary Figure 61. The structure mode of $Co/Co_3O_4/CoB$ in 0 ps to 10 ps.

Supplementary Tables

Supplementary Table 1. The quantitative analysis of Bader charge over $Co/Co_3O_4/CoB$ and Co/Co_3O_4.

		**Total transferred electrons**	**The average number of electron transfers per Co atom**
$Co/Co_3O_4/CoB$	CoB	-2.36	-0.04
	Co_3O_4	4.59	0.51
	Co	-2.23	-0.22
Co/Co_3O_4	Co_3O_4	3.64	0.08
	Co	-3.64	-0.13

Supplementary Table 2. The FT-EXAFS fitting parameters of $Co/Co_3O_4/CoB$.

Shell	N	R(Å)	$\Delta\sigma^{2}$*10^{-3} (Å^2)	ΔE_0 (eV)	R-factor
Co-O	2.2	1.98	3.0	0.06	0.017
Co-B	2.3	2.21	3.0		
Co-Co	2.5	2.54	7.0		

Supplementary Table 3. The FT-EXAFS fitting parameters of Co/Co_3O_4.

Shell	N	R(Å)	$\Delta\sigma^{2*}10^{-3}$ (Å^2)	ΔE_0 (eV)	R-factor
Co-O	5.7	1.90	3.0	-10.4	0.02
Co-Co	1.3	2.43			
Co-Co	3.2	2.87			

Supplementary Table 4. Comparison of NH_3 yield rate and FE_{NH_3} of Co/Co_3O_4/CoB with the reported NORR electrocatalysts.

**Catalyst**	**Electrolyte**	**NH_3 yield rate ($\mu mol \cdot h^{-1} \cdot cm^{-2}$)**	**FE_{NH_3} (%)**	**Stability time (h)**	**V vs. RHE**	**References**
Co/Co_3O_4/CoB	0.1 M PBS	462.18	98.8	~100	-0.5	This work
hcp-Co	0.1 M Na_2SO_4	439.50	72.58	6	-0.6	[3]
Nb-SA/BNC	0.1 M Na_2SO_4	295.2	48.14	56	-0.9	[4]
Cu (111)	0.1 M Na_2SO_4	187.5	93.19	5	-0.59	[5]
a-$B_{2.6}C$@TiO_2/Ti	0.1 M Na_2SO_4	216.39	87.6	12	-0.9	[6]
Fe_1/MoS_{2-x}	0.1 M Na_2SO_4	288.2	82.5	15	-0.6	[7]
Sb_1/a-MoO_3	0.1 M Na_2SO_4	273.5	91.7	30	-0.6	[8]
Cu_1/MoS_2	0.1 M Na_2SO_4	337.5	90.6	20	-0.6	[9]
NiO/TM	0.1 M Na_2SO_4	125.29	90	12	-0.6	[10]
CoB/Co@C	0.5 M Na_2SO_4	315.4	~ 85	10	-0.6	[11]
Sb_2S_3	0.1 M Na_2SO_4	168.6	93.7	20	-0.7	[12]

Supplementary Table 5. The FE distribution of $Co/Co_3O_4/CoB$ and Co/Co_3O_4 at -0.5 V vs. RHE.

Catalysts	NH_3 (%)	NH_2OH (%)	Calculated H_2 (%)
$Co/Co_3O_4/CoB$	98.80	undetected	1.20
Co/Co_3O_4	91.72	undetected	8.72

Supplementary Table 6. Comparison of NH_3 yield rate and peak power density of various Zn-NO battery systems.

**Catalyst**	**Battery system**	**Power density (mW cm^{-2})**	**NH_3 yield rate**	**References**
$Co/Co_3O_4/CoB$	Metal-NO	10.56	1627.675 μg mg_{cat}^{-1} h^{-1}	This work
Hcp-Co	Metal-NO	4.66	247.8 μg mg_{cat}^{-1} h^{-1}	[3]
Nanoporous VN	Metal-NO	2.0	1077.1 μg mg_{cat}^{-1} h^{-1}	[13]
a-$B_{2.6}C$@TiO_2/Ti	Metal-NO	1.7	1125.2 μg mg_{cat}^{-1} h^{-1}	[6]
Ni_2P/CP	Metal-NO	1.53	62.05 μg mg_{cat}^{-1} h^{-1}	[14]
Fe_2O_3/TM	Metal-NO	1.18	145.28 μg mg_{cat}^{-1} h^{-1}	[15]
MoS_2/GF	Metal-NO	1.04	411.8 μg mg_{cat}^{-1} h^{-1}	[16]
NiO/TM	Metal-NO	0.88	288 μg mg_{cat}^{-1} h^{-1}	[10]
TiO_{2-x}/TP	Metal-NO	0.84	241.7 μg mg_{cat}^{-1} h^{-1}	[17]
CoPi/NPCS	Metal-N_2	0.49	14.7 μg mg_{cat}^{-1} h^{-1}	[18]
CoPi/HSNPC	Metal-N_2	0.31	11.62 μg mg_{cat}^{-1} h^{-1}	[19]
NbS_2	Metal-N_2	0.31	37.58 μg mg_{cat}^{-1} h^{-1}	[20]

Supplementary Table 7. The reaction energy, zero-point energy, and entropy corrections in the free energy calculations for H_2O dissociation over $Co/Co_3O_4/CoB$ and Co/Co_3O_4 surface.

		△E	TPZ	T△S	△G (eV)
$Co/Co_3O_4/CoB$	$*H_2O$	-1.21	0.71	0.13	-0.62
	*OH+*H	-1.19	0.54	0.08	-0.73
Co/Co_3O_4	$*H_2O$	-1.08	0.66	0.06	-0.49
	*OH+*H	-0.70	0.46	0.02	-0.26

Supplementary Table 8. The reaction energy, zero-point energy, and entropy corrections of each intermediate in the free energy calculations over $Co/Co_3O_4/CoB$, Co/Co_3O_4, CoB, Co_3O_4, and Co surface.

		△E	TPZ	T△S	△G (eV)
Co/Co_3O_4	*NO	-2.37	0.26	0.16	-2.27
	*NOH	-1.07	0.54	0.14	-0.67
	*HNO	-1.58	0.51	0.09	-1.16
	*N	-0.65	0.09	0.07	-0.63
	*HNOH	-2.29	0.84	0.14	-1.59
	*NH	-1.72	0.49	0.14	-1.36
	$*NH_2$	-2.90	0.74	0.08	-2.24
	$*NH_3$	-3.81	1.11	0.14	-2.84
$Co/Co_3O_4/CoB$	*NO	-2.80	0.24	0.10	-2.66
	*NOH	-1.73	0.53	0.10	-1.30
	*HNO	-2.24	0.53	0.13	-1.85
	*N	-0.90	0.12	0.03	-0.82
	*HNOH	-2.58	0.81	0.12	-1.88
	*NH	-1.84	0.40	0.07	-1.52
	$*NH_2$	-2.95	0.70	0.07	-2.32
	$*NH_3$	-3.75	1.11	0.13	-2.77
CoB	*NO	-2.05	0.24	0.11	-1.92
	*HNO	-0.79	0.52	0.05	-0.32
	*HNOH	-2.71	0.84	0.15	-2.02
	*NH	-1.53	0.34	0.03	-1.21
	$*NH_2$	-3.35	0.75	0.13	-2.73
	$*NH_3$	-3.91	1.14	0.09	-2.87
Co_3O_4	*NO	-3.64	0.22	0.08	-3.51
	*HNO	-2.63	0.51	0.14	-2.26
	*HNOH	-4.19	0.82	0.09	-3.46
	*NH	-3.44	0.35	0.07	-3.16
	$*NH_2$	-4.08	0.70	0.11	-3.49
	$*NH_3$	-4.75	1.08	0.10	-3.78
Co	*NO	-2.68	0.21	0.04	-2.51
	*HNO	-1.12	0.53	0.12	-0.70
	*HNOH	-2.64	0.84	0.15	-1.95
	*NH	-1.92	0.37	0.09	-1.64
	$*NH_2$	-2.74	0.69	0.11	-2.17
	$*NH_3$	-3.60	1.07	0.10	-2.62

Supplementary References

1. Han, H. et al. Advantageous crystalline–amorphous phase boundary for enhanced electrochemical water oxidation. *Energy Environ. Sci.* **12**, 2443-2454 (2019).
2. Masa, J. et al. Amorphous cobalt boride (Co_2B) as a highly efficient nonprecious catalyst for electrochemical water splitting: oxygen and hydrogen evolution. *Adv. Energy Mater.* **6**, 1502313 (2016).
3. Wang, D. et al. Hexagonal cobalt nanosheets for high-performance electrocatalytic NO reduction to NH_3. *J. Am. Chem. Soc.* **145**, 6899-6904 (2023).
4. Peng, X. et al. Ambient electrosynthesis of ammonia with efficient denitration. *Nano Energy.* **78**, 105321 (2020).
5. Xiao, L. et al. Identification of Cu(111) as superior active sites for electrocatalytic NO reduction to NH_3 with high single-pass conversion efficiency. *Angew. Chem. Int. Ed.* **63**, e202319135 (2024).
6. Liang, J. et al. Amorphous boron carbide on titanium dioxide nanobelt arrays for high-efficiency electrocatalytic NO reduction to NH_3. *Angew. Chem. Int. Ed.* **61**, e202202087 (2022).
7. Chen, K. et al. Atomically Fe-doped MoS_{2-x} with Fe-Mo dual sites for efficient electrocatalytic NO reduction to NH_3. *Appl. Catal. B: Environ.* **324**, 122241 (2023).
8. Chen, K. et al. p-Block antimony single-atom catalysts for nitric oxide electroreduction to ammonia. *ACS Energy Lett.* **8**, 1281-1288 (2023).
9. Chen, K., Zhang, G., Li, X., Zhao, X. & Chu, K. Electrochemical NO reduction to NH_3 on Cu single atom catalyst. *Nano Res.* **16**, 5857-5863 (2023).
10. Liu, P. et al. High-performance NH_3 production via NO electroreduction over a NiO nanosheet array. *Chem. Commun.* **57**, 13562-13565 (2021).
11. Wu, B. et al. Boron-modulated electronic-configuration tuning of cobalt for enhanced nitric oxide fixation to ammonia. *Nano Lett.* **23**, 7120-7128 (2023).
12. Chen, K., Zhang, Y., Du, W., Guo, Y. & Chu, K. Atomically isolated and unsaturated Sb sites created on Sb_2S_3 for highly selective NO electroreduction to NH_3. *Inorg. Chem. Front.* **10**, 2708-2715 (2023).
13. Lv, X.-W., Liu, Y., Wang, Y.-S., Liu, X.-L. & Yuan, Z.-Y. Encapsulating vanadium nitride nanodots into N,S-codoped graphitized carbon for synergistic electrocatalytic nitrogen reduction and aqueous Zn-N_2 battery. *Appl. Catal. B: Environ.* **280**, 119434 (2021).
14. Mou, T. et al. High-efficiency electrohydrogenation of nitric oxide to ammonia on a Ni_2P nanoarray under ambient conditions. *J. Mater. Chem. A.* **9**, 24268-24275 (2021).
15. Liang, J. et al. Coupling denitrification and ammonia synthesis via selective electrochemical reduction of nitric oxide over Fe_2O_3 nanorods. *J. Mater. Chem. A.* **10**, 6454-6462 (2022).
16. Zhang, L. et al. High-performance electrochemical NO reduction into NH_3 by MoS_2 nanosheet. *Angew. Chem. Int. Ed.* **60**, 25263-25268 (2021).
17. Li, Z. et al. Defective TiO_{2-x} for high-performance electrocatalytic NO reduction toward ambient NH_3 production. *Small* **19**, 2300291 (2023).
18. Ren, J.-T., Chen, L., Wang, H.-Y. & Yuan, Z.-Y. Aqueous rechargeable Zn–N_2 battery assembled by bifunctional cobalt phosphate nanocrystals-loaded carbon nanosheets for simultaneous NH_3 production and power generation. *ACS Appl. Mater. Inter* **13**, 12106-12117 (2021).
19. Ren, J.-T., Chen, L., Liu, Y. & Yuan, Z.-Y. Hollow cobalt phosphate microspheres for sustainable electrochemical ammonia production through rechargeable Zn–N_2 batteries. *J. Mater. Chem. A.* **9**, 11370-11380 (2021).
20. Wang, H. et al. Exfoliated metallic niobium disulfate nanosheets for enhanced electrochemical ammonia synthesis and Zn-N_2 battery. *Appl. Catal. B: Environ.* **270**, 118892 (2020).
