# Supplementary material for: Boosted charge and proton transfer over ternary Co/Co3O4/CoB for electrochemical nitric oxide reduction to ammonia
Source: Nat Commun. 2025 May 26;16:4874. doi: 10.1038/s41467-025-60043-6 (PMC12106605; doi:10.1038/s41467-025-60043-6)
Supplement: Supplementary file 2 — Description Of Additional Supplementary File [file 41467_2025_60043_MOESM2_ESM.pdf]

Description of Additional supplementary file

Supplementary Data 1 provides the atomic coordinates of the optimized computational models of Co/Co3O4/CoB, Co/Co3O4, Co/Co3O4/CoB with adsorbed NO, and Co/Co3O4 with adsorbed NO.

Supplementary Data 2 provides the structure mode of Co/Co3O4/CoB in 0 ps to 10 ps for the molecular dynamics trajectories.
